# Cardiomyocyte-specific knockout of ADAM17 alleviates doxorubicin-induced cardiomyopathy via inhibiting TNFα–TRAF3–TAK1–MAPK axis

**DOI:** 10.1038/s41392-024-01977-z

**Published:** 2024-10-16

**Authors:** Lin Xie, Fei Xue, Cheng Cheng, Wenhai Sui, Jie Zhang, Linlin Meng, Yue Lu, Wenjing Xiong, Peili Bu, Feng Xu, Xiao Yu, Bo Xi, Lin Zhong, Jianmin Yang, Cheng Zhang, Yun Zhang

**Affiliations:** 1 State Key Laboratory for Innovation and Transformation of Luobing Theory; Key Laboratory of Cardiovascular Remodeling and Function Research, Chinese Ministry of Education, Chinese National Health Commission and Chinese Academy of Medical Sciences; Department of Cardiology, Qilu Hospital of Shandong University, Jinan, China; 2 Department of Cardiology, Shengjing Hospital of China Medical University, Shenyang, Liaoning Province China; 3 Department of Emergency Medicine, Chest Pain Center, Shandong Provincial Clinical Research Center for Emergency and Critical Care Medicine, Qilu Hospital, Shandong University, Jinan, China; 4 Key Laboratory Experimental Teratology of the Ministry of Education, Department of Physiology, School of Basic Medical Sciences, Cheeloo College of Medicine, Shandong University, Jinan, China; 5 Department of Epidemiology, School of Public Health, Cheeloo College of Medicine, Shandong University, Jinan, China; 6 Department of Cardiology, The Affiliated Yantai Yuhuangding Hospital of Qingdao University, Yantai, China; 7 Cardiovascular Disease Research Center of Shandong First Medical University, Central Hospital Affiliated to Shandong First Medical University, Jinan, China

**Keywords:** Cardiology, Cardiovascular diseases

## Abstract

The pathogenesis of doxorubicin-induced cardiomyopathy remains unclear. This study was carried out to test our hypothesis that ADAM17 aggravates cardiomyocyte apoptosis induced by doxorubicin and inhibition of ADAM17 may ameliorate doxorubicin-induced cardiomyopathy. C57BL/6J mice were intraperitoneally injected with a cumulative dose of doxorubicin to induce cardiomyopathy. Cardiomyocyte-specific ADAM17-knockout (A17^α-MHCKO^) and ADAM17-overexpressing (AAV9-oeA17) mice were generated. In addition, RNA sequencing of the heart tissues in different mouse groups and in vitro experiments in neonatal rat cardiomyocytes (NRCMs) receiving different treatment were performed. Mouse tumor models were constructed in A17^fl/fl^ and A17^α-MHCKO^ mice. In addition, cardiomyocyte-specific TRAF3-knockdown and TRAF3-overexpressing mice were generated. ADAM17 expression and activity were markedly upregulated in doxorubicin-treated mouse hearts and NRCMs. A17^α-MHCKO^ mice showed less cardiomyocyte apoptosis induced by doxorubicin than A17^fl/fl^ mice, and cardiomyocyte ADAM17 deficiency did not affect the anti-tumor effect of doxorubicin. In contrast, AAV9-oeA17 mice exhibited markedly aggravated cardiomyocyte apoptosis relative to AAV9-oeNC mice after doxorubicin treatment. Mechanistically, doxorubicin enhanced the expression of transcription factor C/EBPβ, leading to increased expression and activity of ADAM17 in cardiomyocyte, which enhanced TNF-α shedding and upregulated the expression of TRAF3. Increased TRAF3 promoted TAK1 autophosphorylation, resulting in activated MAPKs pathway and cardiomyocyte apoptosis. ADAM17 acted as a positive regulator of cardiomyocyte apoptosis and cardiac remodeling and dysfunction induced by doxorubicin by upregulating TRAF3/TAK1/MAPKs signaling. Thus, targeting ADAM17/TRAF3/TAK1/MAPKs signaling holds a promising potential for treating doxorubicin-induced cardiotoxicity.

## Introduction

Doxorubicin as a commonly used anthracycline has become the cornerstone of chemotherapy in a wide range of cancers owing to its high efficacy.^1^ However, clinical applications of doxorubicin are limited mainly due to its toxic effects on myocardium, such as cardiomyopathy, heart failure and arrhythmias, but the pathogenic mechanism of doxorubicin-induced cardiomyopathy is poorly understood.^2^ Several mechanisms have been proposed for the pathogenesis of cardiomyopathy induced by doxorubicin, including drug intercalation into DNA, inhibition of macromolecule synthesis, initiation of DNA damage via inhibition of topoisomerase II, autophagy disorder, ferroptosis, reactive oxygen species (ROS) overproduction and cardiomyocyte apoptosis.^3,4^ In particular, cardiomyocyte apoptosis induced by oxidative stress and ROS production through extrinsic or intrinsic pathways after doxorubicin treatment has received great attention.^5,6^ However, simple application of antioxidants did not prevent doxorubicin-induced cardiomyopathy,^7^ suggesting that this pathology may involve more complex mechanisms than expected. It has been reported that doxorubicin may directly promote cardiomyocyte apoptosis without the involvement of ROS production and peroxide stress.^8^ Furthermore, the serum level of TNF-α was increased in patients receiving doxorubicin chemotherapy, and TNF-α expression in the myocardium was also enhanced in mice receiving doxorubicin treatment.^9^ It has been confirmed that TNF-α may stimulate the expression of several pro-apoptotic proteins such as Bax,^7^ thus promoting cardiomyocyte apoptosis. As with other forms of dilated cardiomyopathy, doxorubicin-induced cardiotoxicity may exhibit extensive fibrosis and scattered cardiomyocytes with vacuolar degeneration, and necrotic cardiomyopathy may be present, although less common.^10^ Therefore, exploration of TNF-α-related pathogenetic mechanism and discovery of novel therapeutic targets of doxorubicin-induced cardiomyopathy are highly warranted.

Members of the A Disintegrin and Metalloproteinase (ADAM) family have been recognized as major proteinases for ectodomain shedding. ADAMs are type I transmembrane proteins consisting of an N-terminal signal sequence, followed by a pro-domain, metalloproteinase domain, a disintegrin domain, an EGF-like domain, a single transmembrane domain and a cytoplasmic portion.^11^ ADAM17 is known as tumor necrosis factor α converting enzyme (TACE)^12^ that converts transmembrane TNF-α (tmTNF-α) to soluble TNF-α (sTNF-α), and the cleavage of TNF-α by ADAM17 is a prerequisite for pro-inflammatory TNF-α activity,^13^ which raises a possibility that inhibition of ADAM17 may exert a beneficial effect on disease processes where TNF-α plays an essential role. Recent studies by our team demonstrated that ADAM17 knockdown affected autophagy through regulating the AMPK signaling pathway, thereby alleviating cardiomyocyte apoptosis and improving diabetic cardiomyopathy.^14^ However, the relationship between cardiomyocyte ADAM17 and doxorubicin-induced cardiomyopathy is unclear. The therapeutic effect and its mechanism of cardiomyocyte-specific ADAM17 deficiency on doxorubicin-induced cardiomyopathy, and whether it interferes with the anti-tumor effects of doxorubicin have not been reported.

Members of the tumor necrosis factor receptor (TNFR) associated factor (TRAF) family, including TRAF1–7, are key adapter molecules that transmit signals to downstream factors, and are involved in many biological processes such as cell survival.^15^ TRAF3 shares a well-defined functional TRAF domain with other TRAFs, which enables TRAF3 an adaptor activity that facilitates the formation of intracellular domain complexes of transmembrane receptors and signaling to the downstream cascade.^16^ Previous studies have shown that TRAF3 was involved in many pathophysiological processes, such as acting as a positive regulator of pathological cardiac hypertrophy, and cardiomyocyte-specific knockout of TRAF3 significantly alleviates pathological cardiac hypertrophy, fibrosis, and dysfunction in mice.^17^ In addition, TRAF3 binds to transforming growth factor-β (TGF-β) activated kinase 1 (TAK1) during hepatic ischemia/reperfusion (I/R) injury, enhances downstream MAPKs pathway activation, and accelerates apoptosis during I/R injury.^18^ TRAF3 mediates neuronal apoptosis in early brain injury after subarachnoid hemorrhage through the targeted TAK1-dependent MAPKs pathway.^19^ These studies suggest that TRAF3 may play an important regulatory role in the process of pathological cell apoptosis. However, the role of TRAF3 in doxorubicin-induced cardiomyopathy remains elusive and merits exploration.

TAK1, originally identified as a mitogen-activated protein kinase kinase kinase (MAP3K),^20^ is activated by a number of signaling molecules, including TGF-β, TNF-α and interleukin-1(IL-1).^21^ Activation of TAK1 leads to phosphorylation and degradation of IκB, thereby activating NF-κB.^22^ In addition, activated TAK1 also phosphorylates and activates MAPKKs, resulting in the activation of MAPKs such as JNK, P38 MAPK and ERK.^23^ Previous studies have pointed to TRAF3 as a potential therapeutic target for ischemic stroke by interacting with TAK1 and enhancing TAK1 phosphorylation and activation.^24^ TRAF3 has been shown to promote liver damage and inflammation through TAK1-dependent activation of JNK and NF-κB pathways.^18^ Hepatocyte TRAF3 binds to TAK1, inducing TAK1 ubiquitination and subsequent autophosphorylation, thereby enhancing activation of downstream IKKβ–NF-κB and MKK–JNK signaling cascades, which are essential for regulating hepatic steatosis.^25^ However, the role of TAK1 and its downstream MAPKs pathway in doxorubicin-induced cardiomyopathy has not been reported. Based on the above scientific questions, we hypothesize that cardiomyocyte ADAM17 aggravates cardiomyocyte apoptosis induced by doxorubicin by regulating TRAF3–TAK1–MAPKs pathways, and inhibition of ADAM17 may ameliorate doxorubicin-induced cardiomyopathy without affecting the anti-tumor effect of doxorubicin. This hypothesis was tested via a number of in vivo and in vitro experiments in the present study.

## Results

### Doxorubicin impairs cardiac function and upregulates ADAM17 expression in mouse hearts and NRCMs

**Fig. 1:**
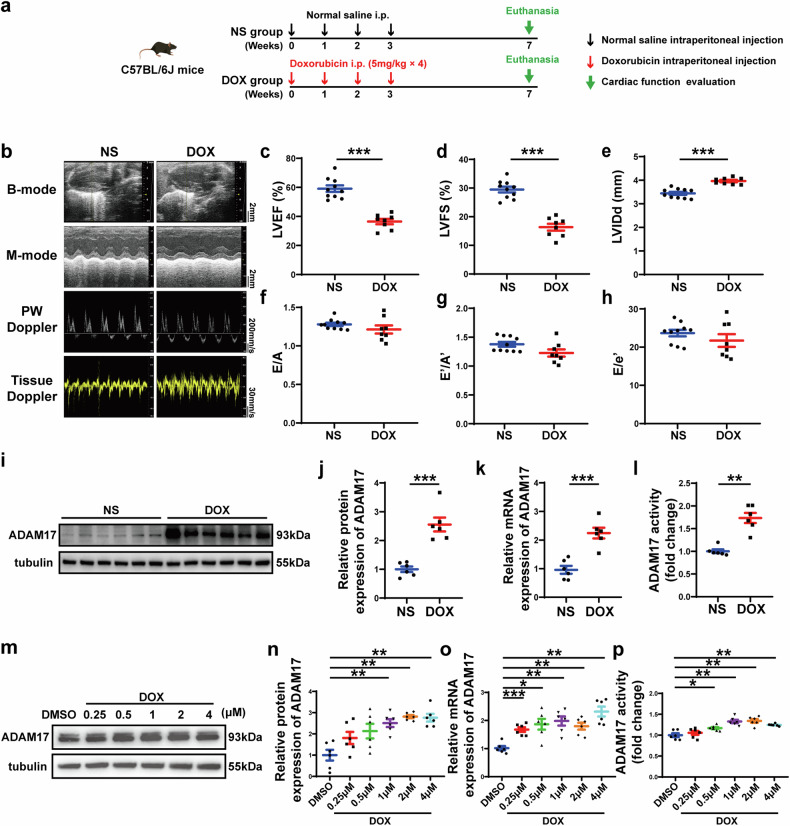
Echocardiographic measurements and ADAM17 protein expression in mice receiving different treatment. **a** Experiment timeline in vivo. **b** Representative echocardiographic images in mice showing B-mode echocardiogram (scale bar = 2 mm), M-mode echocardiogram (scale bar = 2 mm), pulse-wave Doppler tracing (PW) (scale bar = 200 mm/s), and tissue Doppler tracing (scale bar = 30 mm/s). **c** Comparison of left ventricular ejection fraction (LVEF) between mice treated with normal saline (NS) and doxorubicin (DOX) (*n* = 8–10 in each group). **d** Comparison of left ventricular fractional shortening (LVFS) between mice treated with normal saline (NS) and doxorubicin (DOX) (*n* = 8–10 in each group). **e** Comparison of left ventricular end-diastolic diameter (LVIDd) between mice treated with normal saline (NS) and doxorubicin (DOX) (*n* = 8–10 in each group). **f** Comparison of the ratio of early to late diastolic mitral flow velocities (E/A) between mice treated with normal saline (NS) and doxorubicin (DOX) (*n* = 8–10 in each group). **g** Comparison of the ratio of early to late diastolic mitral annular velocities (E′/A′) between mice treated with normal saline (NS) and doxorubicin (DOX) (*n* = 8–10 in each group). **h** Comparison of the ratio of early diastolic mitral flow to early diastolic mitral annulus velocity (E/E′) between mice treated with normal saline (NS) and doxorubicin (DOX) (*n* = 8–10 in each group). **i** Representative western blot images of ADAM17 protein expression in the myocardium of NS- and DOX-treated mice. **j** Comparison of ADAM17 protein expression in the myocardium of NS- and DOX-treated mice (*n* = 6 in each group). **k** Comparison of ADAM17 mRNA expression in the myocardium of NS- and DOX-treated mice (*n* = 6 in each group). **l** Comparison of ADAM17 activity in the myocardium of NS- and DOX-treated mice (*n* = 6 in each group). **m** Representative western blot images of ADAM17 protein expression in NRCMs treated with DMSO, or DOX at different concentrations, respectively, for 24 h. **n** Comparison of ADAM17 protein expression in NRCMs treated with DMSO, or DOX at different concentrations, respectively, for 24 h (*n* = 6 in each group). **o** Comparison of ADAM17 mRNA expression in NRCMs treated with DMSO, or DOX at different concentrations, respectively, for 24 h (*n* = 6 in each group). **p** Comparison of ADAM17 activity in the NRCMs treated with DMSO, or DOX at different concentrations, respectively, for 24 h (*n* = 6 in each group). Values shown were mean and SEM. Unpaired two-tailed Student’s *t*-tests were applied in (**c**–**f**, **h**, and **j**–**l**). Mann–Whitney *U* test was applied in (**g**). One-way ANOVA were applied in (**n**–**p**). *p < 0.05; **p < 0.01; ***p < 0.001

To investigate the role of ADAM17 in doxorubicin-induced cardiac injury, a mouse model of doxorubicin-induced cardiomyopathy was established in the first proportion of the in vivo experiments (Fig. 1a), where doxorubicin was continuously administered to C57BL/6J male mice, once a week for 4 weeks, to achieve chronic cardiotoxicity. Four weeks after the last dose of doxorubicin, the cardiac systolic function of mice injected with doxorubicin deteriorated as manifested as declined LVEF and LVFS, and enlarged LVIDd (Fig. 1b–e), as compared with the NS group. However, there was no significant difference in the ratio of E/A, E′/A′ and E/E′ (Fig. 1f–h) between the two groups of mice, suggesting that the effect of doxorubicin on cardiac diastolic function was less significant than that on cardiac systolic function. Next, we evaluated the effect of doxorubicin on ADAM17 expression in the mouse myocardium. Western blot and RT-PCR analysis demonstrated that myocardial ADAM17 protein and mRNA expression levels increased significantly in the doxorubicin-treated mouse relative to the NS group (Fig. 1i–k). ADAM17 activity was higher in the heart tissue of doxorubicin-injected mice than in that of normal saline-injected mice (Fig. 1l). In addition, immunofluorescence co-localization staining showed that ADAM17 was abundantly expressed in cardiomyocytes (Supplementary Fig. 1a). Compared with the NS group, ADAM17 expression in cardiomyocytes in the DOX group was significantly upregulated (Supplementary Fig. 1b, c). In neonatal rat cardiomyocytes (NRCMs) stimulated with various concentrations of doxorubicin, ADAM17 protein, mRNA levels and ADAM17 activity increased in a concentration-dependent manner (Fig. 1m–p). These results indicated that cardiomyocyte ADAM17 may be involved in the pathogenesis of doxorubicin-induced cardiotoxicity.

### Cardiomyocyte-specific knockout of ADAM17 alleviates cardiac dysfunction and remodeling induced by doxorubicin

**Fig. 2:**
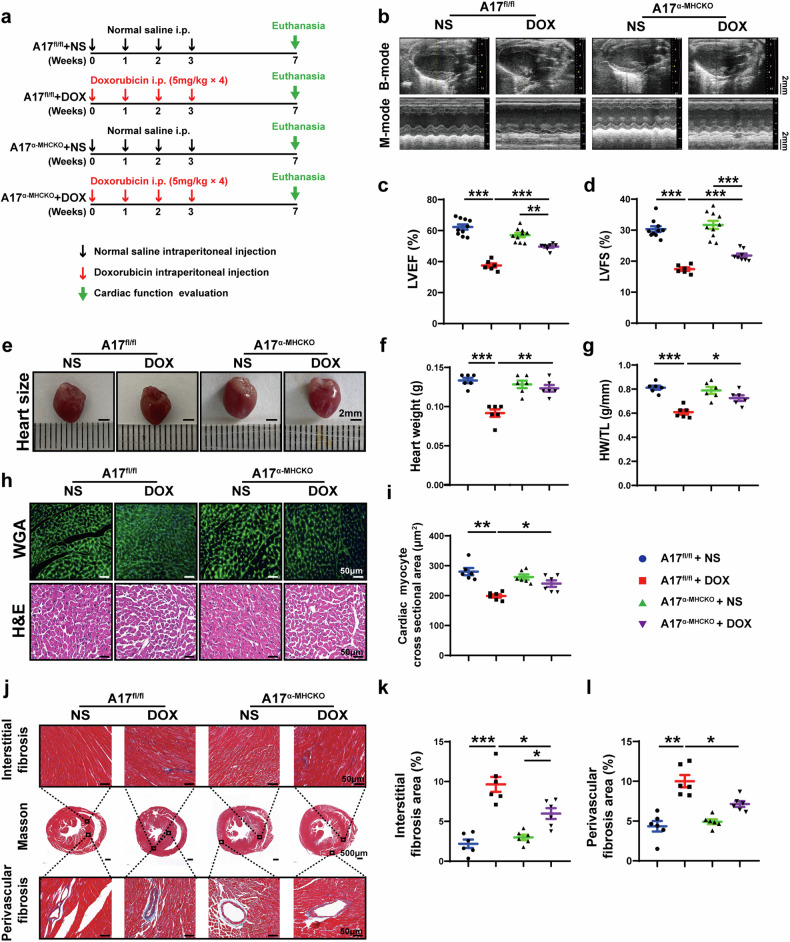
Echocardiographic measurements and histological staining in ADAM17^fl/fl^ and ADAM17^α-MHCKO^ mice treated with NS or DOX. **a** Experiment timeline in vivo. **b** Representative echocardiographic images (scale bar = 2 mm) showing B-mode and M-mode echocardiograms in four groups of mice. **c** Comparison of left ventricular ejection fraction (LVEF) among four groups of mice (*n* = 6–10 in each group). **d** Comparison of left ventricular fractional shortening (LVFS) among four groups of mice (*n* = 6–10 in each group). **e** Representative anatomical images of heart size (scale bar = 2 mm) in four groups of mice. **f** Comparison of heart weight among four groups of mice (*n* = 6 in each group). **g** Comparison of heart weight/tibial length (HW/TL) ratio among four groups of mice (*n* = 6 in each group). **h** Representative WGA and H&E staining of myocardial cross-sections (scale bar = 50 μm) in four groups of mice. **i** Comparison of cardiac myocyte cross-sectional area measured by WGA staining (*n* = 6 in each group). **j** Representative Masson’s trichrome staining of myocardial interstitial and perivascular fibrosis in four groups of mice. **k** Comparison of interstitial fibrosis area among four groups of mice (*n* = 6 in each group). **l** Comparison of perivascular fibrosis area among four groups of mice (*n* = 6 in each group). Values shown were mean and SEM. One-way ANOVA were applied in (**c**, **d**, **f**, **g**, **i**, **k** and **l**). *p < 0.05; **p < 0.01; ***p < 0.001

Since ADAM17 expression was upregulated in the cardiomyocytes of doxorubicin-treated mice, we crossed ADAM17 flox/flox mice with cardiomyocyte-specific α-MHC-Cre mice to obtain ADAM17 cardiomyocyte-specific knockout mice (ADAM17^α-MHCKO^), as displayed in Supplementary Fig. 2a, b. Western blot and RT-PCR showed that, compared with A17^fl/fl^ mice, the protein and mRNA expression level of ADAM17 in A17^α-MHCKO^ mouse heart decreased by about 70% and about 80%, respectively (Supplementary Fig. 2c–e). In addition, the ADAM17 activity of ADAM17 in A17^α-MHCKO^ mouse heart decreased by about 70% relative to that of A17^fl/fl^ mice (Supplementary Fig. 2f). To ascertain the role of ADAM17 in doxorubicin-induced cardiomyopathy, we selected A17^fl/fl^ and A17^α-MHCKO^ male mice at 8 weeks of age and injected doxorubicin into these mice in the second proportion of in vivo experiments (Fig. 2a). No significant difference in the survival rate was observed between A17^α-MHCKO^ and A17^fl/fl^ mice who received doxorubicin treatment (Supplementary Fig. 2g) although the survival rate tended to be higher in the former than the latter group. However, LVEF and LVFS were significantly higher in the A17^α-MHCKO^ mice than in the A17^fl/fl^ mice after doxorubicin treatment (Fig. 2b–d). On the contrary, heart weight and the ratio of heart weight to tibia length (HW/TL) were decreased in A17^fl/fl^ mice receiving doxorubicin injection versus A17^fl/fl^ mice receiving NS, while these parameters were improved in A17^α-MHCKO^ mice (Fig. 2e–g). Furthermore, WGA staining showed that myocardial cell cross-sectional area decreased in doxorubicin-injected A17^fl/fl^ mice, which was improved in A17^α-MHCKO^ mice after doxorubicin injection (Fig. 2h, i). As cardiac fibrosis is a typical feature of doxorubicin-induced chronic cardiac injury, Masson staining showed increased interstitial and perivascular fibrosis in the heart of doxorubicin-injected A17^fl/fl^ mice, which was again substantially ameliorated in A17^α-MHCKO^ mice receiving doxorubicin injection (Fig. 2j–l). These results suggested that cardiomyocyte-specific knockout of ADAM17 alleviated cardiac systolic dysfunction and fibrosis induced by doxorubicin.

### ADAM17 overexpression aggravates doxorubicin-induced cardiac dysfunction and remodeling

**Fig. 3:**
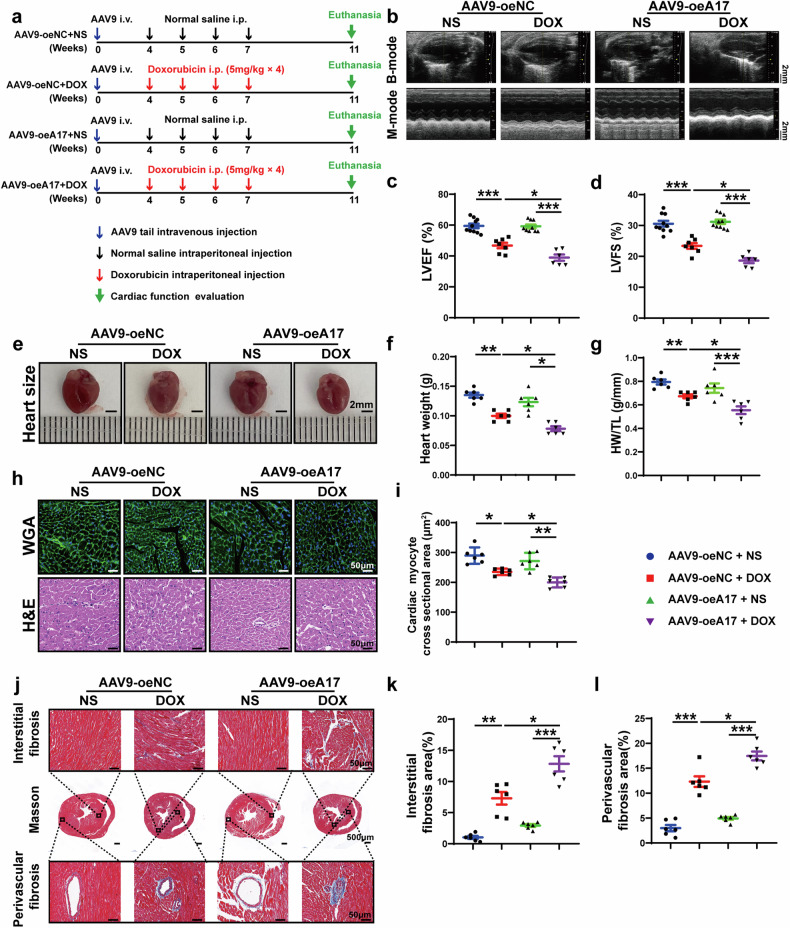
Echocardiographic measurements and histological staining in negative control and ADAM17-overexpressing mice treated with NS or DOX. **a** Experiment timeline in vivo. **b** Representative echocardiographic images (scale bar = 2 mm) showing B-mode and M-mode echocardiograms in four groups of mice. **c** Comparison of left ventricular ejection fraction (LVEF) among four groups of mice (*n* = 6–10 in each group). **d** Comparison of left ventricular fractional shortening (LVFS) among four groups of mice (*n* = 6–10 in each group). **e** Representative anatomical images of heart size (scale bar = 2 mm) in four groups of mice. **f** Comparison of heart weight among four groups of mice (*n* = 6 in each group). **g** Comparison of heart weight/tibial length (HW/TL) ratio among four groups of mice (*n* = 6 in each group). **h** Representative WGA and H&E staining of myocardial cross-sections (scale bar = 50 μm) in four groups of mice. **i** Comparison of cardiac myocyte cross-sectional area measured by WGA staining (*n* = 6 in each group). **j** Representative Masson’s trichrome staining of myocardial interstitial and perivascular fibrosis in four groups of mice. **k** Comparison of interstitial fibrosis area among four groups of mice (*n* = 6 in each group). **l** Comparison of perivascular fibrosis area among four groups of mice (*n* = 6 in each group). Values shown were mean and SEM. One-way ANOVA were applied in (**c**, **d**, **f**, **g**, **i**, **k** and **l**). *p < 0.05; **p < 0.01; ***p < 0.001

To further testify the causal relation between ADAM17 and doxorubicin-induced cardiomyopathy, wild-type C57BL/6J male mice were transfected with adeno-associated virus (AAV) 9 carrying ADAM17 (AAV9-oeA17) to overexpress ADAM17 and adeno-associated virus vehicle as negative control (AAV9-oeNC) by means of tail vein injection in the third proportion of in vivo experiments (Fig. 3a). Immunofluorescent staining showed that AAV9-mediated expression of ADAM17 was homogenously distributed in the heart (Supplementary Fig. 3a). Western blot and RT-PCR analysis confirmed that ADAM17 expression was upregulated in AAV9-oeA17 group versus the AAV9-oeNC group (Supplementary Fig. 3b–d). In addition, ADAM17 activity was significantly upregulated in the AAV9-oeA17 group compared with the AAV9-oeNC group (Supplementary Fig. 3e). Then, these mice were injected with doxorubicin or normal saline, and the results showed no significant difference in the survival rate between AAV9-oeNC mice and AAV9-oeA17 mice after doxorubicin injection (Supplementary Fig. 3f). Doxorubicin treatment further increased the ADAM17 expression in the heart of AAV9-oeA17 mice compared with normal saline (Supplementary Fig. 3g-h). However, LVEF and LVFS in the AAV9-oeA17 + DOX group were significantly reduced compared with those in the AAV9-oeNC + DOX group (Fig. 3b–d). In addition, ADAM17-overexpressing mice injected with doxorubicin had a lower heart weight and a smaller HW/TL ratio than AAV9-oeNC + DOX group mice (Fig. 3e–g). WGA staining demonstrated that myocardial cell cross-sectional area was decreased in AAV9-oeNC + DOX group relative to AAV9-oeNC + NS group, which was further decreased in AAV9-oeA17 + DOX group (Fig. 3h, i). Compared with AAV9-oeNC mice, myocardial interstitial fibrosis and perivascular fibrosis induced by doxorubicin were aggravated in ADAM17-overexpressing mice (Fig. 3j–l). These results suggested that ADAM17 overexpression in cardiomyocytes aggravated cardiac systolic dysfunction and fibrosis induced by doxorubicin.

### ADAM17 is involved in doxorubicin-mediated cardiomyocyte apoptosis in vivo

**Fig. 4:**
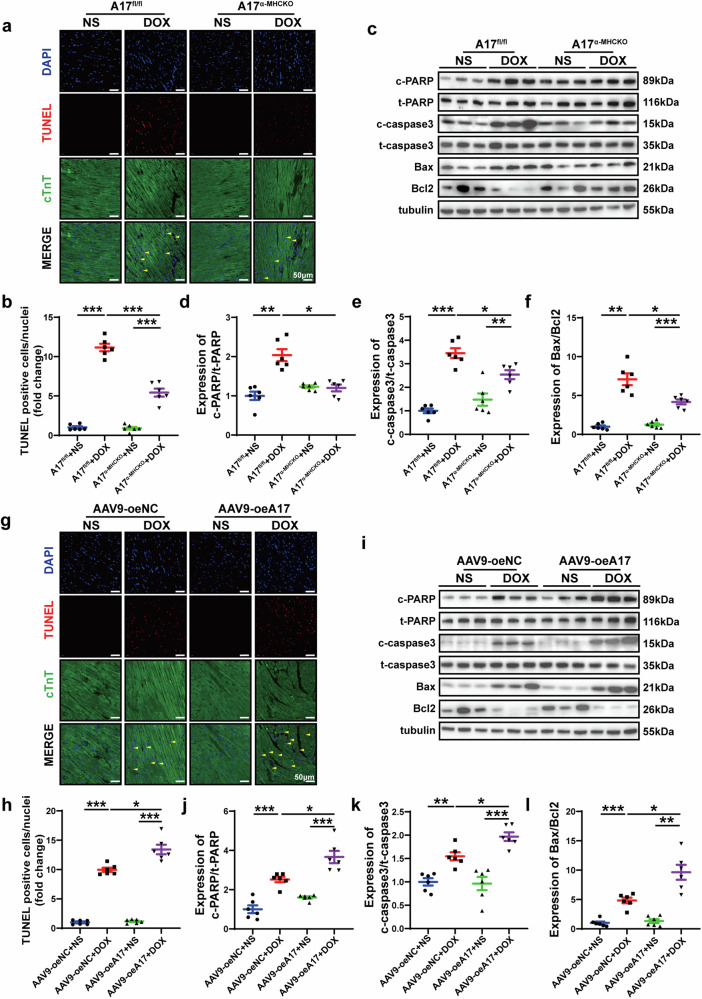
Effects of ADAM17 knockout and overexpression on cardiomyocyte apoptosis in four groups of mice treated with NS or DOX. **a** Representative TUNEL-positive cardiomyocyte staining in four groups of mice (scale bar = 50 μm). **b** Comparison of TUNEL-positive cardiomyocyte in the myocardium among four groups of mice (*n* = 6 in each group). **c** Representative western blot images of PARP, cleaved PARP, caspase 3, cleaved caspase 3, Bax and Bcl2 expression in the myocardium of four groups of mice. **d** Comparison of cleaved PARP/PARP expression among four groups of mice (*n* = 6 in each group). **e** Comparison of cleaved caspase3/caspase3 expression among four groups of mice (*n* = 6 in each group). **f** Comparison of Bax/Bcl2 expression among four groups of mice (*n* = 6 in each group). **g** Representative TUNEL-positive cardiomyocyte staining in four groups of mice (scale bar = 50 μm). **h** Comparison of TUNEL-positive cardiomyocyte in the myocardium among four groups of mice (*n* = 6 in each group). **i** Representative western blot images of PARP, cleaved PARP, caspase 3, cleaved caspase 3, Bax and Bcl2 expression in the myocardium of four groups of mice. **j** Comparison of cleaved PARP/PARP expression among four groups of mice (*n* = 6 in each group). **k** Comparison of cleaved caspase3/caspase3 expression among four groups of mice (*n* = 6 in each group). **l** Comparison of Bax/Bcl2 expression among four groups of mice (*n* = 6 in each group). Values shown were mean and SEM. One-way ANOVA were applied in (**b**, **d**–**f**, **h**, and **j**–**l**). *p < 0.05; **p < 0.01; ***p < 0.001

As apoptosis is a salient feature of doxorubicin-induced cardiomyopathy, we quantified the extent of apoptotic cardiomyocytes in heart tissue by TUNEL staining. Cardiomyocyte apoptosis level was similar in A17^fl/fl^ and A17^α-MHCKO^ mice. After doxorubicin injection, the number of TUNEL-positive cells was significantly higher in A17^fl/fl^ than in A17^α-MHCKO^ mice (Fig. 4a, b). Western blot results showed that compared with A17^fl/fl^ mice, the apoptosis-promoting protein expression of the cleaved PARP/PARP, cleaved caspase 3/caspase3 and Bax/Bcl2 in A17^α-MHCKO^ mice decreased (Fig. 4c–f). To explore the effect of ADAM17 overexpression on myocardial apoptosis induced by doxorubicin, we assessed the number of TUNEL-positive cells in the myocardium of mice after infusion of doxorubicin or normal saline. Cardiomyocyte apoptosis was similar in AAV9-oeA17 and AAV9-oeNC mice after normal saline treatment, while the percentage of TUNEL-positive cells in the AAV9-oeA17 + DOX group increased further than AAV9-oeNC + DOX group (Fig. 4g, h). In addition, western blot showed that expression of apoptosis-promoting proteins including cleaved PARP/PARP, cleaved caspase 3/caspase3, and Bax/Bcl2 in AAV9-oeA17 + DOX group increased compared with that in the AAV9-oeNC + DOX group (Fig. 4i–l). These results indicated that cardiomyocyte-specific knockout of ADAM17 significantly prevented myocardial cell apoptosis induced by doxorubicin, whereas ADAM17 overexpression in cardiomyocyte aggravated doxorubicin-induced cardiomyocyte apoptosis in vivo.

### ADAM17 is involved in doxorubicin-mediated cardiomyocyte apoptosis in vitro

To further explore the effect of doxorubicin on cardiomyocyte apoptosis, we stimulated NRCMs with doxorubicin at different concentrations and for various durations (Supplementary Fig. 4a). Similar to the results of doxorubicin stimulation at different concentrations, ADAM17 protein, mRNA levels and ADAM17 activity increased in a time-dependent manner (Supplementary Fig. 5a–d). In addition, western blot showed that the expression level of pro-apoptotic proteins including cleaved PARP/PARP, cleaved caspase 3/caspase3 and Bax/Bcl2 substantially increased with the increased doxorubicin concentration (Supplementary Fig. 5e–h) or treatment duration (Supplementary Fig. 5k–n). RT-PCR showed that the ratio of Bax/Bcl2 increased and CCK-8 assay conveyed that the cell viability decreased with the augmented doxorubicin concentration (Supplementary Fig. 5i, j) or the extension of treatment duration (Supplementary Fig. 5o, p). Then, we selected 1 μM doxorubicin treatment for 24 h as the protocol for subsequent experiment, because at this concentration and duration, cell apoptosis was obvious and cell viability was >50%.

**Fig. 5:**
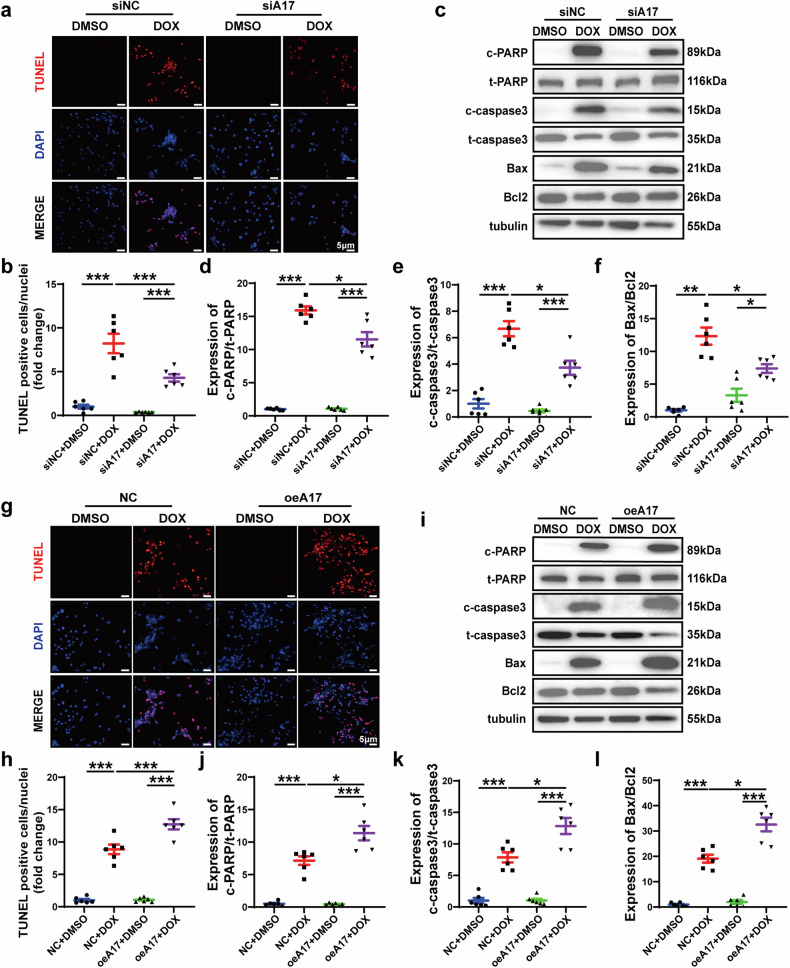
Effects of ADAM17 knockdown and overexpression on cardiomyocyte apoptosis in four groups of NRCMs treated with DMSO or DOX. **a** Representative TUNEL staining in four groups of NRCMs treated with siNC + DMSO, siNC + DOX, siA17 + DMSO and siA17 + DOX, respectively (scale bar = 5 μm). **b** Comparison of TUNEL-positive cells among four groups of NRCMs (*n* = 6 in each group). **c** Representative western blot images of PARP, cleaved PARP, caspase 3, cleaved caspase 3, Bax and Bcl2 expression in four groups of NRCMs. **d** Comparison of cleaved PARP/PARP expression among four groups of NRCMs (*n* = 6 in each group). **e** Comparison of cleaved caspase3/caspase3 expression among four groups of NRCMs (*n* = 6 in each group). **f** Comparison of Bax/Bcl2 expression among four groups of NRCMs (*n* = 6 in each group). **g** Representative TUNEL staining in four groups of NRCMs treated with NC + DMSO, NC + DOX, oeA17 + DMSO and oeA17 + DOX, respectively (scale bar = 5 μm). **h** Comparison of TUNEL-positive cells among four groups of NRCMs (*n* = 6 in each group). **i** Representative western blot images of PARP, cleaved PARP, caspase 3, cleaved caspase 3, Bax and Bcl2 expression in four groups of NRCMs. **j** Comparison of cleaved PARP/PARP expression among four groups of NRCMs (*n* = 6 in each group). **k** Comparison of cleaved caspase3/caspase3 expression among four groups of NRCMs (*n* = 6 in each group). **l** Comparison of Bax/Bcl2 expression among four groups of NRCMs (*n* = 6 in each group). Values shown were mean and SEM. One-way ANOVA were applied in (**b**, **d**–**f**, **h**, and **j–l**). *p < 0.05; **p < 0.01; ***p < 0.001

To further explore the potential role of ADAM17 in cardiomyocyte apoptosis induced by doxorubicin in vitro, we used siRNA to knockdown ADAM17 expression in NRCMs and treated NRCMs with doxorubicin in the second proportion of the in vitro experiment (Supplementary Fig. 4b). Western blot and RT-PCR showed that, compared with siNC group, ADAM17 protein and mRNA expression levels and ADAM17 activity in siA17 group were significantly decreased (Supplementary Fig. 6a–d). TUNEL staining showed that the number of TUNEL-positive cells in the siA17 + DOX group was less than that in the siNC + DOX group (Fig. 5a, b). In addition, western blot demonstrated that the expression level of apoptosis-promoting proteins including cleaved PARP/PARP, cleaved caspase 3/caspase3 and Bax/Bcl2 in the siA17 + DOX group were lower than that in the siNC + DOX group (Fig. 5c–f). Meanwhile, we overexpressed ADAM17 in NRCMs in vitro and carried out doxorubicin treatment in the third proportion of the in vitro experiment (Supplementary Fig. 4c). Western blot and RT-PCR showed that, compared with NC group, ADAM17 protein and mRNA expression levels and ADAM17 activity in oeA17 group were significantly increased (Supplementary Fig. 6e–h). TUNEL staining of NRCMs showed that the number of TUNEL-positive cells remarkably increased in the oeA17 + DOX group compared with the NC + DOX group (Fig. 5g, h). Western blot found that the expression level of apoptosis-promoting protein including cleaved PARP/PARP, cleaved caspase 3/caspasd3 and Bax/Bcl2 was higher in the oeA17 + DOX group than that in the NC + DOX group (Fig. 5i–l). These results demonstrated that ADAM17 deficiency inhibited while ADAM17 overexpression aggravated doxorubicin-mediated cardiomyocyte apoptosis in vitro.

### Upregulation of ADAM17 in doxorubicin-stimulated cardiomyocytes is mainly induced by C/EBPβ

To understand the mechanism of ADAM17 upregulation in doxorubicin-induced cardiomyopathy, bioinformatics tools were used to predict potential transcription factors in the ADAM17 promoter region, including the Animal Transcription factor database (Animal TFDB4), JASPAR, TRANSFAC, and PROMO. As shown in Supplementary Fig. 7a, we draw the Venn diagram based on cross-analysis of multiple datasets and found that CCAAT/enhancer binding protein beta (C/EBPβ) and Sp1 were the most promising transcription factors for binding to ADAM17 promoters. C/EBPβ is a transcription factor that binds the regulatory domains of promoters and/or enhancers of target genes involved in different biological processes.^26^ A previous study suggested that hypoxia instigated C/EBPβ signaling pathway, which initiated recruitment of C/EBPβ to the ADAM17 promoter and induced ADAM17 expression in human lung fibroblasts^27^ (Supplementary Fig. 7b). To explore the role of C/EBPβ in regulating ADAM17 gene transcription in doxorubicin-induced cardiotoxicity, we knocked down C/EBPβ expression in NRCMs using siRNA. Western blot and RT-PCR showed that C/EBPβ protein and mRNA expression levels were significantly decreased in siC/EBPβ group compared with siNC group (Supplementary Fig. 7c–e). In NRCMs, compared with the siNC + DMSO group, the protein and mRNA expression levels of both C/EBPβ and ADAM17 in the siNC + DOX group were significantly increased. However, the protein and mRNA expression levels of ADAM17 were dramatically decreased in the siC/EBPβ + DOX group versus the siNC + DOX group (Supplementary Fig. 7f–j), suggesting that C/EBPβ might be an upstream mediator of ADAM17 upregulation in mice with doxorubicin-induced cardiomyopathy.

### Differential gene expression in TNF signaling pathway of A17^fl/fl^ and A17^α-MHCKO^ mice treated with doxorubicin and normal saline

**Fig. 6:**
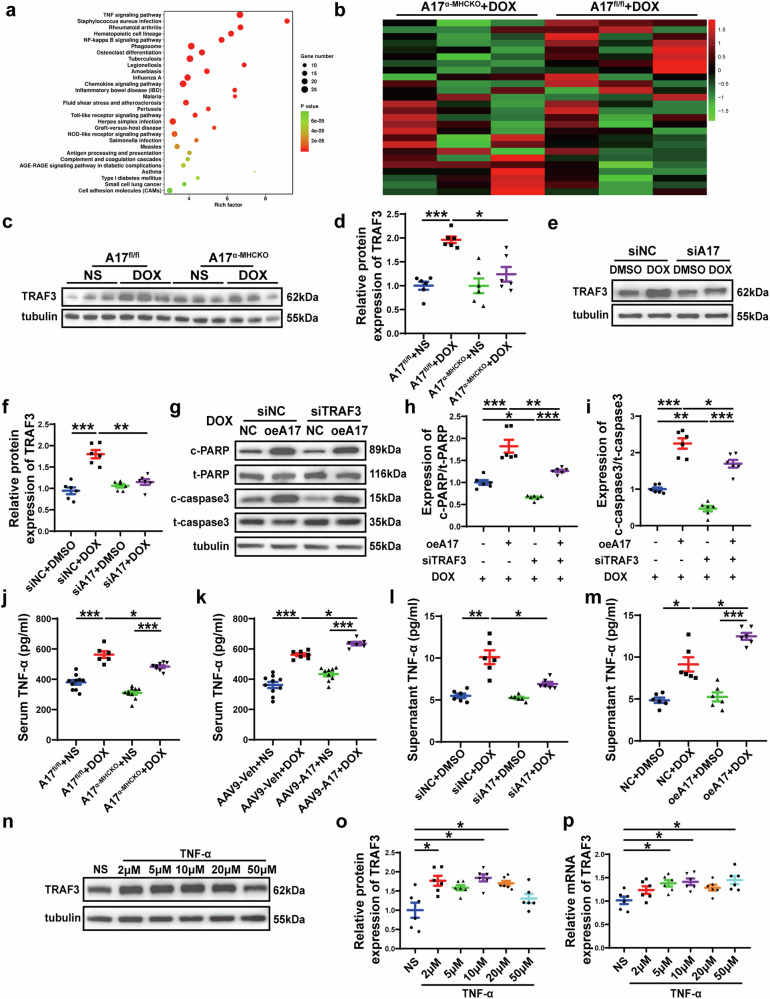
Transcriptome profiles of the myocardium in four groups of mice and the relation between ADAM17 and TRAF3 in the regulation of apoptosis-associated proteins in the NRCMs. **a** KEGG pathway enrichment analysis of differential expressing (DE) transcripts and bubble chart showing KEGG pathways enrichment in the heart of A17^fl/fl^ + NS and A17^fl/fl^ + DOX mice. **b** Heat map of 26 genes, which showed significant difference in the TNF signaling pathway between A17^fl/fl^ + NS and A17^fl/fl^ + DOX mouse hearts, were compared between A17^α-MHCKO^ + DOX and A17^fl/fl^ + DOX mouse hearts and the differences between the latter two groups were ranked by *p* values with the smallest *p* value on the top. **c** Representative western blot images of TRAF3 expression in the myocardium of four groups of mice. **d** Comparison of TRAF3 expression among four groups of mice (*n* = 6 in each group). **e**, **f** Representative western blot images and comparison of TRAF3 expression in four groups of NRCMs treated with siNC + DMSO, siNC + DOX, siA17 + DMSO and siA17 + DOX, respectively (*n* = 6 in each group). **g** Representative western blot images of PARP, cleaved PARP, caspase 3 and cleaved caspase 3 expression among four groups of NRCMs treated with NC + DOX, oe17 + DOX, siTRAF3 + DOX and oeA17 + siTRAF3 + DOX, respectively. **h** Comparison of cleaved PARP/PARP expression among four groups of NRCMs treated with NC + DOX, oe17 + DOX, siTRAF3 + DOX and oeA17 + siTRAF3 + DOX, respectively (*n* = 6 in each group). **i** Comparison of cleaved caspase3/caspase3 expression among four groups of NRCMs treated with NC + DOX, oe17 + DOX, siTRAF3 + DOX and oeA17 + siTRAF3 + DOX, respectively (*n* = 6 in each group). **j** Serum levels of TNF-α in A17^fl/fl^ and A17^α-MHCKO^ mice treated with NS or DOX (*n* = 6 in each group). **k** Serum levels of TNF-α in AAV9-oeNC and AAV9-oeA17 mice treated with NS or DOX (*n* = 6 in each group). **l** Supernatant levels of TNF-α in four groups of NRCMs treated with siNC + DMSO, siNC + DOX, siA17 + DMSO and siA17 + DOX, respectively (*n* = 6 in each group). **m** Supernatant levels of TNF-α in four groups of NRCMs treated with NC + DMSO, NC + DOX, oeA17 + DMSO and oeA17 + DOX, respectively (*n* = 6 in each group). **n**, **o** Representative western blot images and comparison of TRAF3 protein expression in the NRCMs treated with TNF-α at different concentrations for 24 h (*n* = 6 in each group). **p** Comparison of TRAF3 mRNA expression in NRCMs treated with TNF-α at different concentrations for 24 h (*n* = 6 in each group). Values shown were mean and SEM. One-way ANOVA were applied in (**d**, **f**, **h**–**m**, **o** and **p**). *p < 0.05; **p < 0.01; ***p < 0.001

Subsequently, RNA sequencing was performed in left ventricular samples from A17^fl/fl^ mice and A17^α-MHCKO^ mice treated with normal saline or doxorubicin. The differentially expressed genes of A17^fl/fl^ + DOX (*n* = 3) and A17^fl/fl^ + NS mice (*n* = 3) were analyzed by hierarchical clustering. Next, KEGG pathway analyses showed that the TNF pathway was significantly enriched in doxorubicin-treated mice (Fig. 6a). We identified 26 genes with changes in TNF signaling pathway. Then the expression of these 26 genes in A17^α-MHCKO^ + DOX group (*n* = 3) and A17^fl/fl^ + DOX group (*n* = 3) was analyzed by hierarchical clustering, and of these genes, TRAF3 expression showed the most significant difference between the two groups (Fig. 6b). These findings strongly suggested that TRAF3 may be a key molecule in the role of ADAM17 in doxorubicin-induced cardiotoxicity.

### ADAM17 aggravates cardiac injury via upregulating TRAF3 expression in doxorubicin-induced cardiomyopathy

To further investigate the role of TRAF3 in the effect of ADAM17 on doxorubicin-induced cardiotoxicity, we examined the protein expression of TRAF3 in the heart tissue of A17^fl/fl^ and A17^α-MHCKO^ mice after doxorubicin or normal saline treatment. Western blot showed that the protein expression of TRAF3 in A17^fl/fl^ mice after doxorubicin treatment was increased compared with A17^fl/fl^ mice after saline treatment, while the expression of TRAF3 in A17^α-MHCKO^ + DOX group was decreased compared with A17^fl/fl^ + DOX group (Fig. 6c, d). In NRCMs, the protein expression of TRAF3 was upregulated in the siNC + DOX group relative to the siNC + DMSO group, while TRAF3 expression in both siA17 + DMSO and siA17 + DOX was similar to the siNC + DMSO group (Fig. 6e, f). These results suggested that TRAF3 was a downstream molecule in the ADAM17 signaling.

Previous studies revealed that TRAF3 modulated apoptosis and inflammation-related signaling pathways in hepatic I/R injury to regulate cell survival.^18^ Immunofluorescence co-localization staining showed that TRAF3 expression was notably increased in cardiomyocytes in the DOX group compared to the NS group (Supplementary Fig. 8a, b). To further examine the role of TRAF3 in doxorubicin-induced cardiac injury by modulating cardiomyocyte apoptosis, AAV9 was used to knock down and overexpress (Supplementary Fig. 8c–h) TRAF3 in cardiomyocytes. As previously described, mice of AAV9-shNC group and AAV9-shTRAF3 group received either normal saline or doxorubicin injection (Supplementary Fig. 9a). After doxorubicin treatment, LVEF and LVFS in mice of AAV9-shTRAF3 group were markedly increased in comparison to those in AAV9-shNC mice (Supplementary Fig. 9b–d). In contrast, heart weight, HW/TL and cardiomyocyte cross-sectional area (Supplementary Fig. 9e–i) were increased in doxorubicin-injected AAV9-shTRAF3 mice compared with doxorubicin-injected AAV9-shNC mice. Masson staining showed that cardiac interstitial and perivascular fibrosis of doxorubicin-injected AAV9-shTRAF3 mice was also significantly improved compared with doxorubicin-injected AAV9-shNC mice (Supplementary Fig. 9j–l). After the injection of doxorubicin, the number of TUNEL-positive cells in AAV9-shTRAF3 mice was significantly reduced compared with AAV9-shNC mice (Supplementary Fig. 9m, n).

Both AAV9-oeNC and AAV9-oeTRAF3 mice received injection of doxorubicin or normal saline as described earlier (Supplementary Fig. 10a). Echocardiographic measurement of LVEF and LVFS in the AAV9-oeTRAF3 + DOX group were significantly lower than those in the AAV9-oeNC + DOX group (Supplementary Fig. 10b–d). Relative to AAV9-oeNC + DOX mice, heart weight, HW/TL, and cardiomyocyte cross-sectional area were significantly decreased in AAV9-oeTRAF3 + DOX mice (Supplementary Fig. 10e–i). In addition, myocardial interstitial and perivascular fibrosis was more prominent in doxorubicin-injected AAV9-oeTRAF3 mice than in doxorubicin-injected AAV9-oeNC mice (Supplementary Fig. 10j–l). The number of TUNEL-positive cells was significantly higher in AAV9-oeTRAF3 mice than in AAV9-oeNC mice after doxorubicin injection (Supplementary Fig. 10m, n). These results indicated that TRAF3 knockdown was mitigated while TRAF3 overexpression aggravated doxorubicin-induced cardiomyopathy via regulating cardiomyocyte apoptosis.

Thus, we speculated that ADAM17 may play a vital role in regulating apoptosis through TRAF3 in doxorubicin-induced myocardial injury and cardiac dysfunction. To verify this hypothesis, we used AAV9 to overexpress ADAM17 and knock down TRAF3 in the hearts of C57BL/6J mice, followed by doxorubicin injection (Supplementary Fig. 11a). After doxorubicin treatment, values of LVEF and LVFS were significantly higher in the AAV9-oeA17 + AAV9-shTRAF3 group than in the AAV9-oeA17 + AAV9-shNC group (Supplementary Fig. 11b–d). Similarly, doxorubicin-injected AAV9-oeA17 + AAV9-shTRAF3 mice showed improvements in heart weight, HW/TL and cardiomyocyte cross-sectional area in comparison with doxorubicin-injected AAV9-oeA17 + AAV9-shNC mice (Supplementary Fig. 11e–i). In addition, after doxorubicin treatment, AAV9-oeA17 + AAV9-shTRAF3 mice exhibited less extensive myocardial interstitial and perivascular fibrosis than AAV9-oeA17 + AAV9-shNC mice as depicted by Masson staining (Supplementary Fig. 11j–l). Furthermore, the number of TUNEL-positive cells in doxorubicin-injected AAV9-oeA17 + AAV9-shTRAF3 mice was significantly decreased versus that in doxorubicin-injected AAV9-oeA17 + AAV9-shNC mice (Supplementary Fig. 11m, n). Then we performed in vitro experiments to further validate these results (Supplementary Fig. 4d). We induced ADAM17 overexpression in NRCMs followed by TRAF3 knockdown and doxorubicin treatment. TRAF3 protein and mRNA expression levels were significantly decreased in siTRAF3 group compared with those in siNC group (Supplementary Fig. 12a–c). Western blot demonstrated that knockdown of TRAF3 markedly counteracted the increased expression of cleaved PARP and cleaved caspase3 caused by doxorubicin treatment and ADAM17 overexpression (Fig. 6g–i). Taken together, these results suggested that ADAM17 promoted doxorubicin-induced apoptosis primarily through TRAF3.

### ADAM17 upregulates TRAF3 expression through TNF-α

The mechanism of ADAM17 enhancing TRAF3 expression in doxorubicin-induced cardiomyopathy has not been clarified. In this study, KEGG showed that most differentially expressed genes were enriched into the TNF pathway, and ADAM17 is known as a TNF-α converting enzyme that converts TNF-α from the precursor form (pro TNF-α) to the mature form (sTNF-α) which is secreted into circulation.^12^ In addition, previous studies showed that inflammatory cytokines such as TNF-α are involved in the pathogenesis of doxorubicin-induced cardiotoxicity.^7,9^ Thus, we studied the effect of doxorubicin on TNF-α, and observed that the serum TNF-α levels were elevated in doxorubicin-treated mice compared to those treated with saline, which was consistent with the results of Nozaki et al.^28^ In contrast, cardiomyocyte ADAM17 knockout lowered the serum level of TNF-α enhanced by doxorubicin treatment whereas cardiomyocyte ADAM17 overexpression further raised the serum level of TNF-α already increased by doxorubicin treatment (Fig. 6j, k). In addition, TNF-α secretion from NRCMs treated by doxorubicin in vitro was increased, consistent with the results in vivo (Fig. 6l, m). As TNF-α is cardiotoxic and can induce cardiac dysfunction,^7^ and production of TNF-α by transgenic cardiomyocytes was sufficient to cause severe heart disease,^29^ we hypothesized that ADAM17 may upregulate TRAF3 expression through TNF-α. Therefore, we stimulated NRCMs with different concentrations of TNF-α for 24 h (Supplementary Fig. 13a) and found that the protein and mRNA expression levels of TRAF3 were indeed increased (Fig. 6n–p), thus confirming that our hypothesis was correct.

### TNF-α enhances TRAF3 expression through TNFR1

**Fig. 7:**
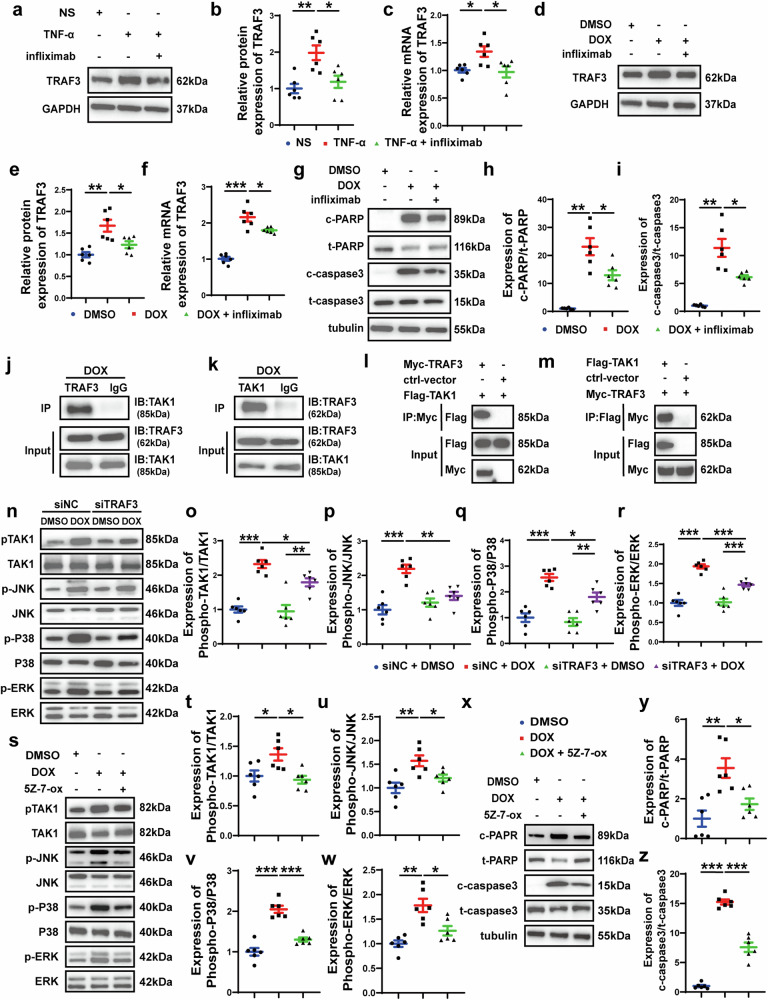
TNF-α enhanced TRAF3 expression in NRCMs treated with DOX, and interaction of TRAF3 and TAK1 leads to activation of MAPKs pathway. **a**, **b** Representative western blot images and comparison of protein expression of TRAF3 among three groups of NRCMs treated with NS, TNF-α and TNF-α + infliximab, respectively (*n* = 6 in each group). **c** Comparison of mRNA expression of TRAF3 among three groups of NRCMs treated with NS, TNF-α and TNF-α + infliximab, respectively (*n* = 6 in each group). **d**, **e** Representative western blot images and comparison of protein expression of TRAF3 among three groups of NRCMs treated with DMSO, DOX and DOX + infliximab, respectively (*n* = 6 in each group). **f** Comparison of mRNA expression of TRAF3 among three groups of NRCMs treated with DMSO, DOX, DOX + infliximab, respectively (*n* = 6 in each group). **g** Representative western blot images of cleaved PARP, PARP, cleaved caspase 3 and caspase 3 expression among three groups of NRCMs treated with DMSO, DOX and DOX + infliximab, respectively. **h** Comparison of cleaved PARP/PARP expression among three groups of NRCMs treated with DMSO, DOX and DOX + infliximab, respectively (*n* = 6 in each group). **i** Comparison of cleaved caspase3/caspase3 expression among three groups of NRCMs treated with DMSO, DOX, DOX + infliximab, respectively (*n* = 6 in each group). **j**, **k** Co-IP assay in NRCMs showing endogenous TRAF3 binding to TAK1. **l**, **m** Co-IP assay in HEK293T cells co-transfected with Myc-TRAF3 and Flag-TAK1 showing exogenous TRAF3 binding to TAK1. **n** Representative western blot images of phosphorylated TAK1, TAK1, phosphorylated JNK, JNK, phosphorylated P38 MAPK, P38 MAPK, phosphorylated ERK and ERK among four groups of NRCMs treated with siNC + DMSO, siNC + DOX, siTRAF3 + DMSO and siTRAF3 + DOX, respectively. **o** Comparison of protein expression of phosphorylated TAK1/TAK1 among four groups of NRCMs treated with siNC + DMSO, siNC + DOX, siTRAF3 + DMSO and siTRAF3 + DOX, respectively (*n* = 6 in each group). **p** Comparison of protein expression of phosphorylated JNK/JNK among four groups of NRCMs treated with siNC + DMSO, siNC + DOX, siTRAF3 + DMSO and siTRAF3 + DOX, respectively (*n* = 6 in each group). **q** Comparison of protein expression of phosphorylated P38 MAPK/P38 MAPK among four groups of NRCMs treated with siNC + DMSO, siNC + DOX, siTRAF3 + DMSO and siTRAF3 + DOX, respectively (*n* = 6 in each group). **r** Comparison of protein expression of phosphorylated ERK/ERK among four groups of NRCMs treated with siNC + DMSO, siNC + DOX, siTRAF3 + DMSO and siTRAF3 + DOX, respectively (*n* = 6 in each group). **s** Representative western blot images of phosphorylated TAK1, TAK1, phosphorylated JNK, JNK, phosphorylated P38 MAPK, P38 MAPK, phosphorylated ERK and ERK among three groups of NRCMs treated with DMSO, DOX and DOX + 5Z-7-ox, respectively. **t** Comparison of protein expression of phosphorylated TAK1/TAK1 among three groups of NRCMs treated with DMSO, DOX and DOX + 5Z-7-ox, respectively (*n* = 6 in each group). **u** Comparison of protein expression of phosphorylated JNK/JNK among three groups of NRCMs treated with DMSO, DOX and DOX + 5Z-7-ox, respectively (*n* = 6 in each group). **v** Comparison of protein expression of phosphorylated P38 MAPK/P38 MAPK among three groups of NRCMs treated with DMSO, DOX and DOX + 5Z-7-ox, respectively (*n* = 6 in each group). **w** Comparison of protein expression of phosphorylated ERK/ERK among three groups of NRCMs treated with DMSO, DOX and DOX + 5Z-7-ox, respectively (*n* = 6 in each group). **x** Representative western blot images of cleaved PARP, PARP, cleaved caspase 3 and caspase 3 expression among three groups of NRCMs treated with DMSO, DOX and DOX + 5Z-7-ox, respectively. **y** Comparison of cleaved PARP/PARP expression among three groups of NRCMs treated with DMSO, DOX and DOX + 5Z-7-ox, respectively (*n* = 6 in each group). **z** Comparison of cleaved caspase3/caspase3 expression among three groups of NRCMs treated with DMSO, DOX and DOX + 5Z-7-ox, respectively (*n* = 6 in each group). Values shown were mean and SEM. One-way ANOVA were applied in (**b**, **c**, **e**, **f**, **h**, **i**, **o**–**r**, **t**–**w**, **y** and **z**). *p < 0.05; **p < 0.01; ***p < 0.001

To further unveil the mechanism of TNF-α increasing TRAF3 expression, we used infliximab, a TNF-α monoclonal antibody to block TNF-α from binding to TNFR (Supplementary Fig. 13b). As shown in Fig. 7a–c, after treatment of NRCMs with infliximab, TNF-α-enhanced expression of TRAF3 was significantly abolished. Since TNFR1 and TNFR2 are the most common receptors of TNF-α,^30^ it is necessary to explore which ligand TNF-α interacts with to increase TRAF3 expression. Thus, we used different siRNA to knock down the expression of TNFR1 (Supplementary Fig. 14a–c) and TNFR2 (Supplementary Fig. 14d–f) in NRCMs respectively, and we found that TNFR1 knockdown in NRCMs significantly attenuated the increase of TRAF3 expression induced by TNF-α (Supplementary Fig. 14g–i). On the contrary, TNFR2 knockdown further enhanced TRAF3 expression already increased by TNF-α (Supplementary Fig. 14j–l). We further went on to knock down both TNFR1 and TNFR2 simultaneously in NRCMs, and found that TNF-α-enhanced TRAF3 expression was reduced (Supplementary Fig. 14m–o). These results suggested that the modulative effect of TNF-α on TRAF3 expression is mainly mediated by TNFR1 in the myocardium. Finally, we showed that infliximab treatment also attenuated the upregulation of TRAF3 (Fig. 7d–f), and decreased relative expression of cleaved PARP/PARP and cleaved caspase3/caspase3, in NRCMs induced by doxorubicin treatment (Fig. 7g–i). Subsequently, we investigated the effect of infliximab on doxorubicin-induced cardiac injury in C57BL/6J mice (Supplementary Fig. 15a). Echocardiographic results showed that LVEF and LVFS of mice in DOX + infliximab group were significantly increased compared with those in DOX group, and the levels of myocardial interstitial and perivascular fibrosis were significantly decreased compared with those in DOX group (Supplementary Fig. 15b–g). In addition, we found that infliximab attenuated the upregulation of TRAF3 in doxorubicin-injected mice and reduced the relative expression of cleaved PARP/PARP and cleaved caspase3/caspase3 (Supplementary Fig. 15h–l). These results suggested that doxorubicin treatment resulted in increased TNF-α secretion from cardiomyocytes, which upregulated TRAF3 expression in cardiomyocytes through TNFR1.

### TRAF3 stimulates TAK1 phosphorylation and activates downstream MAPKs pathway

Previous studies have shown that hepatocyte TRAF3 binds to TAK1 to induce TAK1 ubiquitination and subsequent autophosphorylation.^25^ Furthermore, TAK1 is a member of the MAP3K family and serves as a crucial regulator of MAP kinases and NF-κB signaling pathways.^31^ Our endogenous Co-IP experiments confirmed the interaction between endogenous TRAF3 and TAK1 in NRCMs after doxorubicin treatment (Fig. 7j, k), and confocal microscopy revealed that TRAF3 colocalized with TAK1, which was enhanced by doxorubicin treatment (Supplementary Fig. 16a, b). Subsequently, we transfected HEK293T cells with Myc-TRAF3 and Flag-TAK1 overexpressing plasmids, and performed exogenous Co-IP experiments, which confirmed the interaction between TRAF3 and TAK1 (Fig. 7l, m). Previous studies confirmed that TRAF3 activated downstream MAPKs pathway through TAK1 to promote hepatocyte apoptosis and neuronal apoptosis,^18,19^ and thus we speculated that TRAF3 may also promote cardiomyocyte apoptosis in doxorubicin-induced cardiotoxicity by stimulating TAK1 phosphorylation and activating MAPKs pathway. Our results showed that doxorubicin treatment induced activation of TAK1, JNK, P38 MAPK and ERK, which was attenuated after TRAF3 knockdown in NRCMs (Fig. 7n–r). Next, we explored the role of TAK1 in MAPKs activation induced by doxorubicin treatment. After treatment of NRCMs with TAK1 inhibitor 5Z-7-oxozeaenol (5Z-7-ox), doxorubicin-enhanced phosphorylation of TAK1, JNK, P38 MAPK and ERK was significantly inhibited (Fig. 7s–w). In addition, 5Z-7-ox treatment significantly alleviated doxorubicin-enhanced expression of cleaved PARP/PARP and cleaved caspase3/caspase3 (Fig. 7x–z). After establishing the importance of TAK1 in doxorubicin-induced cardiomyocyte apoptosis, we investigated whether 5Z-7-ox could prevent doxorubicin-induced cardiotoxicity by pharmacologically inhibiting TAK1 activation in mice (Supplementary Fig. 17a). Compared with the mice in DOX group, 5Z-7-ox increased LVEF and LVFS, and reduced myocardial interstitial and perivascular fibrosis (Supplementary Fig. 17b–g). In addition, 5Z-7-ox decreased cardiac TAK1, JNK, P38 MAPK and ERK phosphorylation levels activated by doxorubicin, and attenuated expression of pro-apoptotic molecules, cleaved PARP/PARP and cleaved caspase3/caspase3 (Supplementary Fig. 17h–o). These results proved that TRAF3 played an important role in doxorubicin-induced cardiomyocyte apoptosis via regulating TAK1 phosphorylation and MAPKs pathway.

### ADAM17 is involved in doxorubicin-induced cardiomyopathy via activation of TAK1 and JNK/P38 MAPK/ERK signaling in vivo and in vitro

Finally, we verified that ADAM17 was involved in doxorubicin-induced cardiotoxicity by affecting the activation of TAK1, JNK, P38 MAPK and ERK signaling pathways. We first demonstrated that TAK1, JNK, P38 MAPK and ERK signaling were activated after doxorubicin treatment in mice. However, A17^α-MHCKO^ mice treated with doxorubicin showed an attenuated activation of TAK1, JNK, P38 MAPK and ERK in myocardium compared to A17^fl/fl^ mice treated with doxorubicin (Supplementary Fig. 18a–e). Similarly, we found increased expression of phosphorylated protein of TAK1, JNK, P38 MAPK and ERK in NRCMs treated with doxorubicin, while these protein expression levels were downregulated in NRCMs treated with doxorubicin after ADAM17 knockdown (Supplementary Fig. 18f–j). In contrast, compared with the AAV9-oeNC + DOX group, the expression of phosphorylated protein of TAK1, JNK, P38 MAPK and ERK was further increased in the AAV9-oeA17 + DOX group (Supplementary Fig. 19a–e), suggesting that ADAM17 promoted cardiomyocyte apoptosis by activating TAK1, JNK, P38 MAPK and ERK signaling pathways. Furthermore, we showed that after ADAM17 overexpression, TAK1, JNK, P38 MAPK and ERK signaling in oeA17 + DOX group was further activated compared with NC + DOX group (Supplementary Fig. 19f–j). These results suggested that ADAM17 deficiency abolished TAK1 and MAPKs pathway activation induced by doxorubicin, while ADAM17 overexpression promoted cardiomyocyte apoptosis in doxorubicin-induced cardiomyopathy by activating TAK1, JNK, P38 MAPK and ERK pathway in vivo and in vitro.

### ADAM17 deficiency exerts cardioprotective effects against doxorubicin-induced cardiomyopathy without affecting the anti-tumor efficiency of doxorubicin

**Fig. 8:**
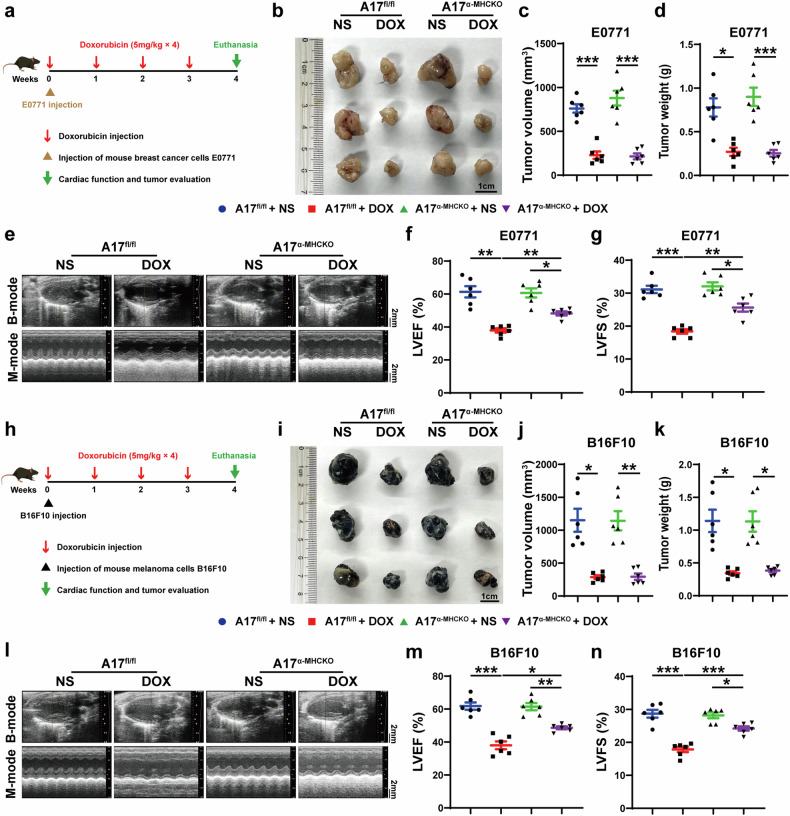
Echocardiographic measurements in A17^fl/fl^ and A17^α-MHCKO^ tumor-bearing mice treated with NS or DOX. **a** Experiment timeline of breast cancer-bearing mice. **b** Representative anatomical images of tumor size (scale bar = 1 cm) in four groups of mice. **c** Comparison of tumor volume among four groups of mice (*n* = 6 in each group). **d** Comparison of tumor weight among four groups of mice (*n* = 6 in each group). **e** Representative echocardiographic images (scale bar = 2 mm) showing B-mode and M-mode echocardiograms in four groups of mice. **f** Comparison of left ventricular ejection fraction (LVEF) among four groups of mice (*n* = 6 in each group). **g** Comparison of left ventricular fractional shortening (LVFS) among four groups of mice (*n* = 6 in each group). **h** Experiment timeline of melanoma-bearing mice. **i** Representative anatomical images of tumor size (scale bar = 1 cm) in four groups of mice. **j** Comparison of tumor volume among four groups of mice (*n* = 6 in each group). **k** Comparison of tumor weight among four groups of mice (*n* = 6 in each group). **l** Representative echocardiographic images (scale bar = 2 mm) showing B-mode and M-mode echocardiograms in four groups of mice. **m** Comparison of left ventricular ejection fraction (LVEF) among four groups of mice (*n* = 6 in each group). **n** Comparison of left ventricular fractional shortening (LVFS) among four groups of mice (*n* = 6 in each group). Values shown were mean and SEM. One-way ANOVA were applied in (**c**, **d**, **f**, **g**, **j**, **k**, **m** and **n**). *p < 0.05; **p < 0.01; ***p < 0.001

To demonstrate that ADAM17 deficiency in cardiomyocytes does not affect the efficacy of doxorubicin in suppressing tumor growth, we used breast cancer cells E0771 to establish tumor models in A17^fl/fl^ and A17^α-MHCKO^ female mice, who were treated with normal saline or doxorubicin once a week for 4 weeks after E0771 injection (Fig. 8a). Cardiac function and tumor volume and weight were evaluated 1 week after the last doxorubicin injection. In both A17^fl/fl^ and A17^α-MHCKO^ mice, doxorubicin treatment reduced breast tumor volume and weight and lowered LVEF and LVFS compared with normal saline treatment, but doxorubicin treatment did not induce significant difference in tumor size between A17^fl/fl^ and A17^α-MHCKO^ tumor-bearing mice (Fig. 8b–d). Echocardiographic results showed that values of LVEF and LVFS in A17^α-MHCKO^ tumor-bearing mice were significantly higher than those in A17^fl/fl^ tumor-bearing mice (Fig. 8e–g). To rule out the sex difference in anti-tumor effect of doxorubicin treatment, we used melanocytes B16F10 to establish melanoma models in male A17^fl/fl^ and A17^α-MHCKO^ mice, who were treated with saline or doxorubicin once a week for 4 weeks after B16F10 injection (Fig. 8h). Similarly, cardiac function and tumor volume and weight were evaluated 1 week after the last doxorubicin injection. In accordance with the results of breast cancer-bearing mice, relative to the normal saline group, doxorubicin treatment reduced the volume and weight of melanoma and lowered LVEF and LVFS. However, there was no significant difference in tumor size between A17^fl/fl^ and A17^α-MHCKO^ tumor-bearing mice treated with doxorubicin (Fig. 8i–k). Echocardiographic results showed that values of LVEF and LVFS in A17^α-MHCKO^ tumor-bearing mice were significantly higher than those in A17^fl/fl^ tumor-bearing mice (Fig. 8l–n). These results indicated that cardiomyocyte ADAM17 deficiency exerts a cardioprotective effect on doxorubicin-induced cardiomyopathy without affecting the anti-tumor effect of doxorubicin.

## Discussion

The current study revealed several significant findings. First, doxorubicin treatment increased the expression and activity of ADAM17 in the mouse heart, which was probably mediated by C/EBPβ. Second, cardiomyocyte-specific ADAM17 knockout ameliorated doxorubicin-induced cardiac dysfunction, cardiac fibrosis and cardiomyocyte atrophy and apoptosis without compromising the anti-tumor efficacy of doxorubicin, whereas cardiomyocyte-specific ADAM17 overexpression aggravated these pathological changes in mice. Third, doxorubicin treatment in NRCMs enhanced the expression and activity of ADAM17. ADAM17 knockdown in NRCMs significantly inhibited while ADAM17 overexpression in NRCMs aggravated doxorubicin-induced cell apoptosis. Fourth, ADAM17 promoted TNF-α maturation, which upregulated the expression of TRAF3 through TNFR1. Finally, increased TRAF3 bound to TAK1 and induced TAK1 autophosphorylation, leading to activated JNK/P38 MAPK/ERK pathway. To our knowledge, our study is the first to demonstrate that ADAM17 plays a key role in the pathogenesis of doxorubicin-induced cardiomyopathy and offers a potential therapeutic target for this intractable disorder.

There were few studies in the literature on the role of ADAM17 in the pathogenesis of cardiomyopathy. Early studies found that ADAM17 expression was significantly elevated in the myocardial tissue of patients with dilated cardiomyopathy and hypertrophic obstructive cardiomyopathy,^32^ and ADAM17 knockdown attenuated cardiac hypertrophy and fibrosis in spontaneous hypertensive rats and angiotensin II-infused mice.^33^ In contrast, an early study showed that cardiomyocyte ADAM17 knockout in a mouse model of acute myocardial infarction resulted in left ventricular dilatation and dysfunction by inhibiting VEGFR2 transcription and angiogenesis.^34^ On the contrary, a recent study in a similar mouse model reported that ADAM17 expression was increased in acute myocardial infarction, which blunted the cardioprotective effect of ACE2 by enhanced ACE2 shedding from cardiomyocytes, and administration of specific ADAM17 blocker TAPI-1 substantially ameliorated cardiac remodeling and dysfunction in mice with myocardial infarction.^35^ Our team demonstrated that ADAM17 expression substantially increased in diabetic cardiomyopathy, and ADAM17 deficiency effectively alleviated diabetic cardiomyopathy via upregulating AMPK pathway and improving autophagy process.^14^ However, the role of ADAM17 in chemotherapeutic drug-associated cardiomyopathy has not been explored. In the present study, we indisputably demonstrated that ADAM17 expression and activity were significantly increased in a mouse model of doxorubicin-induced cardiomyopathy. Consequently, cardiomyocyte-specific knockout of ADAM17 markedly attenuated cardiac dysfunction and fibrosis, whereas ADAM17 overexpression exerted an opposite effect. These results were consistent with our previous studies in diabetic cardiomyopathy.

However, the reason why ADAM17 expression and activity were elevated in doxorubicin-induced cardiomyopathy was unknown. Bioinformatics tools predicted that C/EBPβ may be a potential transcription factor that binds to the ADAM17 promoter. The expression of ADAM17 in human lung fibroblasts was upregulated due to hypoxia activation of C/EBPβ.^27^ Previous studies have shown that C/EBPβ is upregulated in a NF-κB-dependent manner during doxorubicin-induced senescence of cancer cells.^36^ The expression of C/EBPβ was significantly increased in doxorubicin-induced nephropathy rats and doxorubicin-treated HK2 cells.^37^ C/EBPβ is one of the key downstream molecules sensitive to AMPK, and in fact, several studies have highlighted that C/EBPβ is sensitive to AMPK phosphorylation.^38,39^ Doxorubicin, however, is a well-defined AMPK inhibitor that can damage mitochondria and cause β-oxidation dysfunction.^40^ Thus, it is likely that doxorubicin may upregulate C/EBPβ in doxorubicin-induced cardiomyopathy by inhibiting AMPK expression in cardiomyocytes.

ADAM17 is essential for fetal development and gene deletion of ADAM17 is embryonically lethal.^11^ Previous studies in our laboratory have shown that fibroblast-specific knockout ADAM17 in mice elicited a high inflammatory state, leading to sepsis. Thus, in this study, we constructed cardiomyocyte-specific ADAM17-knockout mice that developed normally after birth. In addition, we constructed cardiomyocyte-specific ADAM17-overexpressing mice with AAV9 targeting cardiomyocyte-specific cTnT promoter. Using these experimental tools, we have demonstrated that ADAM17 is a key regulator of cardiomyocyte apoptosis. Previous studies showed that ADAM17 regulated pathologic apoptosis in different diseases by controlling the activity of multiple signaling pathways. ADAM17 deletion reduced the apoptotic response of cardiomyocytes in diabetic cardiomyopathy by activating AMPK pathways and improving autophagy process.^14^ In the present study, we examined the expression of autophagy-related proteins of p62 and LC3 in A17^fl/fl^ + NS, A17^fl/fl^ + DOX, A17^α-MHCKO^ + NS and A17^α-MHCKO^ + DOX groups and found no difference in p62 and LC3 expression among the four groups of mice (Supplementary Fig. 20a–c). Thus, ADAM17 deficiency reduced cardiomyocyte apoptosis in doxorubicin-induced cardiomyopathy probably not by improving autophagy. ADAM17 deficiency in the ADAM17^ex/ex^ mice significantly ameliorated acinar cell injury in acute pancreatitis via IL-6 *trans*-signaling pathways.^41^ Genetic blockade of ADAM17 protected mice from cigarette smoking-induced pulmonary inflammation and alveolar cell apoptosis.^42^ Moreover, inhibition of ADAM17/TNF-α axis reduced downstream phosphorylation of P38 and P53, thereby abating chronic stress-induced apoptosis of hippocampal cells.^43^ To unveil the downstream targets of ADAM17, we performed myocardial RNA sequencing and found a number of genes enriched in TNF pathway in the cardiac tissues of doxorubicin-treated mice. In addition, TRAF3 expression was increased in the hearts of doxorubicin-treated A17^fl/fl^ mice, but decreased in those of doxorubicin-treated A17^α-MHCKO^ mice. These results suggested that TRAF3 may be a downstream target of ADAM17 in regulating cardiomyocyte apoptosis in doxorubicin-induced cardiomyopathy.

Like other TRAF members, TRAF3 has a TRAF domain, which includes an amino terminal curling helix region (TRAF-N) and a C-terminal domain (TRAF-C).^18^ The binding ability of the TRAF domain enables TRAF3 to have adaptor protein activity, which facilitates the formation of intracellular domain complexes across the membrane receptor and signaling to the downstream cascade.^16^ TRAF family members are involved in a variety of biological processes, such as immune response, differentiation, cell survival and proliferation,^44^ and among these members, TRAF3 is most elusive, possibly because of the lethality of TRAF3-KO mice at birth, its selective recruitment into different complexes, its different functional regulation of different processes, and its different specific functions in different cell types.^19^ Cardiomyocyte-specific TRAF3-knockdown mice showed significantly alleviated cardiac hypertrophy, fibrosis, and dysfunction 4 weeks after aortic banding.^17^ Our study showed that the expression of TRAF3 in the hearts of A17^α-MHCKO^ mice was lower than that of A17^fl/fl^ mice treated with doxorubicin. Similarly, ADAM17 knockdown significantly decreased doxorubicin-enhanced expression of TRAF3 in NRCMs.

In addition, we used AAV9 to knockdown and overexpress TRAF3 in cardiomyocytes, respectively, and found that TRAF3 deletion significantly attenuated cardiac dysfunction and fibrosis, whereas TRAF3 overexpression exhibited the opposite effect. Furthermore, TRAF3 knockdown blunted the promotive effect of ADAM17 overexpression on cardiomyocyte apoptosis after doxorubicin treatment in vivo and in vitro. These results demonstrated that ADAM17 affected doxorubicin-induced cardiomyopathy at least in part through TRAF3.

In the current study, RNA sequencing identified a number of genes which were highly enriched in the TNF pathway. As ADAM17 is a TNF-α converting enzyme that converts tmTNF-α to sTNF-α, we suspected that TNF-α may play a role in upregulation of TRAF3. Compared with the saline-treated mice, the serum level of TNF-α in doxorubicin-treated mice and the medium level of sTNF-α in doxorubicin-treated NRCMs were substantially increased. The protein and mRNA expression TRAF3 were increased in TNF-α concentration-dependent manner, and infliximab, a TNF-α monoclonal antibody, blunted TNF-α-induced increase in TRAF3 expression in NRCMs. Furthermore, we used siRNA to knockdown two TNF-α receptors, TNFR1 and TNFR2, to explore the downstream mechanism of the effect of TNF-α on TRAF3. Interestingly, only TNFR1 knockdown blocked the enhancing effect of TNF-α on TRAF3 expression, whereas TNFR2 knockdown exhibited an opposite effect, which may be related to TNFR2 inducing TRAF3 degradation.^45^ These results were consistent with previous studies suggesting that TNFR2-mediated signaling had a protective effect on the heart, while the deteriorative effect was mainly related to the activation of TNFR1.^46^ In addition, infliximab mitigated the expression of TRAF3 already enhanced by doxorubicin treatment in mice and NRCMs, and improved doxorubicin-induced cardiomyocyte apoptosis in vivo and in vitro. Thus, TNF-α upregulated TRAF3 in cardiomyocyte through TNFR1 which increased cardiomyocyte apoptosis, whereas TNFR1 deficiency blunted TNF-α-enhanced TRAF3 upregulation. Notably, our results strongly suggested that ADAM17 promoted doxorubicin-induced cardiomyocyte apoptosis primarily through upregulating TRAF3 by shedding membrane-bound TNF-α to generate its bioactive soluble form. In contrast, ADAM17 deficiency of cardiomyocyte improved cell apoptosis due to the blockade of ADAM17-dependent shedding of soluble and active TNF-α, and consequently, reduced expression of TRAF3.

TAK1 is a serine/threonine kinase regulating a wide range of physiological and pathological cellular processes.^23,47^ TAK1 can be activated by a variety of stimuli, including IL-1, TNF-α, TGF-β or toll-like receptor ligands, and primarily regulates cell death and inflammation signaling.^21,48^ A wealth of evidence indicates that TAK1 plays a key role in modulating cell apoptosis via activating downstream pathways such as P38 MAPK, JNK, ERK-1/2 and NF-κB p65.^31,49–51^ Previous studies have confirmed that C-terminal (TRAF) domain of TRAF3 binds to the C-terminal TAB2/3-binding domain (TAB2/3 BD) of TAK1, and then, the RING finger of TRAF3 ubiquitinates TAK1 leads to phosphorylation and activation of TAK1, thereby enhancing the activation of downstream IKK-β–NF-κB and MKK–JNK signaling cascades to regulate hepatic steatosis.^25^ In the present study, Co-IP assay and fluorescence staining co-localization demonstrated that cardiomyocyte TRAF3 bound to TAK1, which was enhanced by doxorubicin. In addition, TRAF3 knockdown significantly attenuated TAK1 phosphorylation and expression of downstream MAPKs pathway upregulated by doxorubicin treatment in NRCMs. Similarly, 5Z-7-ox, a pharmacological inhibitor of TAK1, significantly inhibited phosphorylation of TAK1 and expression of downstream MAPKs pathways upregulated by doxorubicin treatment in mouse hearts and NRCMs, and alleviated cardiomyocyte apoptosis induced by doxorubicin in vivo and in vitro. These results suggested that in doxorubicin-induced cardiomyopathy, upregulated TRAF3 may bind to TAK1 and promote its phosphorylation, thereby inducing downstream activation of JNK, P38 MAPK, ERK and eliciting doxorubicin cardiotoxicity.

It has been shown by previous studies that activated MAPKs may transmit extracellular signals to regulate cell growth, proliferation, differentiation, migration and apoptosis,^52^ and cell apoptosis can be induced by extracellular stimuli such as chemotherapy drugs. Increasing evidence supports the role of P38 MAPK as a tumor suppressor, and activation of P38 MAPK enhances the apoptotic response to chemotherapeutic drugs.^52^ JNK regulates cell apoptosis through two different mechanisms: first, it promotes phosphorylation of c-Jun and ATF-2, leading to activation of AP-1 and expression of proteins associated with the Fas/FasL signaling pathway.^53^ Second, JNK mediates phosphorylation of anti-apoptotic protein Bcl2/Bcl-xL to alter mitochondrial membrane potential, leading to the release of cytochrome C and activation of caspase 9 and caspase 3 to induce apoptosis.^54^ Thus, JNK is a positive regulator of apoptosis induced by genotoxic stress, and both P38 MAPK and JNK mediate apoptosis and autophagy in response to extracellular stimuli such as chemotherapy agents. In addition, different cellular injuries can induce apoptosis by activating the ERK signaling pathway. For instance, cisplatin-induced DNA damage can activate ERK1/2 and increase p53 protein levels, and ERK1/2 may interact with p53 and induce phosphorylation of p53 at Ser15. Thus, the upregulation of p53 by ERK signaling may be one of the mechanisms of cell apoptosis induced by DNA damage.^55^ Taken together, JNK, P38 MAPK and ERK can be activated individually or in combination during the process of apoptosis induced by many anticancer drugs such as vincristine and paclitaxel.^56^

Previous studies have shown that ADAM17 is upregulated in most tumor cells.^57^ Activated ADAM17 markedly reduced the impact of chemotherapy on tumor growth and cell apoptosis, thereby mediating chemotherapy resistance.^58^ Therefore, it is necessary to determine whether ADAM17 as a cardioprotective target interferes with the anti-tumor effects of doxorubicin. Doxorubicin is commonly used to treat patients with lymphomas, leukemia, breast cancer and other types of malignancies.^59^ Although doxorubicin is not commonly used for the treatment of melanoma patients, many experimental studies have applied doxorubicin to inhibit the growth of melanoma in mice.^60^ In this study, a female mouse model of breast cancer and doxorubicin-induced cardiomyopathy, and a male mouse model of melanoma and doxorubicin-induced cardiomyopathy were developed, which showed that cardiomyocyte-specific knockout of ADAM17 improved doxorubicin-induced cardiac dysfunction without affecting the anti-tumor effect of doxorubicin.

Our study contains several limitations. First, we did not perform experiments related to oxidative stress in doxorubicin-induced cardiomyopathy, as doxorubicin-induced apoptosis may be triggered by oxidative stress. However, RNA sequencing in our study did not reveal a primary role of oxidative stress-related genes and available antioxidant therapies have failed to improve doxorubicin-induced cardiomyopathy.^3^ Second, doxorubicin may induce various forms of programmed cell death apart from apoptosis, such as pyroptosis, necroptosis and ferroptosis.^61–63^ Thus, further research is needed to explore the impact of ADAM17 on other forms of cell death in doxorubicin-induced cardiomyopathy.

In conclusion, ADAM17 acted as a positive regulator of cardiomyocyte apoptosis and cardiac remodeling and dysfunction induced by doxorubicin by upregulating TRAF3/TAK1/MAPKs signaling. Mechanistically, doxorubicin enhanced the expression of transcription factor C/EBPβ, leading to increased expression and activity of ADAM17 in cardiomyocyte, which in turn enhanced the shedding of soluble and active TNF-α and upregulated the expression of TRAF3. Increased TRAF3 bound to TAK1 and promoted TAK1 autophosphorylation and activation, resulting in activated downstream MAPKs pathway and cardiomyocyte apoptosis in doxorubicin-induced cardiomyopathy (Schematic diagram). Thus, targeting ADAM17/TRAF3/TAK1/MAPKs signaling holds a promising potential for treating doxorubicin-induced cardiotoxicity.

## Materials and methods

### Generation of cardiomyocyte-specific ADAM17-knockout mice

ADAM17^flox/flox^ (A17^fl/fl^) mice with C57BL/6J background were obtained from the Jackson laboratory and α-myosin heavy chain (α-MHC)-Cre mice were derived from the Model Animal Research Center at Nanjing University. A17^fl/fl^ mice were crossed with α-MHC-Cre mice to obtain cardiomyocyte-specific ADAM17-knockout mice (A17^α-MHCKO^) through excising specifically exon 2 of the ADAM17 gene in cardiomyocytes. PCR fragments isolated from mouse tails and amplified from genomic DNA were used to confirm genotypes of A17^fl/fl^ mice and A17^α-MHCKO^ mice. The primer sequences and cycling conditions were listed in Supplementary Table 1. The knockout efficiency was assessed by measuring ADAM17 expression with western blot and RT-PCR.

### ADAM17 and TRAF3 overexpression by adeno-associated virus 9

To overexpress ADAM17 and TRAF3 in the cardiomyocyte of C57BL/6J mice, a cDNA encoding ADAM17 and TRAF3 sequence and cardiomyocyte-specific cTnT promoter were produced and inserted into AAV serotype 9 packaging vehicle, and AAV9 vehicle was applied as a negative control. AAV9-cTnTp-3Flag-ADAM17 (AAV9-oeA17), AAV9-cTnTp-3Flag-NC (AAV9-oeNC), AAV9-cTnTp-mcherry-TRAF3 (AAV9-oeTRAF3) and AAV9-cTnTp-mcherry-NC (AAV9-oeNC) were purchased from GeneChem Co., Ltd. (Shanghai, China) and injected to 8-week-old male mice through a tail vein with a dose of 200 μL virus at a titer of 5 × 10^11^ v.g./mL per mouse. The efficiency of virus transfection was measured by the expression of ADAM17 and TRAF3 by western blot and RT-PCR.

### TRAF3 knockdown by adeno-associated virus 9

To knockdown cardiomyocyte TRAF3 in C57BL/6J mice and ADAM17 overexpression mice, a cDNA encoding TRAF3 sequence and cardiomyocyte-specific cTnT promoter was produced and inserted into AAV9 packaging vehicle, and AAV9 vehicle was applied as a negative control. AAV9-cTnTp-mcherry-TRAF3-shRNA (AAV9-shTRAF3) and AAV9-cTnTp-mcherry-NC-shRNA (AAV9-shNC) were purchased from GeneChem Co., Ltd. (Shanghai, China) and injected to C57BL/6J mice and ADAM17 overexpression mice as previously described. The efficiency of virus transfection was measured by the expression of TRAF3 by western blot and RT-PCR.

### Animal model and grouping

Animal experiments consisted of eight proportions. In the first proportion of the in vivo experiments (Fig. 1a), male C57BL/6J mice aged 8 weeks were randomly divided into two groups (*n* = 10/group): normal saline (NS) group and doxorubicin (DOX) group. The mice in the DOX group were injected with an accumulative dose of 20 mg/kg doxorubicin (MCE, USA, cat# HY-15142) [5 mg/kg intraperitoneal (i.p.) injection at days 0, 7, 14 and 21], whereas an equivalent volume of normal saline was administered by i.p. injection to the NS group.^64^ Although doxorubicin is generally delivered through intravenous infusion in clinical setting, i.p. injection is widely used in mouse models of doxorubicin-induced cardiomyopathy for the advantages of stable serum drug concentration, high success rate and easy reproducibility.^65^ These mice were observed daily and finally euthanized with an overdose of sodium pentobarbital (200 mg/kg, i.p.) 4 weeks after the final doxorubicin or saline injection.

In the second proportion of the in vivo experiments (Fig. 2a), A17^α-MHCKO^ mice and their littermates A17^fl/fl^ mice were collected, who were randomly divided into four groups (*n* = 10 in each group): A17^fl/fl^ + NS, A17^fl/fl^ + DOX, A17^α-MHCKO^ + NS, A17^α-MHCKO^ + DOX. The injection method and doses of doxorubicin and normal saline were the same as those described earlier, and these mice were euthanized 4 weeks after the last doxorubicin or saline injection.

In the third proportion of the in vivo experiments (Fig. 3a), C57BL/6J male mice aged 8 weeks were selected and injected with ADAM17-overexpressing AAV9 and corresponding virus vehicle through the tail vein as previously described. The mice were randomly divided into four groups (*n* = 10 per group): AAV9-oeNC + NS, AAV9-oeNC + DOX, AAV9-oeA17 + NS and AAV9-oeA17 + DOX. The injection method and doses of normal saline and doxorubicin were the same as those described earlier. Four weeks after virus injection, the mice received i.p. injection of doxorubicin or equal volume of normal saline, as described earlier, and were euthanized 4 weeks after the last doxorubicin and saline injection.

In the fourth proportion of the in vivo experiments (Supplementary Fig. 9a), C57BL/6J male mice aged 8 weeks were collected and injected with TRAF3-knockdown AAV9 and corresponding virus vehicle through the tail vein as previously described. The mice were randomly divided into four groups (*n* = 10/group): AAV9-shNC + NS, AAV9-shNC + DOX, AAV9-shTRAF3 + NS and AAV9-shTRAF3 + DOX. The injection method and doses of normal saline and doxorubicin were the same as those described earlier. Four weeks after virus injection, the mice received i.p. injection of doxorubicin or equal volume of normal saline, as described earlier, and were euthanized 4 weeks after the last doxorubicin and saline injection.

In the fifth proportion of the in vivo experiments (Supplementary Fig. 10a), C57BL/6J male mice aged 8 weeks were applied and injected with TRAF3-overexpressing AAV9 and corresponding virus vehicle through the tail vein as previously described. The mice were randomly divided into four groups (*n* = 10/group): AAV9-oeNC + NS, AAV9-oeNC + DOX, AAV9-oeTRAF3 + NS and AAV9-oeTRAF3 + DOX. The injection method and doses of normal saline and doxorubicin were the same as those described earlier. Four weeks after virus injection, the mice received i.p. injection of doxorubicin or equal volume of normal saline, as described earlier, and were euthanized 4 weeks after the last doxorubicin and saline injection.

In the sixth proportion of the in vivo experiments (Supplementary Fig. 11a), the AAV9-oeNC and AAV9-oeA17 mice were injected with TRAF3-knockdown AAV9 and corresponding virus vehicle through the tail vein as previously described. The mice were randomly divided into four groups (*n* = 10/group): AAV9-oeNC + AAV9-shNC + DOX, AAV9-oeNC + AAV9-shTRAF3 + DOX, AAV9-oeA17 + AAV9-shNC + DOX, and AAV9-oeA17 + AAV9-shTRAF3 + DOX. The injection method and dose of doxorubicin were the same as those described earlier. Four weeks after virus injection, the mice received i.p. injection of doxorubicin as described earlier, and were euthanized 4 weeks after the last doxorubicin injection.

In the seventh proportion of the in vivo experiments (Supplementary Fig. 15a), C57BL/6J male mice at the age of 8 weeks were selected and randomly divided into three groups (*n* = 10/group): NS, DOX, DOX + infliximab, who received i.p. injection of normal saline, doxorubicin and infliximab, a monoclonal antibody of TNF-α,^66^ respectively. The injection method and doses of normal saline and doxorubicin were the same as those described earlier. Infliximab was injected after the last dose of doxorubicin, and 4 weeks later these mice were euthanized.

In the eighth proportion of the in vivo experiments (Supplementary Fig. 17a), 8-week-old C57BL/6J male mice were used and randomly divided into three groups (*n* = 10/group): NS, DOX, DOX + 5Z-7-ox, who received i.p. injection of normal saline, doxorubicin and 5Z-7-ox, an inhibitor of TAK1,^67,68^ respectively. The injection method and doses of normal saline and doxorubicin were the same as those described earlier. 5Z-7-ox was injected after the last dose of doxorubicin and 4 weeks later these mice were euthanized.

Different mouse groups were assigned in a randomized manner and investigators were blinded to the allocation of different groups when conducting drug treatments and outcome evaluations. Based on our preliminary experiments, we assume that type I error (α) and type II error (β) are 5% and 0.20%, respectively, with a power >0.80, and the sample size required is 6. By taking into account of accidental mouse death, we set *n* = 10 in each mouse group of this study. Exact animal numbers are shown in respective figure legends.

### Mouse tumor models and tumor studies

To examine doxorubicin-induced cardiotoxicity in the tumor-bearing mice, mouse breast cancer cells E0771 were subcutaneously implanted into the mammary fat pads of 8-week-old female A17^fl/fl^ and A17^α-MHCKO^ mice, respectively (Fig. 8a). These breast cancer-bearing mice were randomly divided into four groups (*n* = 10/group): A17^fl/fl^ + NS, A17^fl/fl^ + DOX, A17^α-MHCKO^ + NS, A17^α-MHCKO^ + DOX. To avoid the effects of gender differences, mouse melanoma cells B16F10 were subcutaneously injected into the right flank of the 8-week-old male A17^fl/fl^ and A17^α-MHCKO^ mice, respectively (Fig. 8h). These melanoma-bearing mice were randomly divided into four groups (*n* = 10/group): A17^fl/fl^ + NS, A17^fl/fl^ + DOX, A17^α-MHCKO^ + NS and A17^α-MHCKO^ + DOX. The injection method and doses of doxorubicin and normal saline were the same as previously described and these mice were euthanized 1 week after the last doxorubicin or saline injection. Tumor volumes (mm^3^) were measured with a caliper and calculated as V(mm^3^) = (0.5 × length × width^2^).^60^ At the end of the experiment, all tumors were dissected and weighted for analysis.

### RNA-sequencing analysis

The library construction and sequencing were performed by Sinotech Genomics Co., Ltd. (Shanghai, China). Total RNA was isolated using RNeasy mini kit (Qiagen, Germany) from mouse myocardial tissues, and the RNA concentration and quality were determined by the Qubit®3.0 Fluorometer (Life Technologies, USA) and the Nanodrop One spectrophotometer (Thermo Fisher Scientific Inc., USA). Paired-end libraries were synthesized by using the Stranded mRNA-seq Lib Prep Kit for Illumina (ABclonal, China) following preparation guide. Gene abundance was expressed as fragments per kilobase of exon per million reads mapped (FPKM). Stringtie software was used to count the fragments within each gene, and the TMM algorithm was used for normalization. Differential expression analysis for mRNA was performed using R package edgeR. Differentially expressed RNAs between A17^fl/fl^ + NS and A17^fl/fl^ + DOX, and between A17^α-MHCKO^ + DOX and A17^fl/fl^ + DOX mice with |log2(FC)| value > 1, *p* value < 0.05 and one group’s mean FPKM > 1, considered as significantly modulated, were retained for further analysis. We performed a Kyoto Encyclopedia of Genes and Genomes (KEGG) pathway analysis (http://www.genome.ad.jp/kegg) via enrich R package (version 3.4.3).

### High-pressure liquid chromatography (HPLC) analysis

High-pressure liquid chromatography (HPLC) was performed as described previously.^69,70^ Fifty microliters mouse serum was transferred to 1.5 mL polypropylene centrifuge tube, and then 150 μL methanol was added, the supernatant was collected after mixing and centrifugation. In addition, an appropriate amount of doxorubicin standard (MCE, USA) was used to make a reserve solution of 2 mg/mL and diluted with methanol into a series of standard curve working solutions with concentration gradients. These working solutions were diluted ten times with blank plasma and transferred to 1.5 mL polypropylene centrifuge tube with 50 μL of each solution, and then 150 μL methanol was added, which was vortex-mixed for 1 min and centrifuged at 13,000 rpm for 10 min. The supernatant was collected and the concentration of doxorubicin was quantitatively determined by high-pressure liquid chromatography–mass spectrometry (HPLC–MS). Chromatogram acquisition and integration of the compounds were processed by a software Xcalibur 3.0 (Thermo, USA), and linear regression was performed with a weighting coefficient (1/*X*^2^).

### Neonatal rat cardiomyocytes (NRCMs) isolation and culture

The ventricular muscle from 1- to 3-day-old Sprague Dawley rats was rapidly isolated and the heart tissue was minced to small pieces on ice, which were then digested with 0.75 mg/mL collagenase type II for 1 h at 37 °C.^71^ After repeated digestion for three times, the cells were harvested and centrifuged at 800 rpm for 5 min. The cell pellet was resuspended in the high-glucose Dulbecco’s modified Eagle medium (DMEM) supplemented with 8% horse serum, 5% newborn calf serum and 1% bromodeoxyuridine in plate for 2 h in a culture flask to let fibroblasts attach. Non-cardiomyocytes were removed by differential adherence and only non-attached cardiomyocytes were collected.

### Cell treatment

Cell treatment consisted of eight proportions. In the first proportion of the in vitro experiment (Supplementary Fig. 4a), in order to determine the optimal concentration and duration of doxorubicin treatment to induce cardiomyocyte apoptosis in vitro, NRCMs were treated with different concentrations of doxorubicin (0, 0.25, 0.5, 1, 2 and 4 μM) for 24 h or 1 μM doxorubicin for different time periods (0, 6, 12, 18, 24 and 36 h). Previous studies often used 1 μM of doxorubicin to stimulate NRCMs for 24 h.^72^ In addition, by stimulating NRCMs with different time periods and concentrations, we found that cell apoptosis was obvious and cell viability was not <50% when NRCMs were treated with 1 μM doxorubicin for 24 h. Therefore, NRCMs were stimulated with this regimen in subsequent experiments.

In the second proportion of the in vitro experiments (Supplementary Fig. 4b), to elucidate the role of ADAM17 silencing in cardiomyocyte, siRNA targeting ADAM17 (siA17) was transfected into NRCMs to knockdown the expression of ADAM17 and the control group was transfected by a negative control siRNA (siNC). These NRCMs were treated with dimethyl sulfoxide (DMSO) or 1 μM DOX for 24 h before cell collection.

In the third proportion of the in vitro experiments (Supplementary Fig. 4c), to explore the role of ADAM17 overexpression in cardiomyocyte, plasmid overexpressing ADAM17 was transfected into NRCMs (oeA17) and a negative control plasmid vector was transfected into the negative control NRCMs (NC). These two groups of NRCMs were treated with DMSO or 1 μM DOX for 24 h before cell collection.

In the fourth proportion of the in vitro experiments (Supplementary Fig. 4d), in order to verify the upstream and downstream relationship between ADAM17 and TRAF3, plasmid overexpressing ADAM17 (oeA17) and negative control plasmid vectors (NC) were first transfected into NRCMs, respectively. After 24 h, these NRCMs were transfected with siRNA targeting TRAF3 (siTRAF3) and siNC, respectively. Finally, NRCMs were treated with 1 μM DOX for 24 h before cell collection.

In the fifth proportion of the in vitro experiment (Supplementary Fig. 13a), to determine the optimal dose of TNF-α treatment for NRCMs, we treated the cells with different concentrations of TNF-α for 24 h, and selected the optimal concentration for subsequent experiments.

In the sixth proportion of the in vitro experiments (Supplementary Fig. 13b), to verify whether TNF-α induces downstream TRAF3 expression via TNFR, infliximab (MCE, USA), a chimeric monoclonal IgG1 antibody that specifically binds to TNF-α, was used to block the interaction of TNF-α with TNFR, and NRCMs were treated with NS, 10 μM TNF-α or 10 μM TNF-α + infliximab, respectively.

In the seventh proportion of the in vitro experiments (Supplementary Fig. 13c), to ascertain whether doxorubicin induces cardiac injury via upregulating TNF-α–TNFR signaling, NRCMs were pretreated with infliximab for 24 h before doxorubicin treatment, and then treated with DMSO, 1 μM DOX or 1 μM DOX + infliximab, respectively, for 24 h before cell collection.

In the eighth proportion of the in vitro experiment (Supplementary Fig. 13d), in order to verify that the downstream MAPKs pathway was activated by TAK1, NRCMs were treated with DMSO, 1 μM DOX or 1 μM DOX + TAK1 inhibitor 5Z-7-ox (MCE, USA), respectively, for 24 h before cell collection.

### Cell viability assay

NRCMs (4 × 10^3^ cells/well) were cultured in a 96-well plate and exposed to different concentrations (0, 0.25, 0.5, 1, 2 and 4 μM) of DOX for 24 h and to 1 μM DOX for different durations (0, 6, 12, 18, 24 and 36 h). After cell treatment, the culture medium was changed with a fresh medium. Then, 10 μL of CCK-8 was added to the NRCMs and the cells were cultured for 1 h at 37 °C. Optical density (OD) at 450 nm was measured by a microplate reader (BioTek, VT, USA). Cell viability = (OD of DOX cells − OD of blank control)/(OD of normal cells − OD of blank control) × 100%.

### Enzyme-linked immunosorbent assay (ELISA)

Serum TNF-α levels in mice were measured using a Mouse TNF-α ELISA Kit (ANRK, China). TNF-α levels in NRCMs culture supernatants were detected with a Rat TNF-α ELISA Kit (Proteintech, China).

### ADAM17 activity assay

ADAM17 activity was measured in mouse heart tissue and NRCMs using SensoLyte 520 TACE activity assay kit (AnaSpec, Fremont, CA). The heart tissue sample and NRCMs sample were homogenized in Component C containing 0.1% Triton X-100, which were incubated on ice for 15 min followed by centrifugation at 2000 × *g* for 15 min, and then the supernatant was collected. The biological samples were put into the plate and incubated for 10 min, and then ADAM17 substrate solution was added and incubated for 30 min at 37 °C. Terminating solution was then added to each hole and the absorbance at 490 nm/520 nm was measured using a microplate reader. The activity of each sample was measured and calculated by the formula of the standard curve.

### Statistical analysis

All data were presented as mean ± SEM. Shapiro–Wilk test was used to evaluate the normality distribution of the data. For the data with normal distribution, unpaired two-tailed Student’s *t*-test was used between two groups, and one-way ANOVA analysis followed by Tukey post-hoc test was performed among multiple groups. For data with a non-normal distribution, Mann–Whitney *U* test was used between two group comparisons, and Kruskal–Wallis test followed by Dunnett’s post-hoc test was used for multiple group comparisons. Kaplan–Meier curves and Log-Rank test were applied to assess animal survival data. In all statistical comparisons, *p* < 0.05 was considered statistically significant. All data analysis was performed using GraphPad Prism 8 (GraphPad, CA).

See Supplementary Materials for more details of experimental procedures.

## Supplementary information

supplementary material

western blot raw images

## Data Availability

Transcriptome RNA-sequencing datasets are deposited in Gene Expression Omnibus with an accession code of GSE276325. All data presented in this study are available from the corresponding author upon reasonable request.
