# Supplementary material for: Cardiomyocyte-specific knockout of ADAM17 alleviates doxorubicin-induced cardiomyopathy via inhibiting TNFα–TRAF3–TAK1–MAPK axis
Source: Signal Transduct Target Ther. 2024 Oct 16;9:273. doi: 10.1038/s41392-024-01977-z (PMC11480360; doi:10.1038/s41392-024-01977-z)
Supplement: Supplementary file 1 — supplementary material [file 41392_2024_1977_MOESM1_ESM.docx]

Supplemental Materials for

**Cardiomyocyte-specific knockout of ADAM17 alleviates doxorubicin-induced cardiomyopathy via inhibiting TNFα-TRAF3-TAK1-MAPK axis**

Lin Xie^1^, Fei Xue^1^, Cheng Cheng^1,7,^ Wenhai Sui^1^, Jie Zhang^1^, Linlin Meng^1^, Yue Lu^1^, Wenjing Xiong^1^, Peili Bu^1^, Feng Xu^3^, Xiao Yu^4^, Bo Xi^5^, Lin Zhong^6^, Jianmin Yang^1*^, Cheng Zhang^1,2*^, Yun Zhang^1,2*^

Correspondence to: Yun Zhang, E-mail: zhangyun@sdu.edu.cn, or Cheng Zhang, Email: zhangc@sdu.edu.cn or Jianmin Yang, E-mail: yangjianminsdu@163.com.

**This PDF file includes:**

Materials and methods

Supplementary Figures 1 to 20

Supplementary Tables 1 to 4

**Materials and methods**

***Ethics statement***

All animal experimental protocols complied with the Animal Management Rules of the Chinese Ministry of Health (Document no. 55, 2001) and conformed to National Institutes of Health (NIH) guidelines (the Guide for the Care and Use of Laboratory Animals; NIH Publication No. 85-23, revised 1996). All mice were maintained under specific pathogen-free, environmentally controlled (Temperature: 20–25 °C; humidity: 50 ± 5%) barrier conditions in individual ventilated cages and were fed with chow diet.

***Echocardiography***

Transthoracic echocardiography was performed using Visual Sonics Vevo 770 system connected with a 30 MHz high frequency MS400 transducer (Visual Sonics, Canada). Anesthesia with 5% isoflurane was performed, and the mice remained under general anesthesia with continuous inhalation of 2% isoflurane during echocardiogram acquisition. Parasternal long-axis view was imaged to derive left ventricular M-mode echocardiogram and the following parameters were measured: systolic and diastolic left ventricular internal diameter (LVIDs and LVIDd), systolic and diastolic left ventricular posterior wall (LVPWs and LVPWd), systolic and diastolic interventricular septum thickness (IVSs and IVSd), left ventricular ejection fraction (LVEF), left ventricular fractional shortening (LVFS). The early (E) and late (A) diastolic mitral flow velocities were measured by pulsed Doppler in the four-chamber view. The early (E’) and late (A’) diastolic mitral annular velocities were measured by tissue Doppler imaging in the four-chamber view. The ratio of early to late diastolic mitral flow velocities (E/A), ratio of early to late diastolic mitral annular velocities (E'/A'), and ratio of early diastolic transmittal flow velocity to early diastolic mitral annular velocity (E/E') were calculated. The detailed echocardiographic measurements were listed in Supplementary Table 2.

***Histology and immunofluorescence***

Formalin-fixed and paraffin-embedded cardiac tissues were cut into 5μm thick sections for subsequent analyses. Masson’s trichrome staining and hematoxylin and eosin (H&E) staining were performed according to the manufacturer’s instructions using staining kits (Solarbio, Beijing, China). Fluorescein isothiocyanate-conjugated wheat germ agglutinin (FITC-conjugated WGA) staining (Sigma-Aldrich, USA) was used to measure the cardiomyocyte cross-sectional size. For immunofluorescent staining of tissues, sections were dewaxed and subjected to the antigen retrieval with citrate buffer, which were then blocked with 5% bovine serum albumin (BSA) for 30 min and incubated with primary antibodies at 4°C overnight. For immunofluorescent staining of cells, the cells were fixed in 4% paraformaldehyde for 15 min, blocked with 5% BSA, and incubated with primary antibodies at 4°C overnight. The primary antibodies used were listed in Supplementary Table 3, and sections and cell coverslips reacting with non-immune IgG as well as secondary antibodies were used as negative control. On the next day, sections or cell coverslips were incubated with Alexa Fluor 488/594-conjugated secondary antibodies (Abcam, Cambridge, UK). The cell nuclei were stained with 4,6-diamidino-2-phenylindole (DAPI, Abcam, Cambridge, UK). Immunofluorescent staining was observed and photographed using a fluorescent microscope (Ti-S, Nikon). All histological images were examined and photographed under a microscope (Ti-S, Nikon). Cardiomyocyte cross-sectional area and cardiac fibrotic area were quantified using Image J software and the histological analysis for each mouse were averaged across at least 5 representative areas.

***Gene silencing by small interfering RNA (siRNA)***

The siRNA of ADAM17, TRAF3, C/EBPβ, TNFR1, TNFR2 or negative control siRNA were obtained from keyybio, Shandong, China. NRCMs were cultured in antibiotic-free medium. The ADAM17-siRNA, TRAF3-siRNA, C/EBPβ-siRNA, TNFR1-siRNA, TNFR2-siRNA and NC-siRNA were delivered to the NRCMs by Lipofectamine iMAX reagent (Invitrogen, Carlsbad, CA) according to the manufacturer's protocol. After transfection for 24 h, the supernatant was replaced with fresh medium. The siRNA sequence was as follows: ADAM17 5’--3’: CGAGTTGATAGCAAAGAGA, TRAF3 5’--3’: GTACAAACCAGCAGATCAA, C/EBPβ 5’--3’: ACCTCTTCGCCGACGACTA. TNFR1 5’--3’: GGTGGAGGGTGAAGGAATT, and TNFR2 5’--3’: CAATAGGCCTTGAACAGCA.

***ADAM17 overexpression in NRCMs***

ADAM17 overexpressing plasmid or negative control plasmid was obtained from keyybio, Shandong, China. Lipofectamine 3000 reagents (Invitrogen, USA) were used for transfection of ADAM17 pcDNA3.1-3×Flag-C or pcDNA3.1-3×Flag-C into NRCMs according to the manufacturer’s instructions. After transfection for 24 h, the supernatant was replaced with fresh medium.

***Co-immunoprecipitation assay***

Human HEK293T cells and NRCMs were used for exogenous and endogenous Co-immunoprecipitation (Co-IP) assay, respectively. HEK293T cells were obtained from KeyGene BioTech (China) and cultured in complete medium: DMEM supplemented with 10% FBS, and 1% penicillin–streptomycin. HEK293T cells were cultured in a 60mm cell dish and collected 24h after transfection with Myc-TRAF3 and Flag-TAK1 overexpressing plasmids. NRCMs were cultured in a 100mm cell dish and treated with DOX. After culture for 24 h, the cells were lysed with RIPA lysis buffer (CWbio, China) supplemented with a protease inhibitor cocktail (CWbio, China) on ice for 30 min. After centrifugation, the supernatants were transferred into a new tube and incubated with 1ug primary antibodies or IgG (Abcam, Cambridge, UK) for 2 h at 4°C. Protein A/G-agarose beads (Santa Cruz Biotechnology, USA) were incubated with the mixture overnight at 4°C. On the second day, the beads were washed for five times through centrifugation with lysis buffer, and immunoprecipitated proteins were subjected to western blot analysis after incubation with loading buffer at 99°C for 10min.

***Co-localization of TdT-mediated dUTP nick end-labeling (TUNEL) staining and cardiomyocyte markers***

Myocardial sections (5μm thick) were dewaxed and subjected to the antigen retrieval with citrate buffer, which were then blocked with 5% bovine serum albumin (BSA) for 30 min at 37°C and incubated with the corresponding primary antibodies against cTnT at 4°C overnight. Thereafter, the tissue sections were incubated with appropriate fluorescent secondary antibodies for 30 min at 37°C after washing with PBS. Apoptotic cells in the myocardium were detected via TUNEL assay, performed using a commercially available kit (In Situ Cell Death Detection Kit, TMR red; Roche) following the manufacturer's instructions, subsequently sealed with DAPI tablet. The cell coverslips of NRCMs were fixed with 4% paraformaldehyde at room temperature for 20 min and washed by PBS for three times. Then, cells were permeabilized in PBS with 0.1% Triton X-100 and stained with TUNEL reaction mixture for 1h at 37°C. Finally, the cells were counterstained with DAPI. The images were acquired with a fluorescence microscope (Ni-E, Nikon, Japan) with an excitation wavelength. The apoptosis ratio was expressed as the proportion of apoptotic cells to the total number of cells.

***Western blot analysis***

Total protein was extracted from heart tissues using the Total Protein Extraction Kit (Invent Biotechnologies, Plymouth, MN, USA), and protein was extracted from NRCMs using RIPA lysis buffer. Equal amounts of extracted protein samples were separated using 10% sodium dodecyl sulfate-polyacrylamide gel electrophoresis, and the resolved protein bands were transferred to a polyvinylidene fluoride membrane (Millipore, MA, USA). After incubation in 5% bovine serum albumin for 1 hour at room temperature, the membranes were incubated with primary antibodies at 4°C overnight. Following incubation with peroxidase-conjugated secondary antibodies (1:5000, Jackson ImmunoResearch Laboratories, PA, USA) at room temperature for 1 h, the protein bands were detected using a chemiluminescent substrate (Millipore, MA, USA) and exposure to a chemiluminescence instrument (GE, Amersham Imager 800RGB). The primary antibodies were listed in Supplementary Table 3.

***Quantitative real-time polymerase chain reaction***

Total RNA was extracted from isolated heart tissue by means of the RNeasy mini kit (Qiagen, 74704, Germany) which was reversed-transcribed using a PrimeScript RT reagent kit with gDNA Eraser (TaKaRa, Japan), and quantitative real-time RT-PCR was performed utilizing Takara SYBR RT-PCR kits according to the manufacturer's instructions. Cycling conditions were: 95°C for 10 min, and 95°C for 15s, 55°C for 15s, and 72°C for 20s for 40 cycles. Data were normalized by the level of β-actin expression in each individual sample, and the 2−ΔΔCt method was used to calculate relative expression changes. The primer sequences were listed in Supplementary Table 4.

**
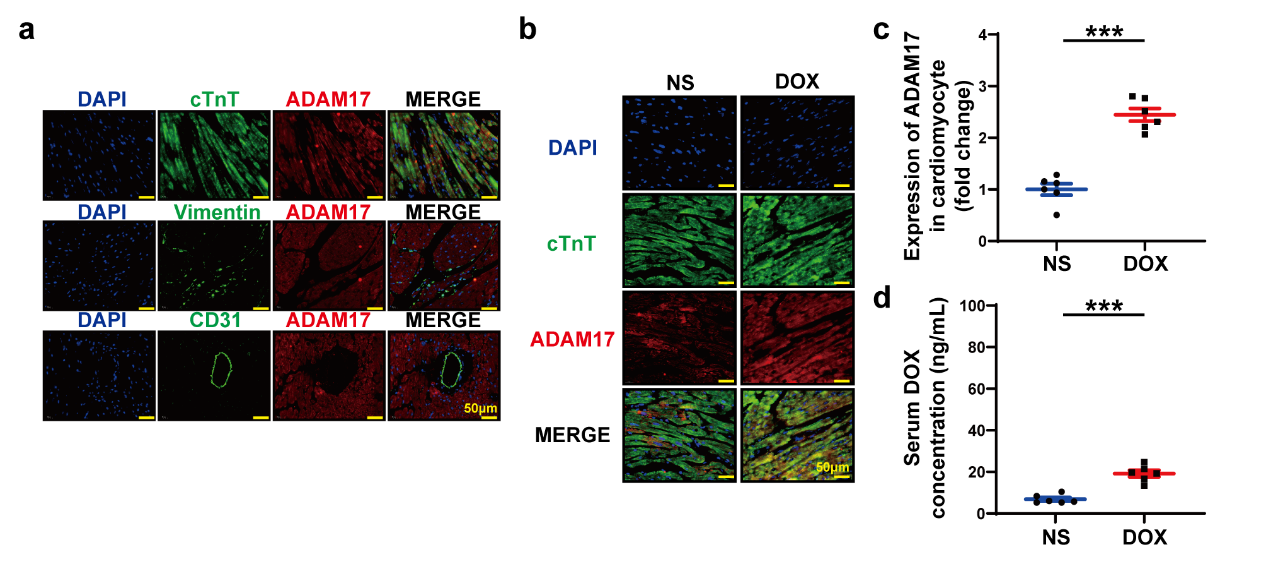
**

**Supplementary Figure 1. ADAM17 expression in the cardiomyocyte and doxorubicin concentration in serum of mice treated with NS or DOX. a** Representative immunofluorescence staining of ADAM17 (red) in different cardiac cells (green). cTnT, Vimentin and CD31 were used as markers for cardiomyocytes, fibroblasts and endothelial cells, respectively (scale bar=50μm). **b-c** Representative immunofluorescence staining (scale bar=50μm) and quantitative analysis of ADAM17 protein expression in the cardiomyocytes of mice treated with NS or DOX (n=6 in each group). **d** Quantitative analysis of doxorubicin concentration in the serum of mice treated with NS or DOX (n=6 in each group). Values shown were mean and SEM. Unpaired two-tailed Student’s t test were applied in **c** and **d**. ********p* <0.001.

**
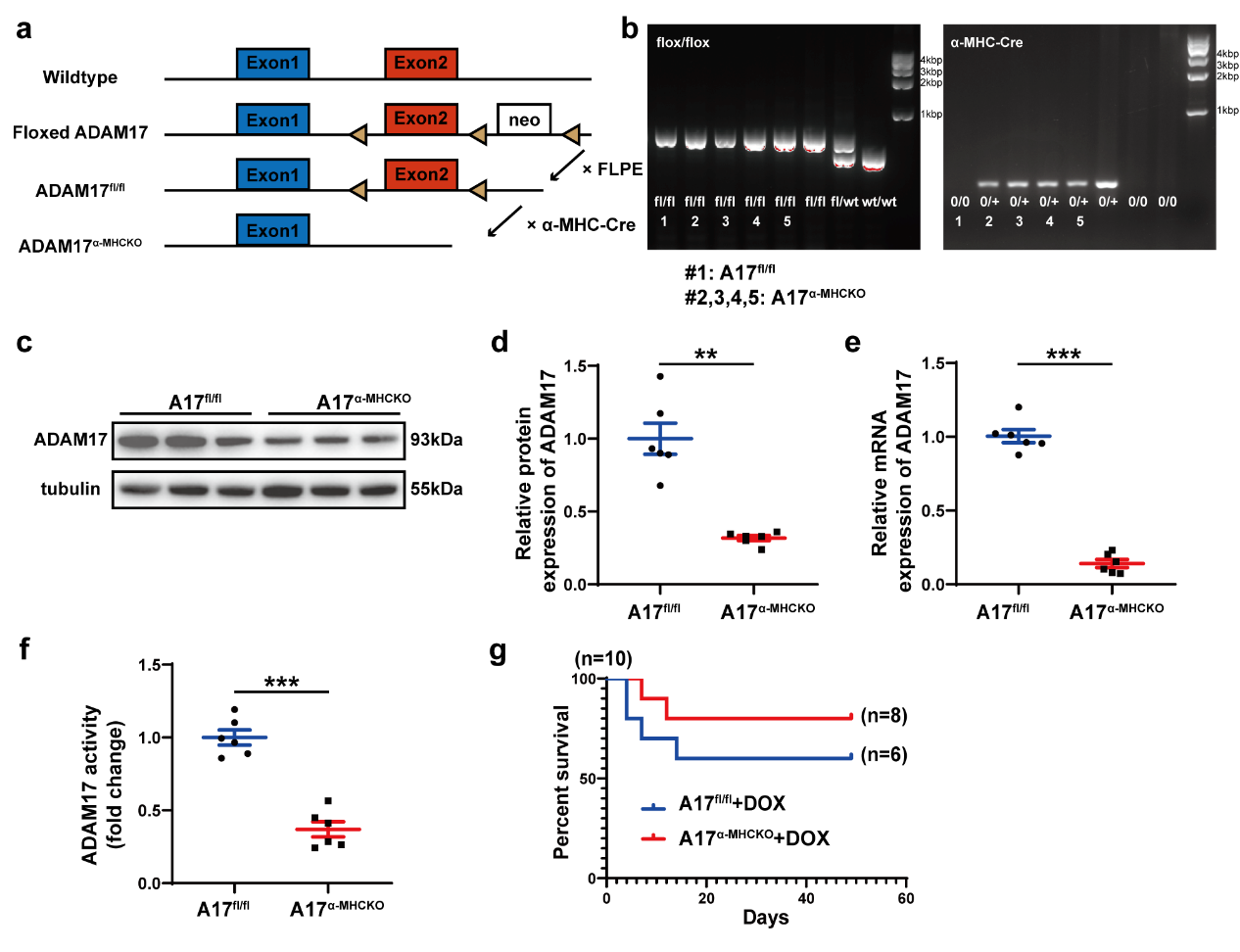
**

**Supplementary Figure 2. Generation and identification of cardiomyocyte-specific ADAM17 knockout mice, and the survival curve of A17^fl/fl^ mice and A17^α-MHCKO^ mice treated with doxorubicin. a** Schematic diagram showing the breeding strategy. After the introduction of cre-recombinase (α-MHC-cre), the ADAM17 gene exon 2 was specifically excised in cardiomyocytes, resulting in generation of selective ADAM17 knockout mice (A17^α-MHCKO^). **b** Representaive genotyping results for A17^α-MHCKO^ and control littermates. Pups 2,3,4 and 5 were identified as A17^α-MHCKO^ [A17^fl/fl^ with cre recombinase (Cre0/+)], and pups 1 was control littermates [A17^fl/fl^ without cre recombinase (Cre0/0)]. **c-d** Representative western blot images and comparison of ADAM17 protein expression in the hearts of the A17^fl/fl^ and A17^α-MHCKO^ mice (n=6 in each group). **e** Comparison of ADAM17 mRNA expression in the hearts of the A17^fl/fl^ and A17^α-MHCKO^ mice (n=6 in each group). **f** Comparison of ADAM17 activity in the hearts of the A17^fl/fl^ and A17^α-MHCKO^ mice (n=6 in each group). **g** The survival curve of A17^fl/fl^ and A17^α-MHCKO^ mice treated with doxorubicin (n=10 in each group). Values shown were mean and SEM. Unpaired two-tailed Student’s t test were applied in **d, e** and **f**. Log-rank test was applied in **g**. *******p* <0.01; ********p* <0.001.

**
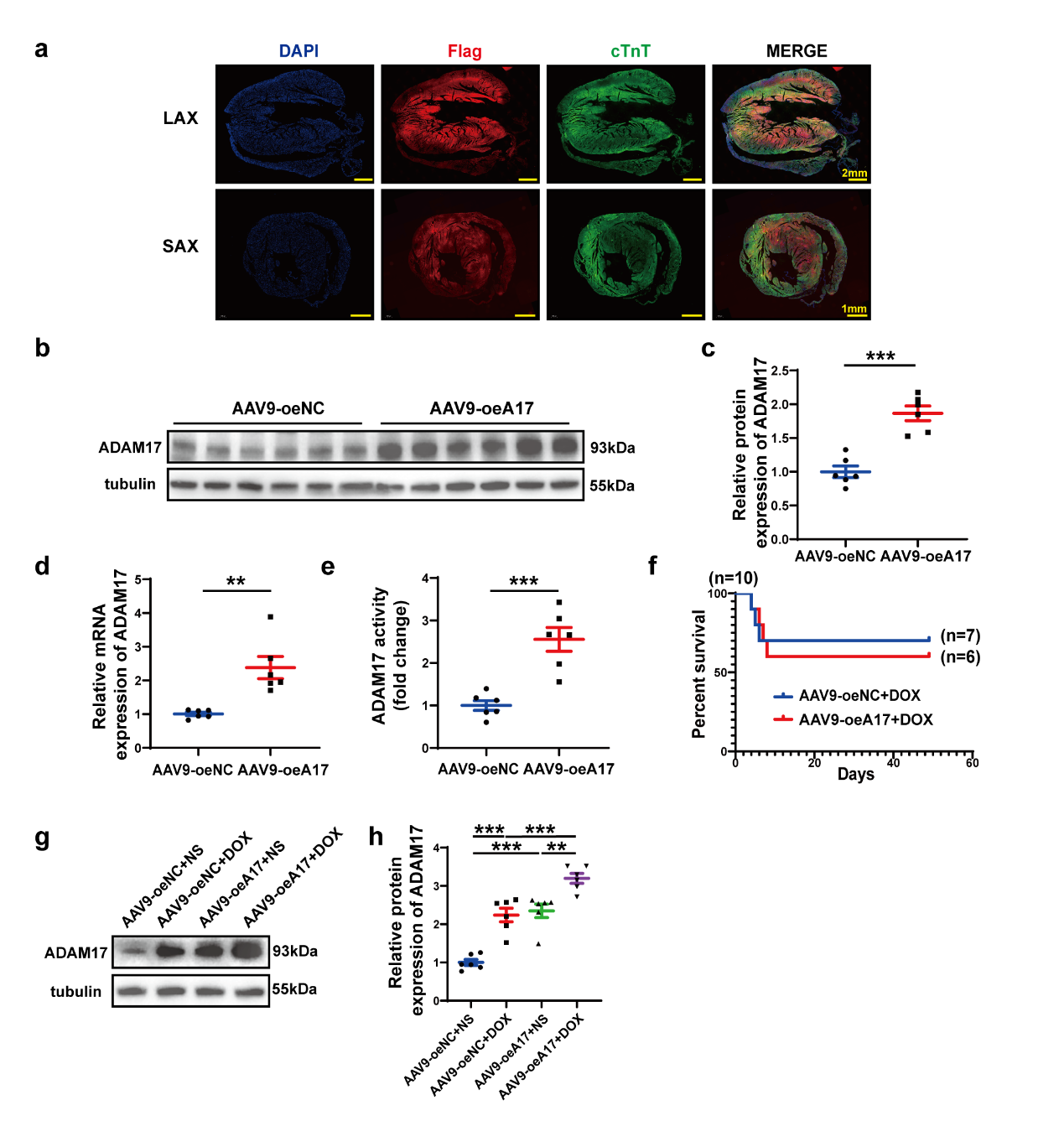
**

**Supplementary Figure 3. ADAM17 expression and survival rate in AAV9-oeNC and AAV9-oeA17 mice. a** Representative immunofluorescence staining images of Flag in the heart of AAV9-oeA17 mice. **b-c** Representative western blot images and comparison of ADAM17 protein expression in the hearts of the AAV9-oeNC and AAV9-oeA17 mice (n=6 in each group). **d** Comparison of ADAM17 mRNA expression in the hearts of the AAV9-oeNC mice and AAV9-oeA17 mice (n=6 in each group). **e** Comparison of ADAM17 activity in the hearts of the AAV9-oeNC mice and AAV9-oeA17 mice (n=6 in each group). **f** Survival curve of AAV9-oeNC and AAV9-oeA17 mice treated with doxorubicin (n=10 in each group). **g-h** Representative western blot images and comparison of ADAM17 protein expression in the myocardium among four groups of mice. Values shown were mean and SEM. Unpaired two-tailed Student’s t test were applied in **c, d** and **e**, Log-rank test was applied in **f**, One-way ANOVA were applied in **h. *****p* <0.01; ********p* <0.001.


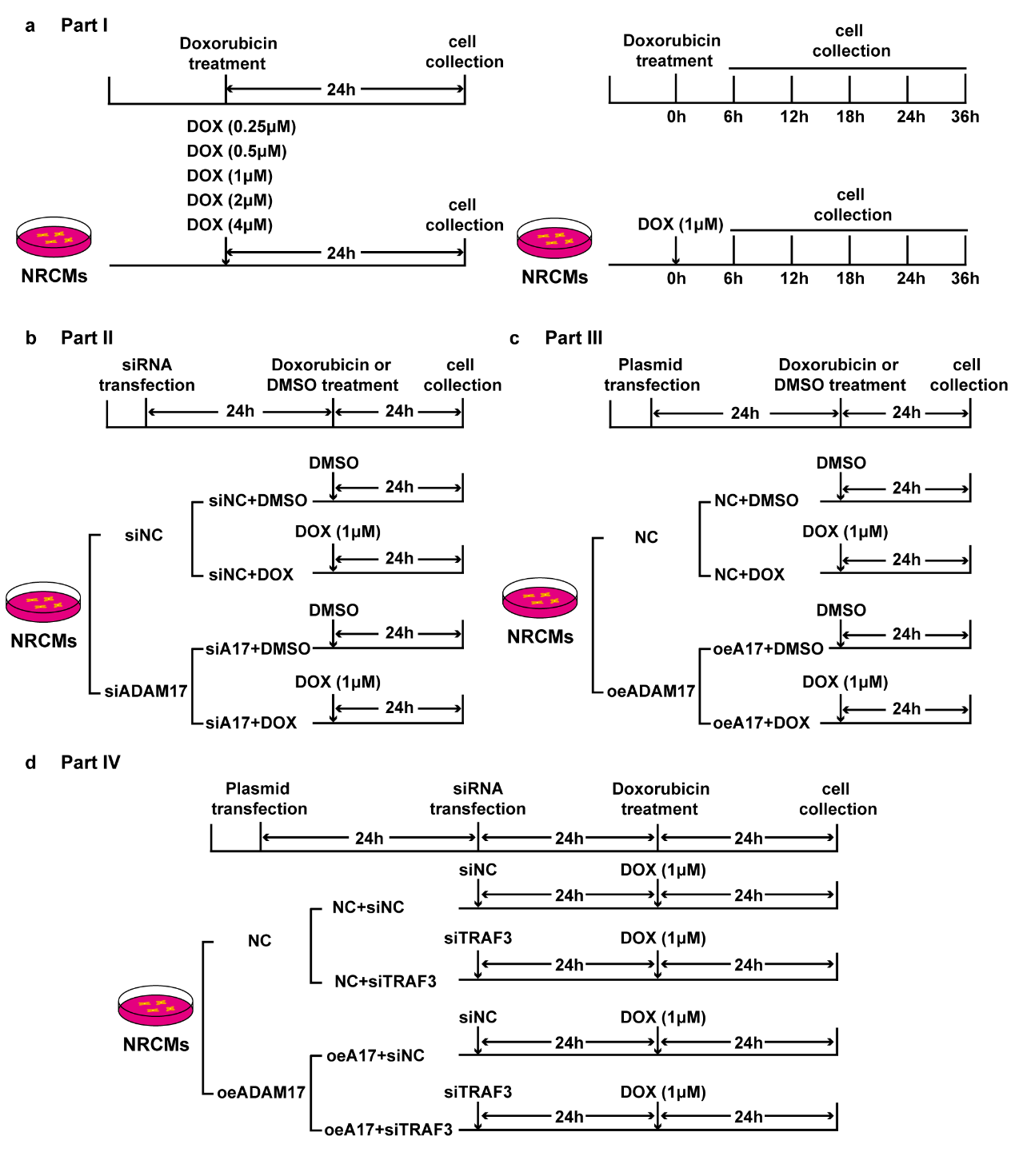


**Supplementary Figure 4. Experiment timeline *in vitro*.**


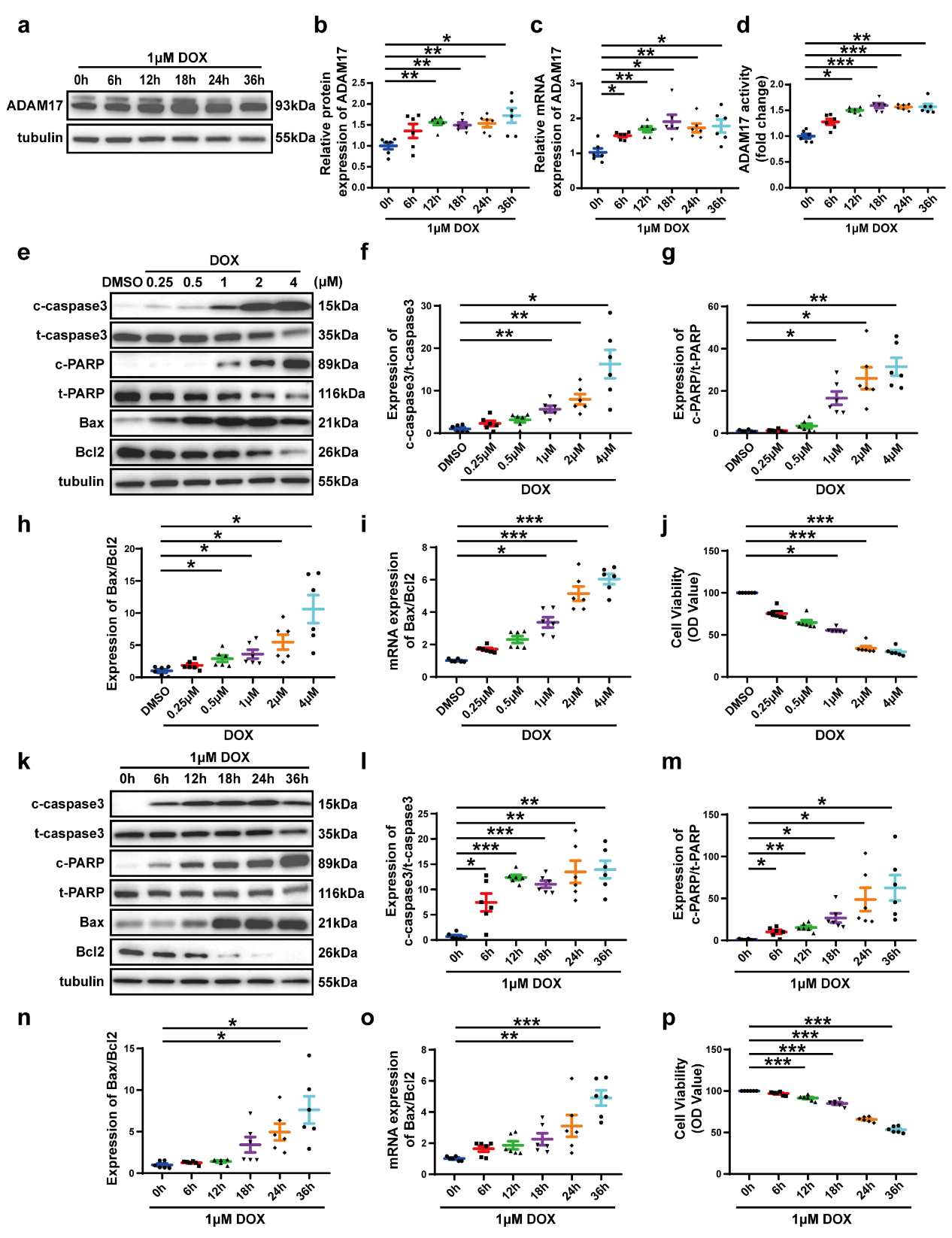


**Supplementary Figure 5. Protein and mRNA expression of ADAM17 and apoptosis-related molecules in doxorubicin-treated NRCMs. a** Representative western blot images of ADAM17 protein expression in the NRCMs treated with 1μM DOX for different durations. **b** Comparison of ADAM17 protein expression in the NRCMs treated with 1μM DOX for different durations (n=6 in each group). **c** Comparison of ADAM17 mRNA expression in NRCMs treated with 1μM DOX for different durations (n=6 in each group). **d** Comparison of ADAM17 activity in NRCMs treated with 1μM DOX for different durations (n=6 in each group). **e** Representative western blot images of caspase3, cleaved caspase3, PARP, cleaved PARP, Bax and Bcl2 in the NRCMs treated with doxorubicin at different concentrations for 24h. **f** Comparison of cleaved caspase3/caspase3 protein expression among NRCMs treated with doxorubicin at different concentrations for 24h (n=6 in each group). **g** Comparison of cleaved PARP/PARP protein expression among NRCMs treated with doxorubicin at different concentrations for 24h (n=6 in each group). **h** Comparison of Bax/Bcl2 protein expression among NRCMs treated with doxorubicin at different concentrations for 24h (n=6 in each group). **i** Comparison of Bax/Bcl2 mRNA expression among NRCMs treated with doxorubicin at different concentrations for 24h (n=6 in each group). **j** Cell viability in six groups of NRCMs treated with doxorubicin at different concentrations for 24h (n=6 in each group). **k** Representative western blot images of caspase3, cleaved caspase3, PARP, cleaved PARP, Bax and Bcl2 protein expression in NRCMs treated with 1μM doxorubicin for different durations. **l** Comparison of cleaved caspase3/caspase3 protein expression among NRCMs treated with 1μM doxorubicin for different durations (n=6 in each group). **m** Comparison of cleaved PARP/PARP protein expression among NRCMs treated with 1μM doxorubicin for different durations (n=6 in each group). **n** Comparison of Bax/Bcl2 protein expression among NRCMs treated with 1μM doxorubicin for different durations (n=6 in each group). **o** Comparison of Bax/Bcl2 mRNA expression among NRCMs treated with 1μM doxorubicin for different durations (n=6 in each group). **p** Cell viability in six groups of NRCMs treated with 1μM doxorubicin for different durations (n=6 in each group). Values shown were mean and SEM. One-way ANOVA were applied in **b, c, d, f, g, h, l, m, n** and **p.** Kruskal-Walli’s test were applied in **i, j** and **o. ****p* <0.05; *******p* <0.01; ********p* <0.001.

**
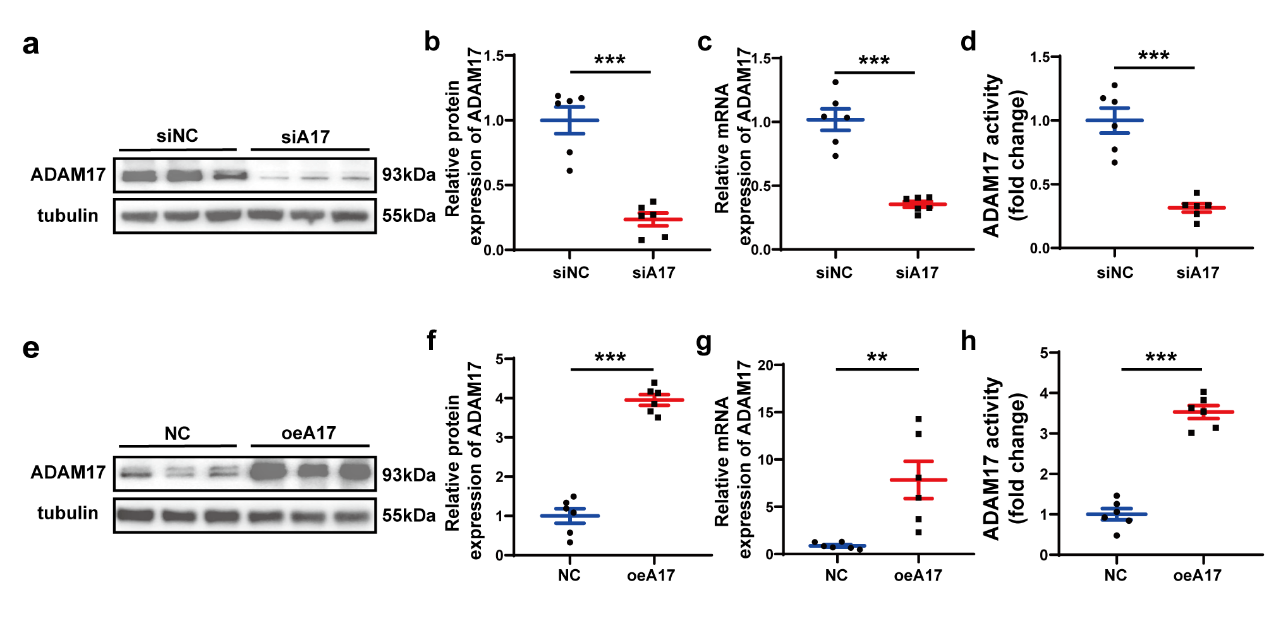
**

**Supplementary Figure 6. Efficiency of ADAM17 knockdown and overexpression in NRCMs. a** Representative western blot images of protein expression of ADAM17 in two groups of NRCMs treated with NC-siRNA and ADAM17-siRNA (n=6 in each group). **b-c** Comparison of ADAM17 protein and mRNA expression in two groups of NRCMs treated with NC-siRNA and ADAM17-siRNA (n=6 in each group). **d** Comparison of ADAM17 activity in two groups of NRCMs treated with NC-siRNA and ADAM17-siRNA (n=6 in each group). **e** Representative western blot images of protein expression of ADAM17 in two groups of NRCMs treated with a negative control plasmid and an ADAM17-overexpressing plasmid, respectively (n=6 in each group). **f-g** Comparison of ADAM17 protein and mRNA expression in two groups of NRCMs treated with a negative control plasmid and an ADAM17-overexpressing plasmid, respectively (n=6 in each group). **h** Comparison of ADAM17 activity in two groups of NRCMs treated with a negative control plasmid and an ADAM17-overexpressing plasmid, respectively (n=6 in each group). Values shown were mean and SEM. Unpaired two-tailed Student’s t test were applied in **b, c, d,** **f, g** and **h**. *******p* <0.01; ********p* <0.001.

**
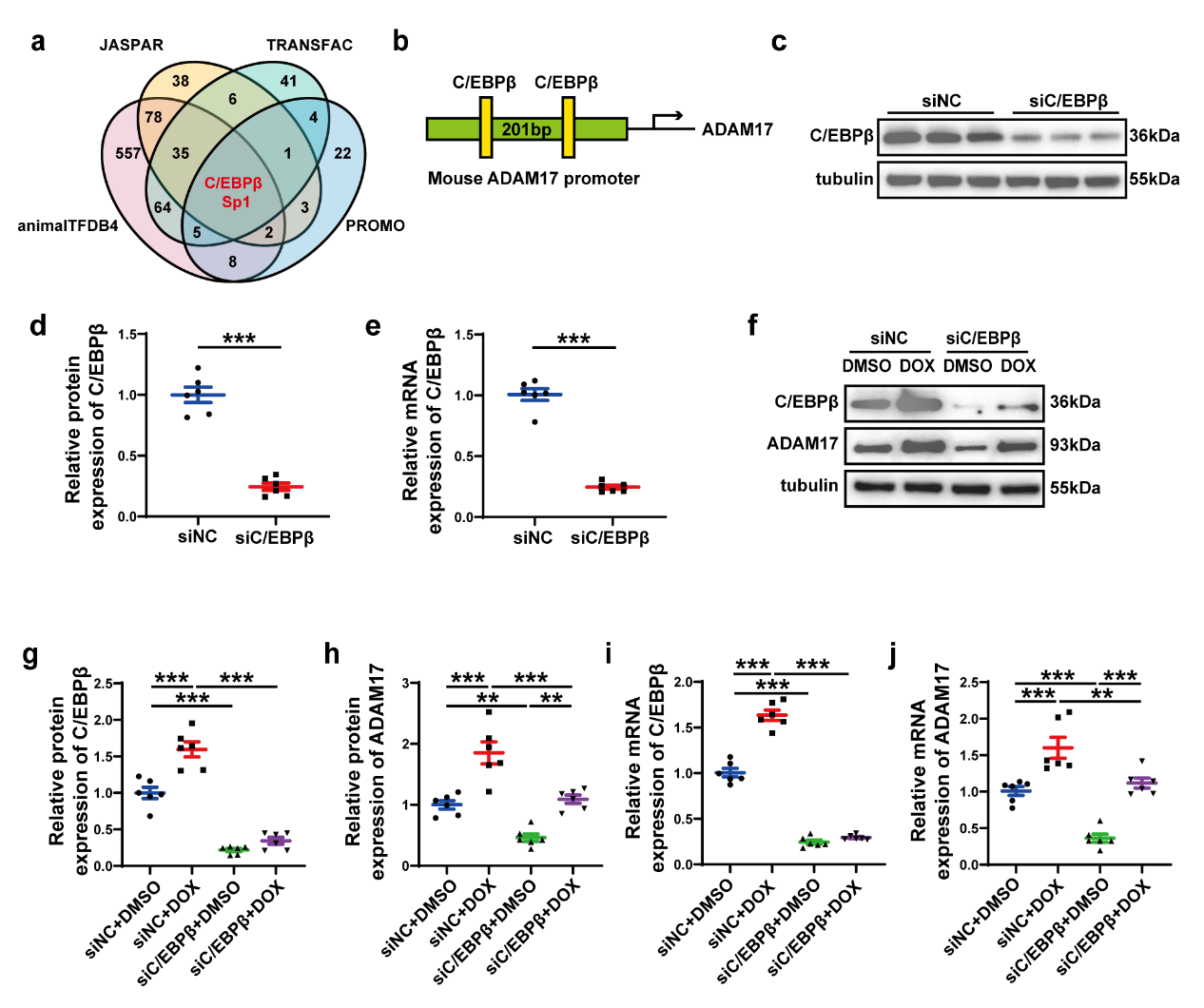
**

**Supplementary Figure 7. Effect of C/EBPβ knockdown on ADAM17 expression in NRCMs treated with DMSO or DOX. a** Venn diagram showing the overlap of predicted transcription factors in Animal TFDB4, JASPAR, TRANSFAC and PROMO databases that may bind to ADAM17 promotors. The numbers represented the number of transcription factors that overlap between databases. The transcription factors predicted by all four databases were C/EBPβ and Sp1. **b** Diagrammatic drawing of C/EBPβ binding to the ADAM17 promoter. **c** Representative western blot images of protein expression of C/EBPβ in two groups of NRCMs treated with NC-siRNA and C/EBPβ-siRNA, respectively (n=6 in each group). **d-e** Comparison of C/EBPβ protein and mRNA expression in two groups of NRCMs treated with NC-siRNA and C/EBPβ-siRNA, respectively (n=6 in each group). **f** Representative western blot images ADAM17 and C/EBPβ protein expression in NRCMs treated with siNC + DMSO, siNC + DOX, siC/EBPβ+DMSO, and siC/EBPβ+DOX, respectively. **g** Comparison of C/EBPβ protein expression in NRCMs treated with siNC + DMSO, siNC + DOX, siC/EBPβ+DMSO, and siC/EBPβ+DOX, respectively (n=6 in each group). **h** Comparison of ADAM17 protein expression in NRCMs treated with siNC + DMSO, siNC + DOX, siC/EBPβ+DMSO, and siC/EBPβ+DOX, respectively (n=6 in each group). **i** Comparison of C/EBPβ mRNA expression in NRCMs treated with siNC + DMSO, siNC + DOX, siC/EBPβ+DMSO, and siC/EBPβ+DOX, respectively (n=6 in each group). **j** Comparison of ADAM17 mRNA expression in NRCMs treated with siNC + DMSO, siNC + DOX, siC/EBPβ+DMSO, and siC/EBPβ+DOX, respectively (n=6 in each group). Values shown were mean and SEM. Unpaired two-tailed Student’s t test were applied in **d** and **e**, One-way ANOVA were applied in **g, h, i** and **j.** *******p* <0.01; ********p* <0.001.

**
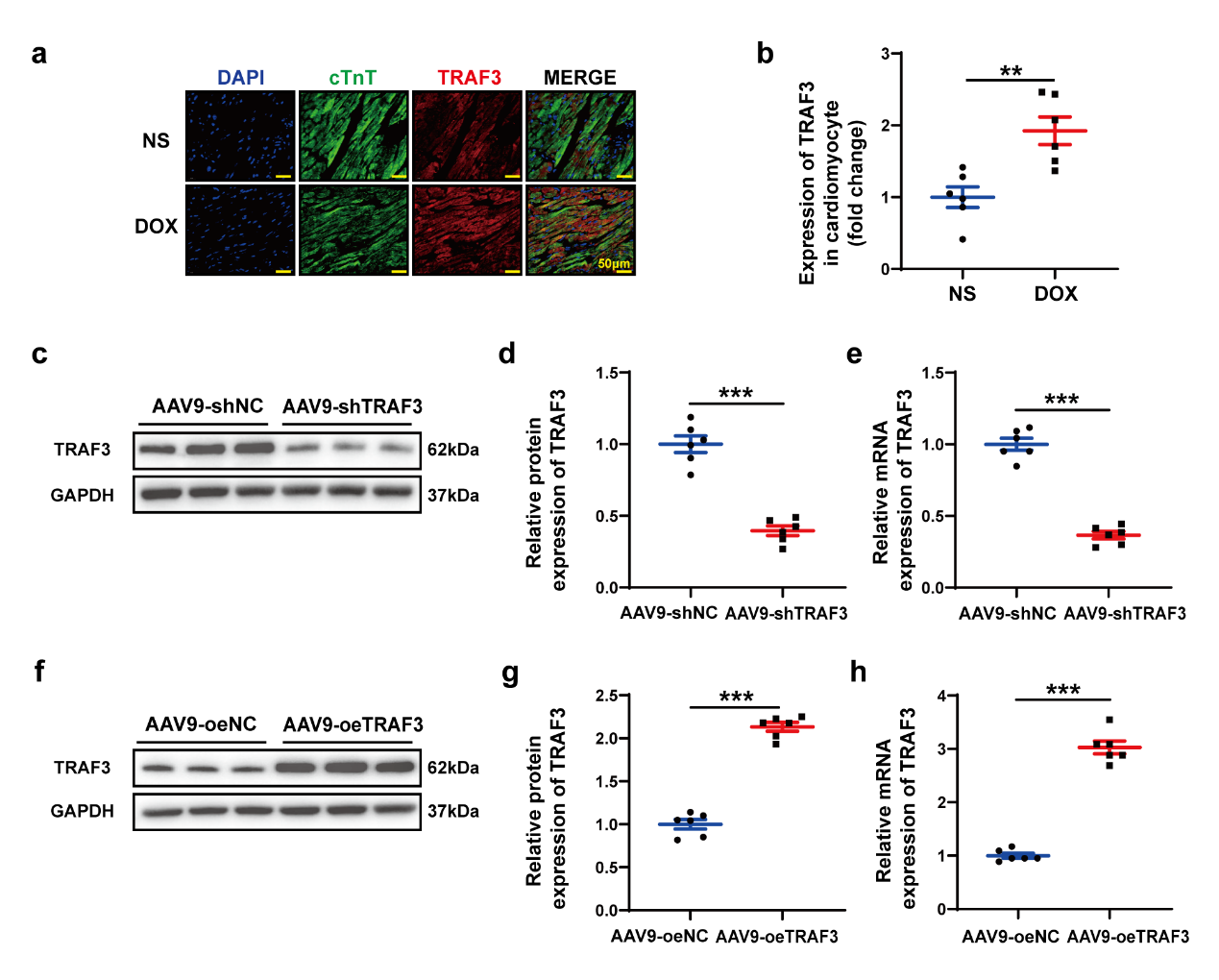
**

**Supplementary Figure 8.** **TRAF3 expression in the cardiomyocytes of mice treated with NS or DOX and efficiency of TRAF3 knockdown and overexpression in mice. a-b** Representative immunofluorescence staining (scale bar=50μm) and quantitative analysis of TRAF3 protein expression in the cardiomyocytes of mice treated with NS or DOX (n=6 in each group). **c-d** Representative western blot images and comparison of TRAF3 protein expression in the hearts of the AAV9-shNC and AAV9-shTRAF3 mice (n=6 in each group). **e** Comparison of TRAF3 mRNA expression in the hearts of the AAV9-shNC and AAV9-shTRAF3 mice (n=6 in each group). **f-g** Representative western blot images and comparison of TRAF3 protein expression in the hearts of the AAV9-oeNC and AAV9-oeTRAF3 mice (n=6 in each group). **h** Comparison of TRAF3 mRNA expression in the hearts of the AAV9-oeNC and AAV9-oeTRAF3 mice (n=6 in each group). Values shown were mean and SEM. Unpaired two-tailed Student’s t test were applied in **b, d, e, g** and **h**. *******p* <0.01; ********p* <0.001.

**
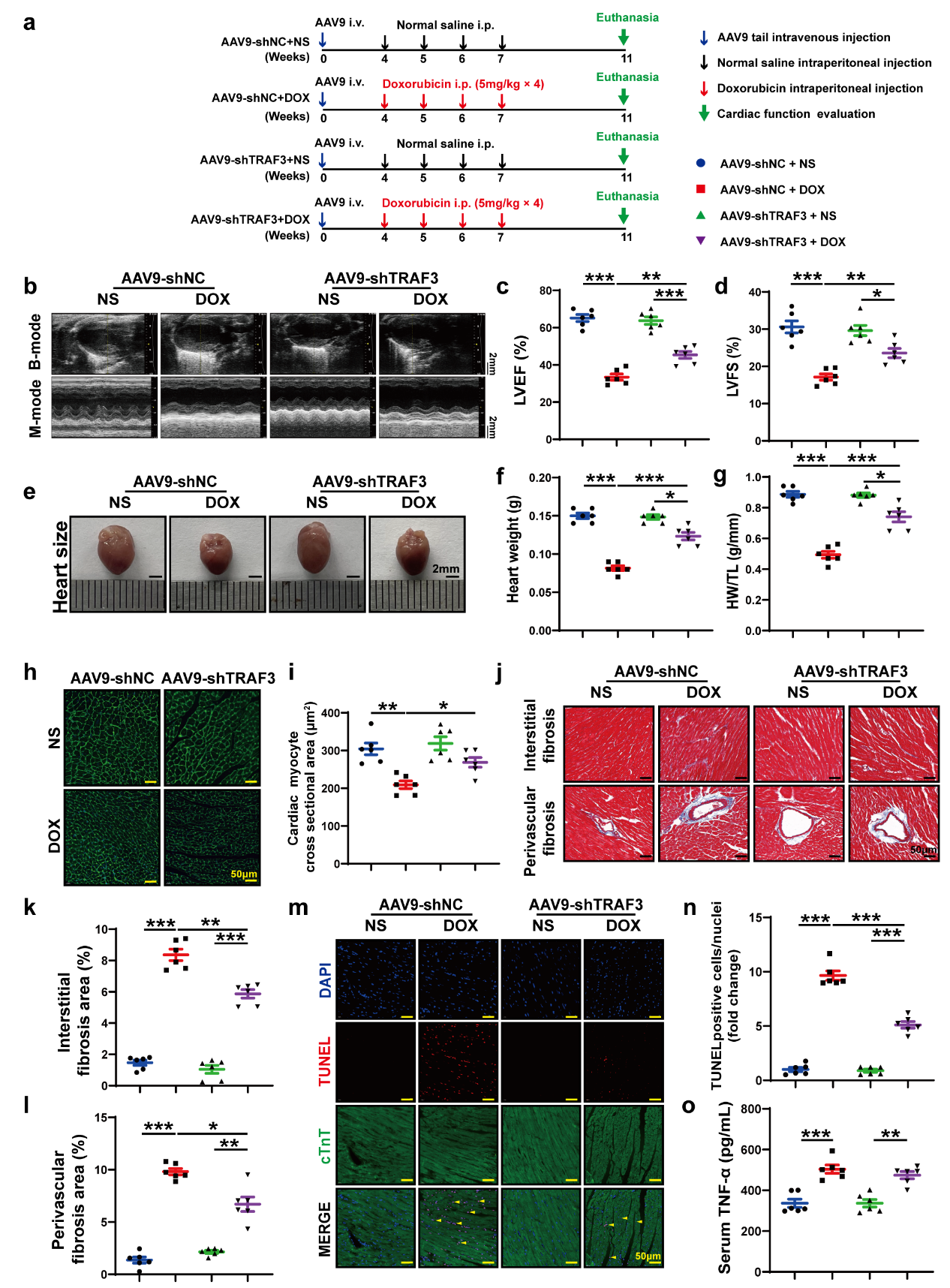
**

**Supplementary Figure 9. Echocardiographic measurements and histological staining in negative control and TRAF3 knockdown mice treated with NS or DOX. a** Experiment timeline *in vivo*. **b** Representative echocardiographic images (scale bar=2mm) showing B-mode and M-mode echocardiograms in four groups of mice. **c** Comparison of left ventricular ejection fraction (LVEF) among four groups of mice (n=6 in each group). **d** Comparison of left ventricular fractional shortening (LVFS) among four groups of mice (n=6 in each group). **e** Representative anatomical images of heart size (scale bar=2mm) in four groups of mice. **f** Comparison of heart weight among four groups of mice (n=6 in each group). **g** Comparison of heart weight/tibial length (HW/TL) ratio among four groups of mice (n=6 in each group). **h** Representative WGA staining of myocardial cross-sections (scale bar=50μm) in four groups of mice. **i** Comparison of cardiomyocyte cross-sectional area measured by WGA staining (n=6 in each group). **j** Representative Masson’s trichrome staining of myocardial interstitial and perivascular fibrosis (scale bar=50μm) in four groups of mice. **k** Comparison of interstitial fibrosis area among four groups of mice (n=6 in each group). **l** Comparison of perivascular fibrosis area among four groups of mice (n=6 in each group). **m** Representative TUNEL-positive cardiomyocyte staining in four groups of mice (scale bar=50μm). **n** Comparison of TUNEL-positive cardiomyocyte in the myocardium among four groups of mice (n=6 in each group). **o** Serum levels of TNF-α in four groups of mice (n=6 in each group). Values shown were mean and SEM. One-way ANOVA were applied in **c, d, f, g, i, k, l, n** and **o. ****p* <0.05; *******p* <0.01; ********p* <0.001.

**
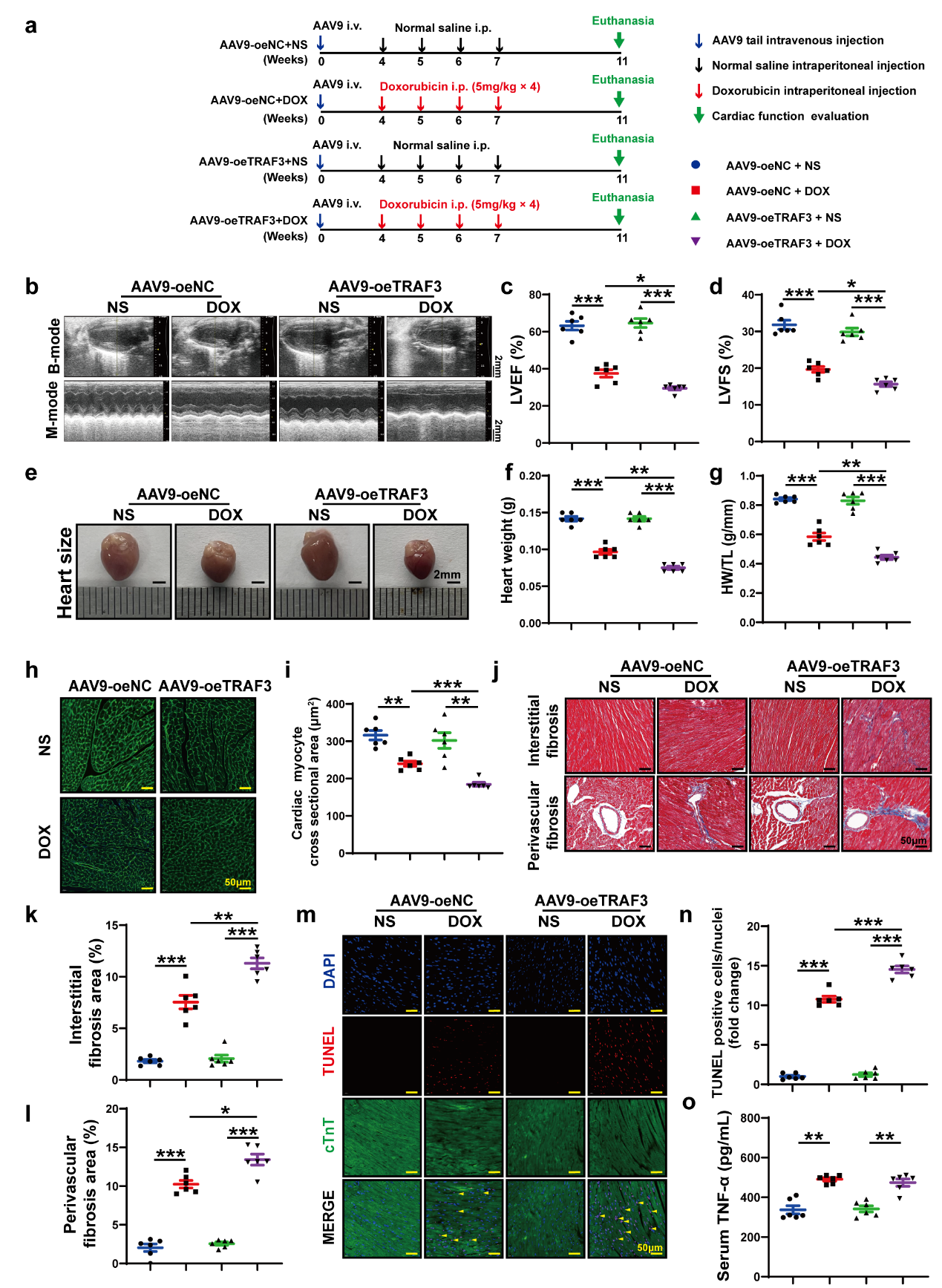
**

**Supplementary Figure 10. Echocardiographic measurements and histological staining in negative control and TRAF3-overexpressing mice treated with NS or DOX. a** Experiment timeline *in vivo*. **b** Representative echocardiographic images (scale bar=2mm) showing B-mode and M-mode echocardiograms in four groups of mice. **c** Comparison of left ventricular ejection fraction (LVEF) among four groups of mice (n=6 in each group). **d** Comparison of left ventricular fractional shortening (LVFS) among four groups of mice (n=6 in each group). **e** Representative anatomical images of heart size (scale bar=2mm) in four groups of mice. **f** Comparison of heart weight among four groups of mice (n=6 in each group). **g** Comparison of heart weight/tibial length (HW/TL) ratio among four groups of mice (n=6 in each group). **h** Representative WGA staining of myocardial cross-sections (scale bar=50μm) in four groups of mice. **i** Comparison of cardiac myocyte cross-sectional area measured by WGA staining (n=6 in each group). **j** Representative Masson’s trichrome staining of myocardial interstitial and perivascular fibrosis (scale bar=50μm) in four groups of mice. **k** Comparison of interstitial fibrosis area among four groups of mice (n=6 in each group). **l** Comparison of perivascular fibrosis area among four groups of mice (n=6 in each group). **m** Representative TUNEL-positive cardiomyocyte staining in four groups of mice (scale bar=50μm). **n** Comparison of TUNEL-positive cardiomyocytes in the myocardium among four groups of mice (n=6 in each group). **o** Serum levels of TNF-α in four groups of mice (n=6 in each group). Values shown were mean and SEM. One-way ANOVA were applied in **c, d, f, g, i, k, l, n** and **o. ****p* <0.05; *******p* <0.01; ********p* <0.001.

**
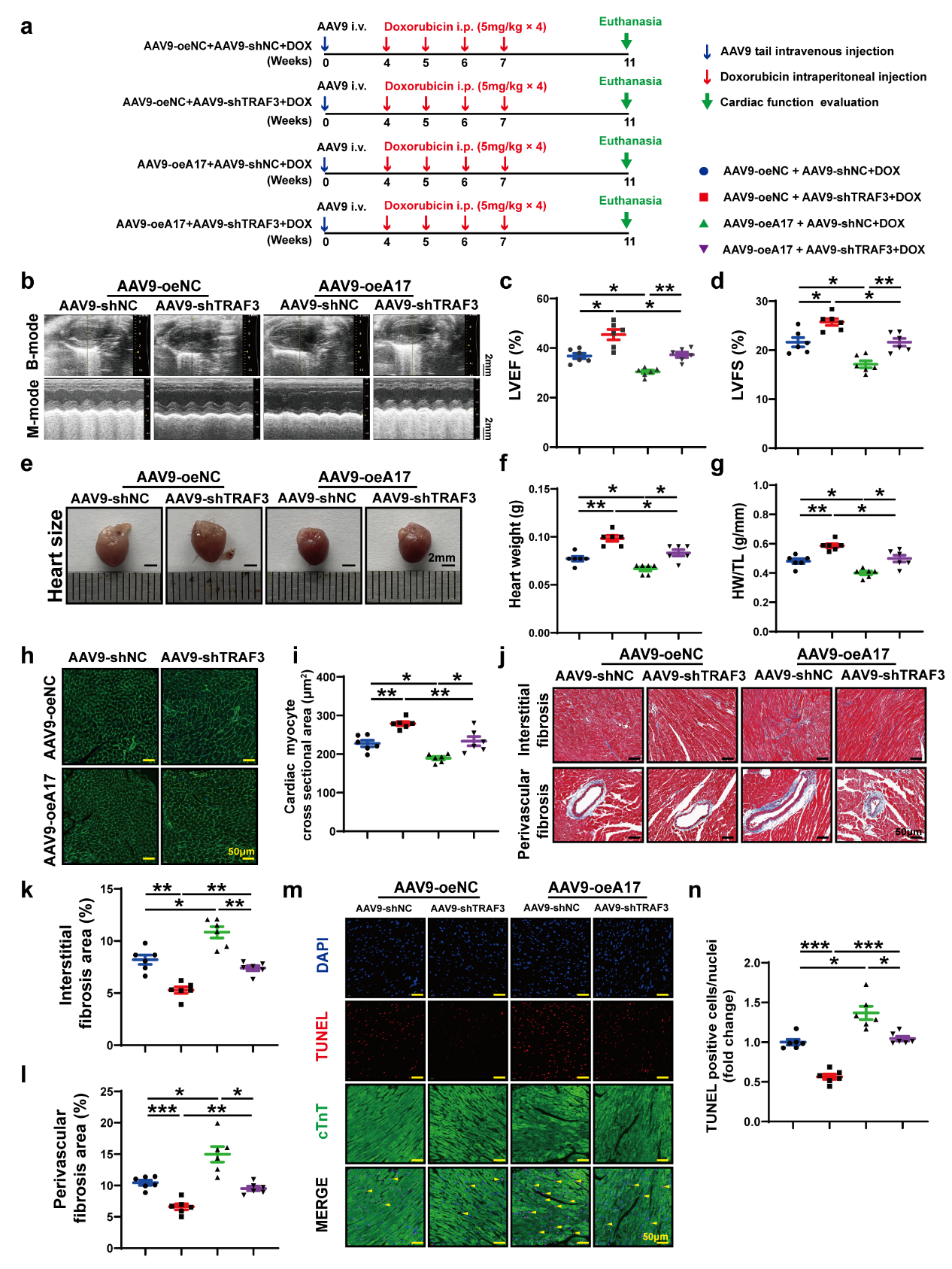
**

**Supplementary Figure 11. Echocardiographic measurements and histological staining in negative control and ADAM17-overexpressing but TRAF3-knockdown mice treated with NS or DOX. a** Experiment timeline *in vivo*. **b** Representative echocardiographic images (scale bar=2mm) showing B-mode and M-mode echocardiograms in four groups of mice. **c** Comparison of left ventricular ejection fraction (LVEF) among four groups of mice (n=6 in each group). **d** Comparison of left ventricular fractional shortening (LVFS) among four groups of mice (n=6 in each group). **e** Representative anatomical images of heart size (scale bar=2mm) in four groups of mice. **f** Comparison of heart weight among four groups of mice (n=6 in each group). **g** Comparison of heart weight/tibial length (HW/TL) ratio among four groups of mice (n=6 in each group). **h** Representative WGA staining of myocardial cross-sections (scale bar=50μm) in four groups of mice. **i** Comparison of cardiac myocyte cross-sectional area measured by WGA staining (n=6 in each group). **j** Representative Masson’s trichrome staining of myocardial interstitial and perivascular fibrosis (scale bar=50μm) in four groups of mice. **k** Comparison of interstitial fibrosis area among four groups of mice (n=6 in each group). **l** Comparison of perivascular fibrosis area among four groups of mice (n=6 in each group). **m** Representative TUNEL-positive cardiomyocyte staining (scale bar=50μm) in four groups of mice (n=6 in each group). **n** Comparison of TUNEL-positive cardiomyocyte in the myocardium among four groups of mice (n=6 in each group). Values shown were mean and SEM. One-way ANOVA were applied in **c, d, f, g, i, k, l** and **n. ****p* <0.05; *******p* <0.01; ********p* <0.001.

**
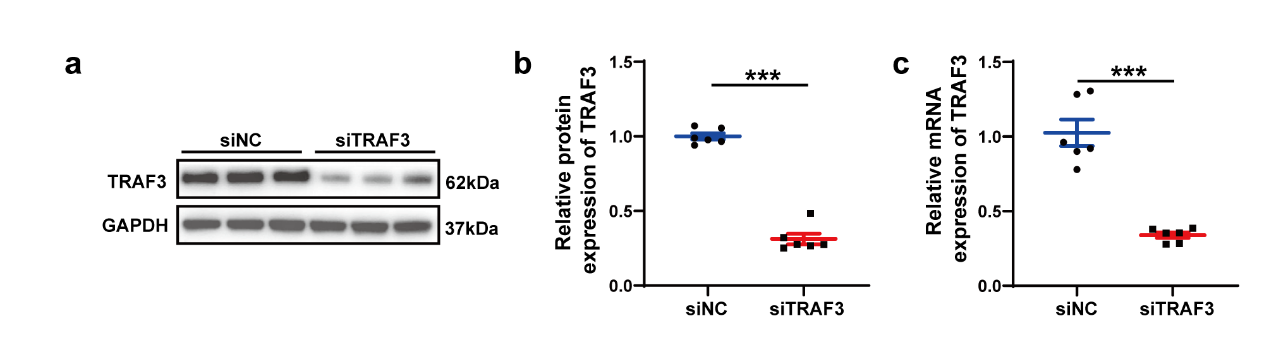
**

**Supplementary Figure 12. Efficiency of TRAF3 knockdown in NRCMs.** **a** Representative western blot images of protein expression of TRAF3 in two groups of NRCMs treated with NC-siRNA and TRAF3-siRNA (n=6 in each group). **b-c** Comparison of TRAF3 protein and mRNA expression in two groups of NRCMs treated with NC-siRNA and TRAF3-siRNA (n=6 in each group). Unpaired two-tailed Student’s t test were applied in **b** and **c.** ********p* <0.001.

**
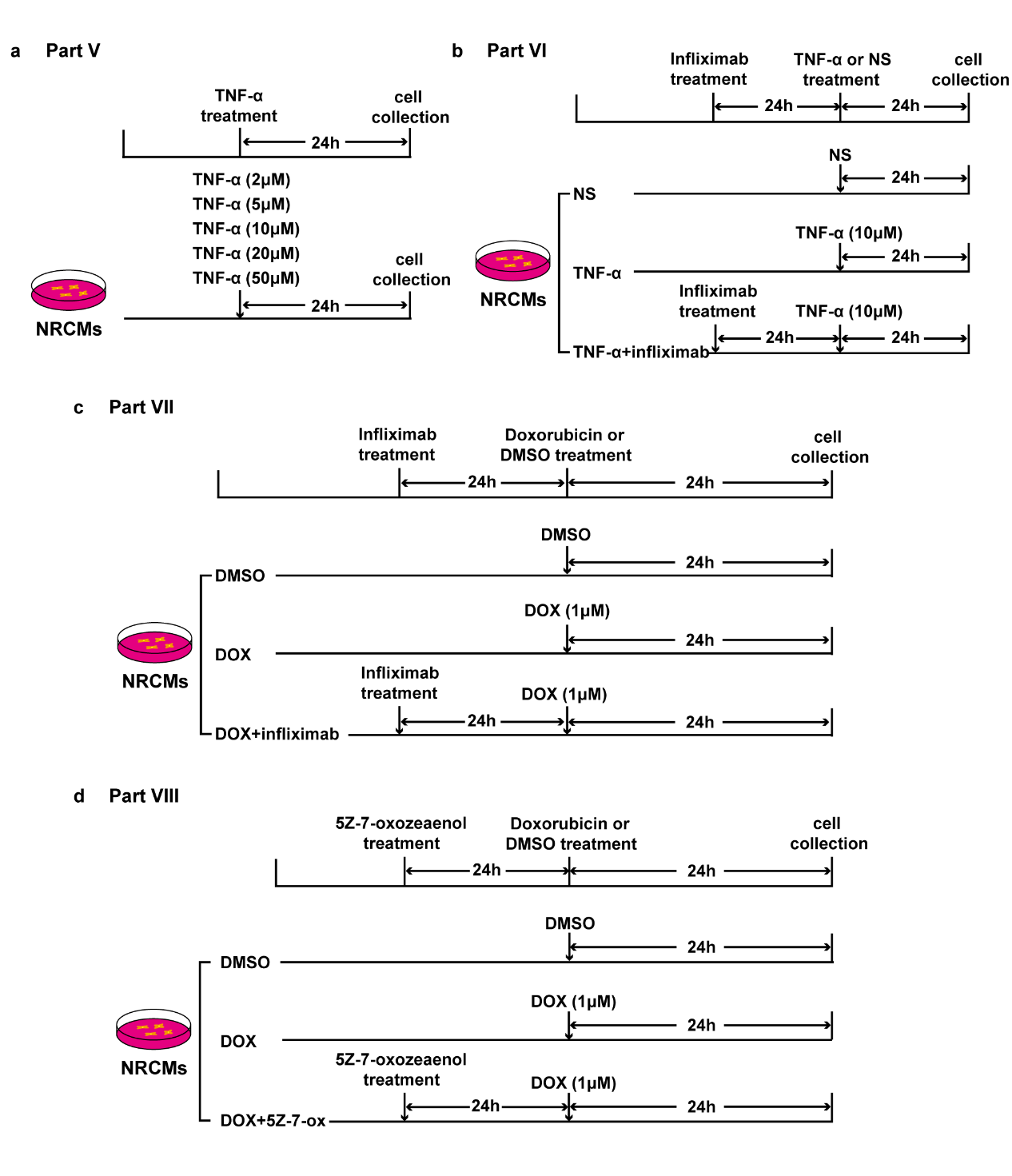
**

**Supplementary Figure 13. Experiment timeline *in vitro*.**

**
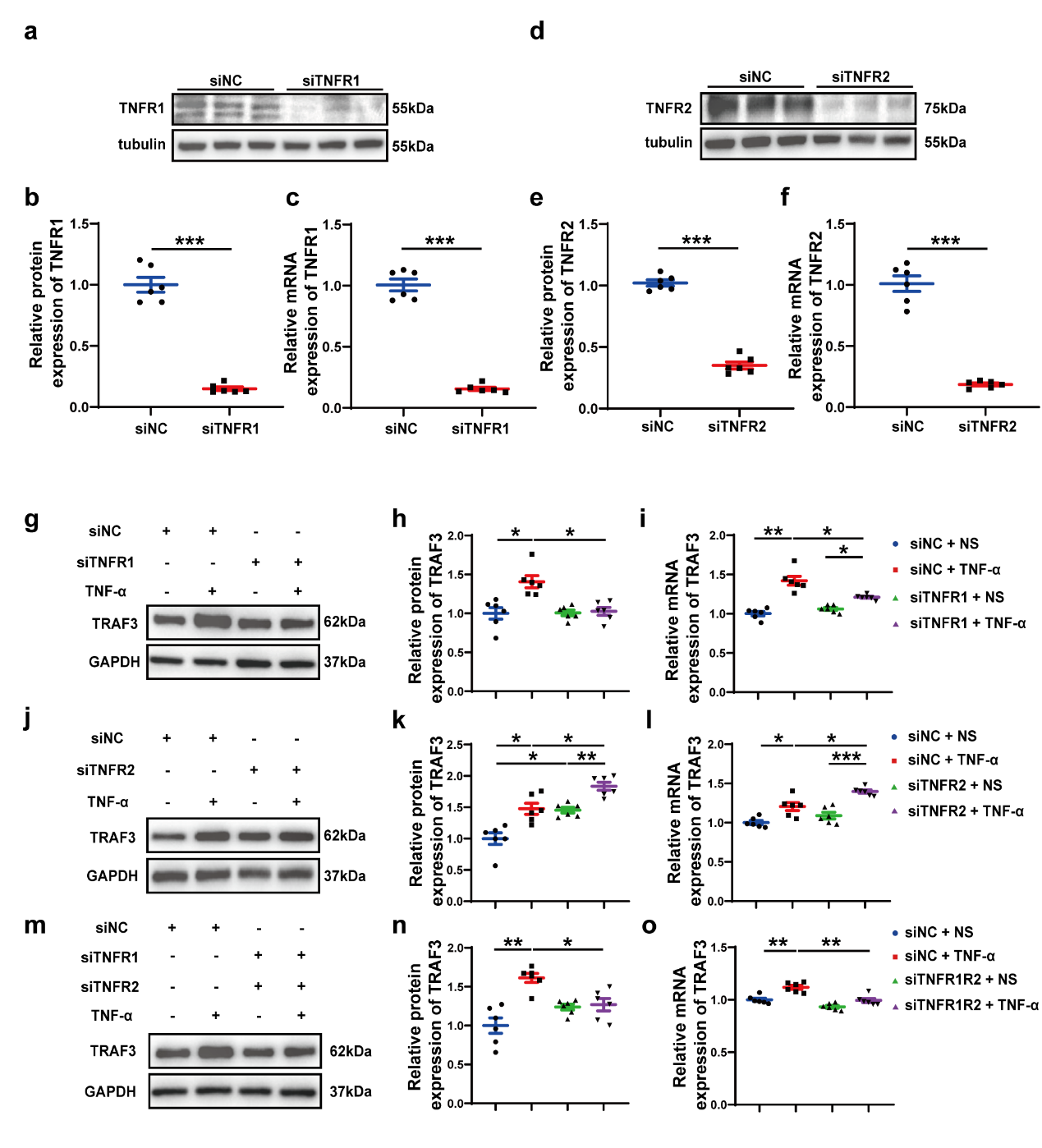
**

**Supplementary Figure 14. TNF-α enhanced TRAF3 expression via TNFR1 in** **NRCMs treated with DOX. a** Representative western blot images of protein expression of TNFR1 in two groups of NRCMs treated with NC-siRNA and TNFR1-siRNA, respectively (n=6 in each group). **b-c** Comparison of TNFR1 protein and mRNA expression in two groups of NRCMs treated with NC-siRNA and TNFR1-siRNA, respectively (n=6 in each group). **d** Representative western blot images of protein expression of TNFR2 in two groups of NRCMs treated with NC-siRNA and TNFR2-siRNA, respectively (n=6 in each group). **e-f** Comparison of TNFR2 protein and mRNA expression in two groups of NRCMs treated with NC-siRNA and TNFR2-siRNA, respectively (n=6 in each group). **g-h** Representative western blot images and comparison of protein expression of TRAF3 among four groups of NRCMs treated with siNC + NS, siNC + TNF-α, siTNFR1+NS, and siTNFR1+TNF-α, respectively (n=6 in each group). **i** Comparison of mRNA expression of TRAF3 among four groups of NRCMs treated with siNC + NS, siNC + TNF-α, siTNFR1+NS, and siTNFR1+TNF-α, respectively (n=6 in each group). **j-k** Representative western blot images and comparison of protein expression of TRAF3 among four groups of NRCMs treated with siNC + NS, siNC + TNF-α, siTNFR2 + NS, and siTNFR2 + TNF-α, respectively (n=6 in each group). **l** Comparison of mRNA expression of TRAF3 among four groups of NRCMs treated with siNC + NS, siNC + TNF-α, siTNFR2+NS, and siTNFR2+TNF-α, respectively (n=6 in each group). **m-n** Representative western blot images and comparison of protein expression of TRAF3 among four groups of NRCMs treated with siNC + NS, siNC + TNF-α, siTNFR1 + siTNFR2 (siTNFR1R2) +NS, and siTNFR1R2+TNF-α, respectively (n=6 in each group). **o** Comparison of mRNA expression of TRAF3 among four groups of NRCMs treated with siNC + NS, siNC + TNF-α, siTNFR1R2 + NS, and siTNFR1R2 + TNF-α, respectively (n=6 in each group). Values shown were mean and SEM. Unpaired two-tailed Student’s t test were applied in **b, c, e** and **f**, One-way ANOVA were applied in **h, i, k, l, n** and **o**. ******p* <0.05; *******p* <0.01; ********p* <0.001.

**
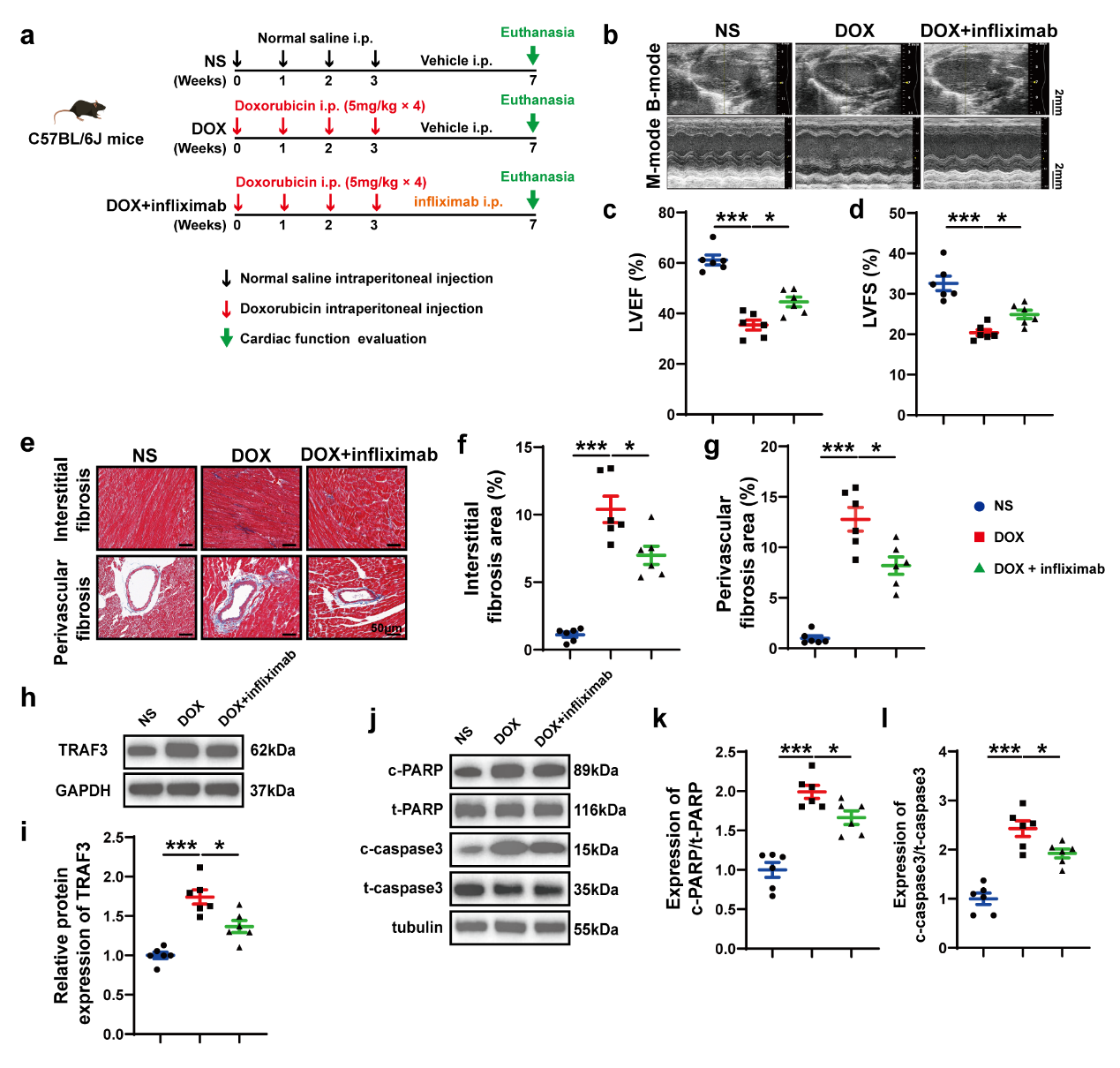
**

**Supplementary Figure 15. Echocardiographic measurements, histological staining and effects of infliximab on cardiomyocyte apoptosis in mice treated with NS or DOX. a** Experiment timeline *in vivo*. **b** Representative echocardiographic images (scale bar=2mm) showing B-mode and M-mode echocardiograms in three groups of mice. **c** Comparison of left ventricular ejection fraction (LVEF) among three groups of mice (n=6 in each group). **d** Comparison of left ventricular fractional shortening (LVFS) among three groups of mice (n=6 in each group). **e** Representative Masson’s trichrome staining of myocardial interstitial and perivascular fibrosis (scale bar=50μm) in three groups of mice. **f** Comparison of interstitial fibrosis area among three groups of mice (n=6 in each group). **g** Comparison of perivascular fibrosis area among three groups of mice (n=6 in each group). **h-i** Representative western blot images and comparison of protein expression of TRAF3 among three groups of mice (n=6 in each group). **j** Representative western blot images of PARP, cleaved PARP, caspase 3 and cleaved caspase3 expression in three groups of mice. **k** Comparison of cleaved PARP/PARP expression among three groups of mice (n=6 in each group). **l** Comparison of cleaved caspase3/caspase3 expression among three groups of mice (n=6 in each group). Values shown were mean and SEM. One-way ANOVA were applied in **c, d, f, g, i, k** and **l. ****p* <0.05; ********p* <0.001.

**
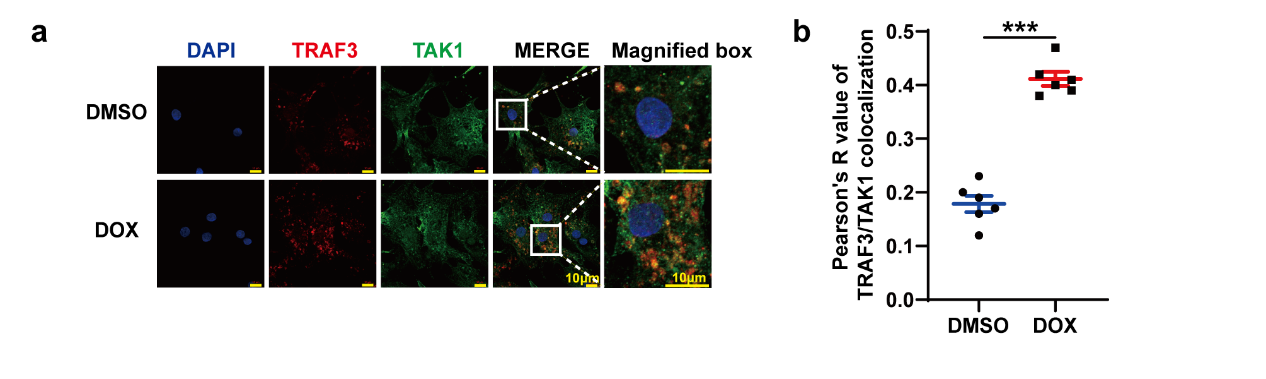
**

**Supplementary Figure 16. Colocalization of TRAF3 and TAK1 in NRCMs. a** Representative immunofluorescence staining of TRAF3 (red) and TAK1 (green) in NRCMs treated with DMSO or DOX (scale bar=10μm). **b** Quantitative analysis of colocalization of TRAF3 and TAK1 in NRCMs (n=6 in each group). Values shown were mean and SEM. Unpaired two-tailed Student’s t test were applied in **b. ******p* <0.001.


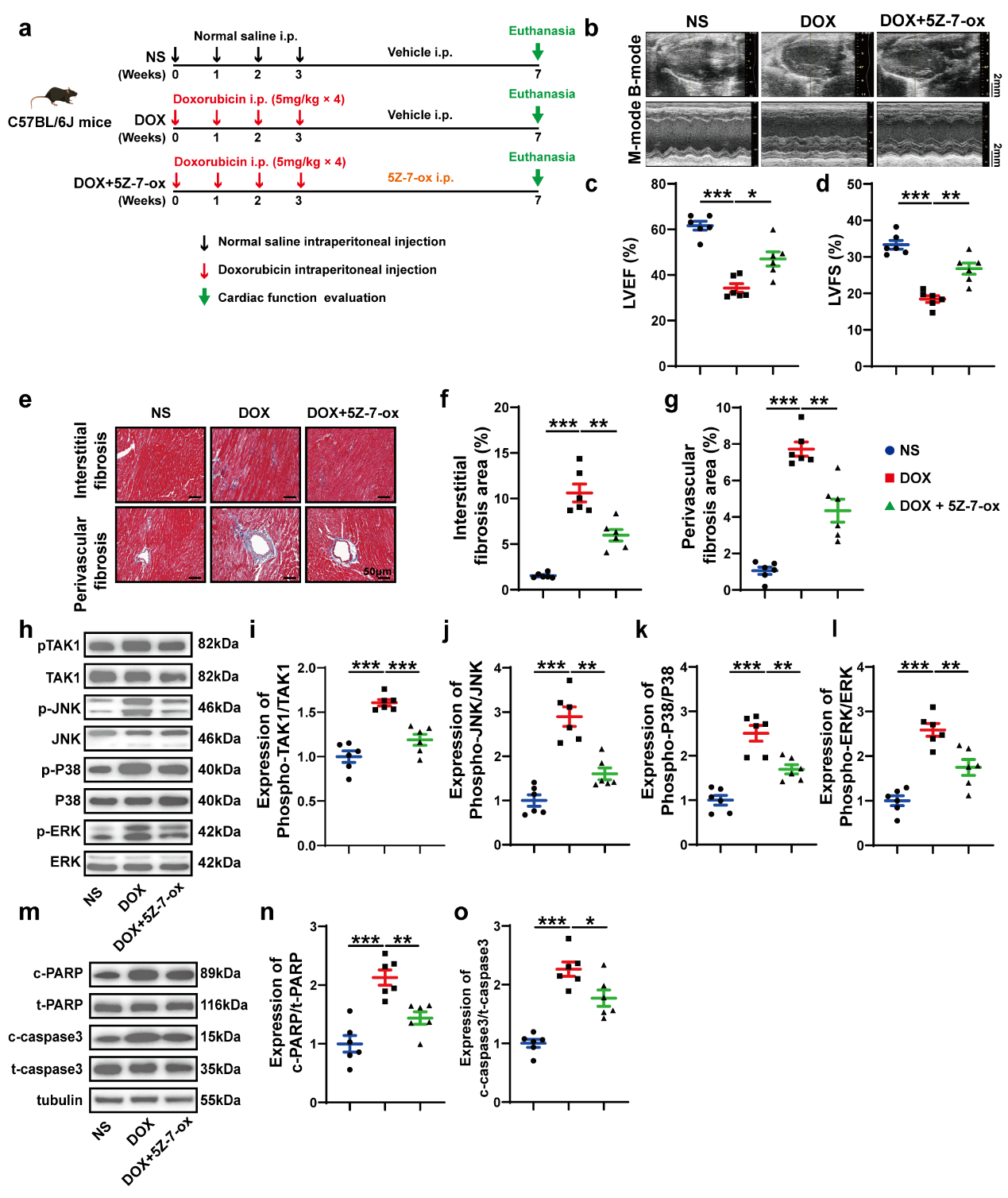


**Supplementary Figure 17. Echocardiographic measurements, histological staining and effects of 5Z-7-ox on cardiomyocyte apoptosis in mice treated with NS or DOX. a** Experiment timeline *in vivo*. **b** Representative echocardiographic images (scale bar=2mm) showing B-mode and M-mode echocardiograms in three groups of mice. **c** Comparison of left ventricular ejection fraction (LVEF) among three groups of mice (n=6 in each group). **d** Comparison of left ventricular fractional shortening (LVFS) among three groups of mice (n=6 in each group). **e** Representative Masson’s trichrome staining of myocardial interstitial and perivascular fibrosis (scale bar=50μm) in three groups of mice. **f** Comparison of interstitial fibrosis area among three groups of mice (n=6 in each group). **g** Comparison of perivascular fibrosis area among three groups of mice (n=6 in each group). **h** Representative western blot images of phosphorylated TAK1, TAK1, phosphorylated JNK, JNK, phosphorylated P38 MAPK, P38 MAPK, phosphorylated ERK and ERK among three groups of mice. **i** Comparison of protein expression of phosphorylated TAK1/TAK1 among three groups of mice (n=6 in each group). **j** Comparison of protein expression of phosphorylated JNK/JNK among three groups of mice (n=6 in each group). **k** Comparison of protein expression of phosphorylated P38 MAPK/P38 MAPK among three groups of mice (n=6 in each group). **l** Comparison of protein expression of phosphorylated ERK/ERK MAPK among three groups of mice (n=6 in each group). **m** Representative western blot images of PARP, cleaved PARP, caspase 3 and cleaved caspase3 expression in three groups of mice. **n** Comparison of cleaved PARP/PARP expression among three groups of mice (n=6 in each group). **o** Comparison of cleaved caspase3/caspase3 expression among three groups of mice (n=6 in each group). Values shown were mean and SEM. One-way ANOVA were applied in **c, d, f, g, i, j, k, l, n** and **o. ****p* <0.05; *******p* <0.01; ********p* <0.001.


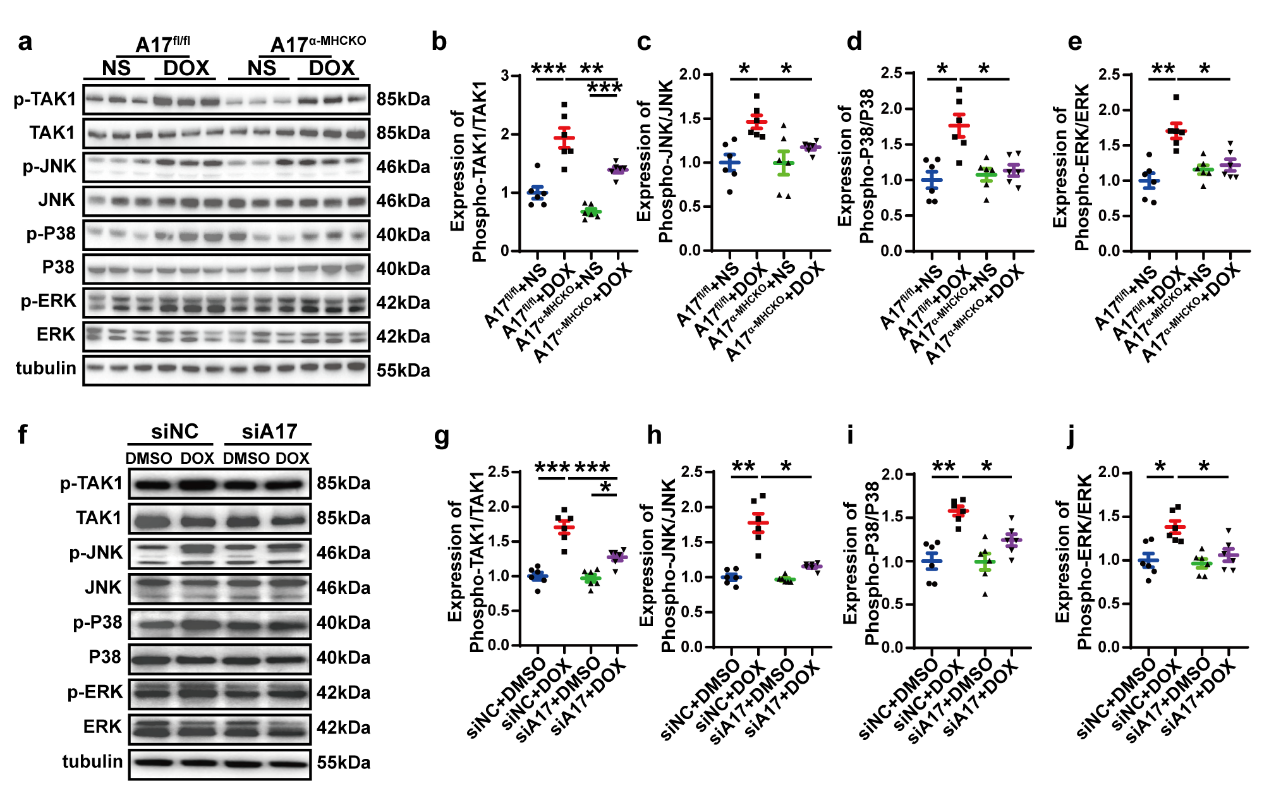


**Supplementary Figure 18. ADAM17 deficiency regulates TAK1 and MAPKs pathway activation in mice and in NRCMs treated with vehicle or DOX. a** Representative western blot images of protein expression of phosphorylated TAK1, TAK1, phosphorylated JNK, JNK, phosphorylated P38 MAPK, P38 MAPK, phosphorylated ERK and ERK in the myocardium of A17^fl/fl^+NS, A17^fl/fl^+DOX, A17^α-MHCKO^+NS and A17^α-MHCKO^+DOX groups. **b** Comparison of phosphorylated TAK1/TAK1 expression in the myocardium of four groups of mice (n=6 in each group). **c** Comparison of phosphorylated JNK/JNK expression in the myocardium of four groups of mice (n=6 in each group). **d** Comparison of phosphorylated P38 MAPK/P38 MAPK expression in the myocardium of four groups of mice (n=6 in each group). **e** Comparison of phosphorylated ERK/ERK expression in the myocardium of four groups of mice (n=6 in each group). **f** Representative western blot images of protein expression of phosphorylated TAK1, TAK1, phosphorylated JNK, JNK, phosphorylated P38 MAPK, P38 MAPK, phosphorylated ERK and ERK in four groups of NRCMs treated with siNC + DMSO, siNC + DOX, siA17+DMSO and siA17+DOX, respectively. **g** Comparison of phosphorylated TAK1/TAK1 expression among four groups of NRCMs treated with siNC + DMSO, siNC + DOX, siA17+DMSO and siA17+DOX, respectively (n=6 in each group). **h** Comparison of phosphorylated JNK/JNK expression among four groups of NRCMs treated with siNC + DMSO, siNC + DOX, siA17+DMSO and siA17+DOX, respectively (n=6 in each group). **i** Comparison of phosphorylated P38 MAPK/P38 MAPK expression among four groups of NRCMs treated with siNC + DMSO, siNC + DOX, siA17+DMSO and siA17+DOX, respectively (n=6 in each group). **j** Comparison of phosphorylated ERK/ERK expression among four groups of NRCMs treated with siNC + DMSO, siNC + DOX, siA17+DMSO and siA17+DOX, respectively (n=6 in each group). Values shown were mean and SEM. One-way ANOVA were applied in **b, c, d, e, g, h, i** and **j.** ******p* <0.05; *******p* <0.01; ********p* <0.001.


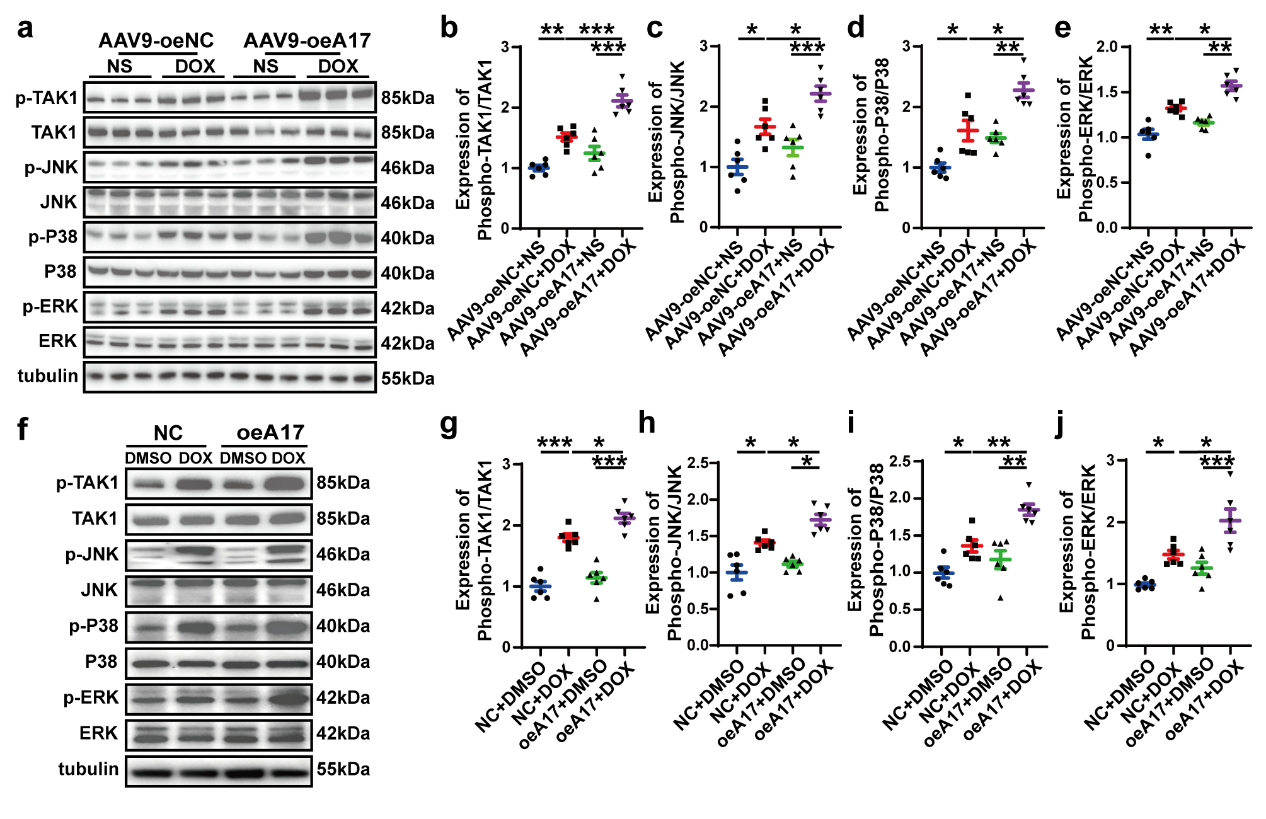


**Supplementary Figure 19. ADAM17 overexpression regulates TAK1 and MAPKs pathway activation in mice and in NRCMs treated with vehicle or DOX. a** Representative western blot images of protein expression of phosphorylated TAK1, TAK1, phosphorylated JNK, JNK, phosphorylated P38 MAPK, P38 MAPK, phosphorylated ERK and ERK in the myocardium of AAV9-oeNC+NS, AAV9-oeNC+DOX, AAV9-oeA17+NS and AAV9-oeA17+DOX groups. **b** Comparison of phosphorylated TAK1/TAK1 expression in the myocardium of four groups of mice (n=6 in each group). **c** Comparison of phosphorylated JNK/JNK expression in the myocardium of four groups of mice (n=6 in each group). **d** Comparison of phosphorylated P38 MAPK/P38 MAPK expression in the myocardium of four groups of mice (n=6 in each group). **e** Comparison of phosphorylated ERK/ERK expression in the myocardium of four groups of mice (n=6 in each group). **f** Representative western blot images of protein expression of phosphorylated TAK1, TAK1, phosphorylated JNK, JNK, phosphorylated P38 MAPK, P38 MAPK, phosphorylated ERK and ERK in four groups of NRCMs treated with NC+DMSO, NC+DOX, oeA17+DMSO and oeA17+DOX, respectively. **g** Comparison of phosphorylated TAK1/TAK1 expression in four groups of NRCMs treated with NC+DMSO, NC+DOX, oeA17+DMSO and oeA17+DOX, respectively (n=6 in each group). **h** Comparison of phosphorylated JNK/JNK expression among four groups of NRCMs treated with NC+DMSO, NC+DOX, oeA17+DMSO and oeA17+DOX, respectively (n=6 in each group). **i** Comparison of phosphorylated P38 MAPK/P38 MAPK expression among four groups of NRCMs treated with NC+DMSO, NC+DOX, oeA17+DMSO and oeA17+DOX, respectively (n=6 in each group). **j** Comparison of phosphorylated ERK/ERK expression among four groups of NRCMs treated with NC+DMSO, NC+DOX, oeA17+DMSO and oeA17+DOX, respectively (n=6 in each group). Values shown were mean and SEM. One-way ANOVA were applied in **b, c, d, e, g, h, i** and **j.** ******p* <0.05; *******p* <0.01; ********p* <0.001.


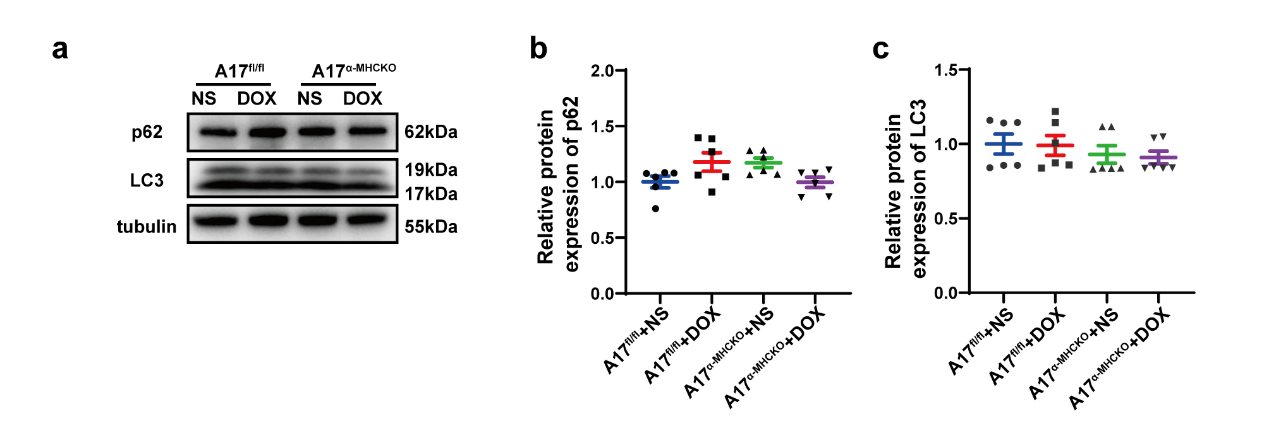


**Supplementary Figure 20. Effects of ADAM17 knockout on autophagy-related proteins in mice treated with NS or DOX. a** Representative western blot images of p62 and LC3 protein expression in the myocardium of four groups of mice. **b** Comparison of p62 protein expression among four groups of mice (n=6 in each group). **c** Comparison of LC3 expression among four groups of mice (n=6 in each group). Values shown were mean and SEM. One-way ANOVA were applied in **b** and **c.**

**Supplemental Table 1. Genotyping primers and cycling conditions for ADAM17^fl/fl^ and α-MHC-Cre mice.**

| Genotype | Sequence 5’-3’ | Primers |
| --- | --- | --- |
| ADAM17^fl/fl^ | TCC CCC AGG TAG ATT GTT TG | Forward |
|  | AGG ACC CAG GTT CAG TTC CT | Reverse |
| α-MHC-Cre | ATT TGC CTG CAT TAC CGG TC | Forward |
|  | ATC AAC GTT TTC TTT TCG G | Reverse |

α-MHC-Cre mice: alpha-myosin heavy chain-Cre mice.

The cycling conditions of A17^fl/fl^ were: 94 °C for 2 min, and 94 °C for 20 s, 65 °C for 15 s, and 68 °C for 10 s for 10 cycles, and 94 °C for 15 s, and 60 °C for 15 s, and 72 ˚C for 10 s for 28 cycles, and 72 ˚C for 2 min, and 10 ˚C HOLD. The cycling conditions of α-MHC-Cre were: 94 °C for 3 min, and 94 °C for 45 s, 57 °C for 30 s, and 72 °C for 1min for 30 cycles, and 72 ˚C for 10 min, and 20 °C HOLD.

**Supplemental Table 2. Echocardiography measurements for mice treated with NS or DOX.**

| Animal experiments | First proportion | | Second proportion | | | | Third proportion | | | |
| --- | --- | --- | --- | --- | --- | --- | --- | --- | --- | --- |
| Grouping | **NS** | **DOX** | **A17^fl/fl^**  **+NS** | **A17^fl/fl^**  **+DOX** | **A17^α-MHCKO^**  **+NS** | **A17^α-MHCKO^**  **+DOX** | **AAV9-oeNC**  **+NS** | **AAV9-oeNC**  **+DOX** | **AAV9-oeA17**  **+NS** | **AAV9-oeA17**  **+DOX** |
| LVEF (%) | **59.12±7.06** | **36.47±5.10***** | **62.30±5.18** | **37.49±3.26***** | **57.07±4.28** | **49.61±1.97###** | **59.52±4.60** | **46.78±4.70***** | **59.21±3.64** | **38.96±4.98#** |
| LVFS (%) | **29.49±3.15** | **16.34±3.37***** | **30.32±2.89** | **17.42±1.34***** | **31.70±4.17** | **21.83±1.79#** | **30.57±2.96** | **23.38±2.43***** | **31.18±2.25** | **18.63±2.06##** |
| LVIDd  (mm) | **3.44±0.20** | **3.96±0.14***** | **3.19±0.13** | **4.03±0.23***** | **3.24±0.19** | **3.61±0.10#** | **3.30±0.16** | **3.88±0.13***** | **3.57±0.20** | **4.17±0.28#** |
| LVIDs  (mm) | **2.58±0.25** | **3.00±0.12***** | **2.04±0.19** | **2.87±0.23***** | **2.10±0.20** | **2.39±0.11##** | **2.15±0.16** | **2.75±0.1***** | **2.38±0.18** | **2.97±0.33** |
| IVSd  (mm) | **0.85±0.11** | **0.68±0.07**** | **0.08±0.05** | **0.68±0.07**** | **0.81±0.05** | **0.77±0.06** | **0.87±0.11** | **0.76±0.08** | **0.88±0.15** | **0.71±0.07** |
| IVSs  (mm) | **1.03±0.11** | **0.86±0.09**** | **1.10±0.06** | **0.89±0.08**** | **1.07±0.10** | **0.99±0.09** | **1.03±0.14** | **0.89±0.10** | **1.11±0.16** | **0.88±0.11** |
| LVPWd  (mm) | **0.85±0.08** | **0.67±0.08***** | **0.83±0.11** | **0.68±0.06*** | **0.85±0.09** | **0.79±0.08** | **0.89±0.10** | **0.68±0.06***** | **0.85±0.09** | **0.62±0.04** |
| LVPWs  (mm) | **1.18±0.10** | **0.97±0.09***** | **1.17±0.12** | **0.99±0.05**** | **1.19±0.10** | **1.11±0.09** | **1.28±0.16** | **1.02±0.16*** | **1.12±0.12** | **0.98±0.06** |

**LVEF:** left ventricular ejection fraction; **LVFS:** left ventricular fractional shortening; **LVIDd**: diastolic left ventricular internal diameter; **LVIDs**: systolic left ventricular internal diameter; **IVSd**: diastolic interventricular septum; **IVSs**: systolic interventricular septum; **LVPWd**: diastolic left ventricular posterior wall; **LVPWs**: systolic left ventricular posterior wall;

In the first proportion of animal experiments, ***p*＜0.01; ****p*＜0.001 vs. NS group;

In the second proportion of animal experiments,**p*＜0.05; ***p*＜0.01; ****p*＜0.001 vs. A17^fl/fl^+NS group; #*p*＜0.05; ##*p*＜0.01; ###*p*＜0.001 vs. A17^fl/fl^+DOX group;

In the third proportion of animal experiments,**p*＜0.05; ****p*＜0.001 vs. AAV9-oeNC+NS group; #*p*＜0.05; ##*p*＜0.01 vs. AAV9-oeNC+DOX group;

**Supplemental Table 3.**

| **Antibodies** | **Source** | **Identifier** |
| --- | --- | --- |
| **Western blot** | | |
| ADAM17 (1:1000) | Abcam | Cat# ab2051 |
| Bcl2 (1:1000) | Abcam | Cat# ab32124 |
| Bax (1:1000) | Abcam | Cat# ab32503 |
| tubulin (1:1000) | Abcam | Cat# ab6046 |
| PARP (1:1000) | Cell Signaling Technology | Cat#9532 |
| cleaved PARP (1:1000) | Cell Signaling Technology | Cat#94885 |
| caspase3(1:1000) | Cell Signaling Technology | Cat#9661 |
| cleaved caspase 3 (1:1000) | Cell Signaling Technology | Cat#9664 |
| TRAF3 (1:1000) | Cell Signaling Technology | Cat#4729 |
| GAPDH (1:1000) | Cell Signaling Technology | Cat#92310 |
| p44/42 MAPK (Erk1/2) (1:1000) | Cell Signaling Technology | Cat#4695 |
| Phospho-p44/42 MAPK (Erk1/2) (1:1000) | Cell Signaling Technology | Cat#4370 |
| SAPK/JNK (1:1000) | Cell Signaling Technology | Cat#9252 |
| Phospho-SAPK/JNK (1:1000) | Cell Signaling Technology | Cat#4668 |
| p38 MAPK (1:1000) | Cell Signaling Technology | Cat#54470 |
| Phospho-p38 MAPK (1:1000) | Cell Signaling Technology | Cat#4511 |
| TAK1(1:1000) | Cell Signaling Technology | Cat#5206 |
| Phospho-TAK1(1:1000) | Cell Signaling Technology | Cat#9339 |
| C/EBPβ (1:1000) | Abcam | Cat# ab32358 |
| TNFR1(1:1000) | Cell Signaling Technology | Cat#13377 |
| TNFR2(1:1000) | Origene | TA326923 |
| DYKDDDDK-Tag | Cell Signaling Technology | Cat#14793 |
| Myc-Tag | Cell Signaling Technology | Cat#3946 |
| **Immunofluorescence** | | |
| cTnT (1:100) | Abcam | Cat#ab8295 |
| cTnT (1:100) | Proteintech | Cat#15513-1-AP |
| Vimentin (1:100) | Cell Signaling Technology | Cat#5741 |
| CD31(1:100) | Abcam | Cat#ab222783 |
| TRAF3(1:100) | Proteintech | Cat#18099-1-AP |
| TAK1(1:100) | Proteintech | Cat#67707-1-Ig |
| DYKDDDDK-Tag (1:100) | Cell Signaling Technology | Cat#14793 |
| TRAF3(1:100) | Proteintech | Cat#66310-1-Ig |
| **Reagents** | **Source** | **Identifier** |
| Doxorubicin | MCE | HY-15142 |
| TNF-α | MCE | HY-P70571 |
| 5z-7-oxozeaenol | MCE | HY-12686 |
| 5z-7-oxozeaenol | Sigma | O9890-1MG |
| infliximab | MCE | HY-P9970 |

**Supplemental Table 4. Primers used for RT-PCR analysis.**

| Gene | Sequence 5’-3’ | Primers |
| --- | --- | --- |
| *Adam17* (Mus) | AGGACGTAATTGAGCGATTTTGG | Forward |
|  | TGTTATCTGCCAGAAACTTCCC | Reverse |
| *β-actin* (Mus) | CACTGTGCCCATCTACGA | Forward |
|  | GTAGTCTGTCAGGTCCCG | Reverse |
| *Adam17* (Rattus) | GGCCCTTTGAAGAGGTGAGG | Forward |
|  | CATGTGAAGGCCAAAACCCC | Reverse |
| *Bax* (Rattus) | ACCAAAGAAGCTGAGCGAGTG | Forward |
|  | TCCACATCAGCAATCATCCTCT | Reverse |
| *Bcl2* (Rattus) | TGGCCTTCTTTGAGTTCGGT | Forward |
|  | GTTCCACAAAGGCATCCCAGC | Reverse |
| *Traf3* (Rattus) | GTGGTGTCCTGCCCTCATAA | Forward |
|  | GGCATTGACACACTCGGACA | Reverse |
| *β-actin* (Rattus) | CCACACCCGCCACCAGTTCG | Forward |
|  | TACAGCCCGGGGAGCATCGT | Reverse |
| *Tnfr1*(Rattus)  *Tnfr2*(Rattus) | CCAAGTGCCACAAAGGAACC  GGTTCTGCGAAGCTGTAAAGG  CTGTGCGGCAGGCATGTTTA  CCACCTGGTCATCACTACAGG | Forward  Reverse  Forward  Reverse |
| *Cebpβ(Rattus)* | TTCTACTACGAGCCCGACTG  GCGAAGAGGTCGGAAAGGA | Forward  Reverse |
