# Supplementary material for: Cardiomyocyte-specific knockout of ADAM17 alleviates doxorubicin-induced cardiomyopathy via inhibiting TNFα–TRAF3–TAK1–MAPK axis
Source: Signal Transduct Target Ther. 2024 Oct 16;9:273. doi: 10.1038/s41392-024-01977-z (PMC11480360; doi:10.1038/s41392-024-01977-z)

Fig.1i

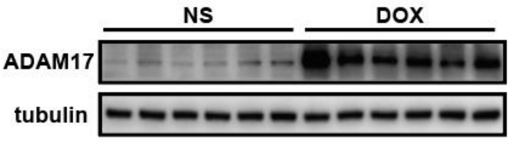

ADAM17

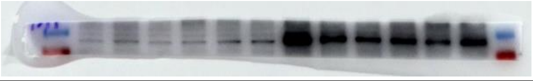

tubulin

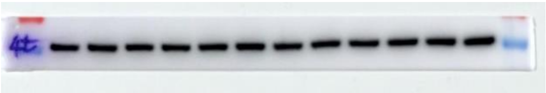

Fig.4c

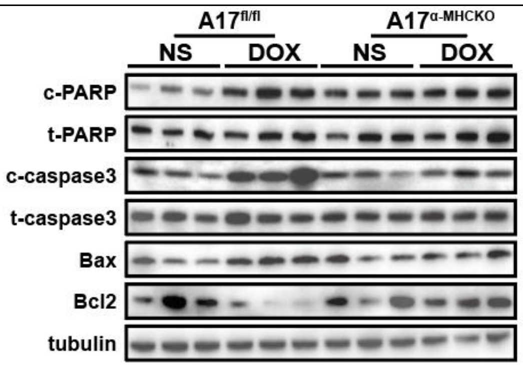

c-PARP and t-PARP

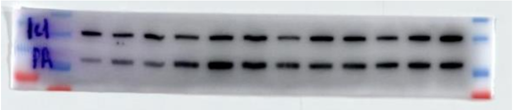

c-caspase3

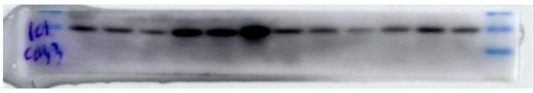

t-caspase3

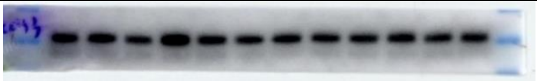

Bax

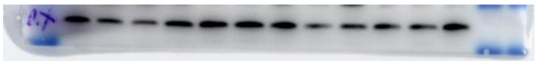

Bcl2

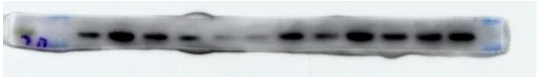

tubulin

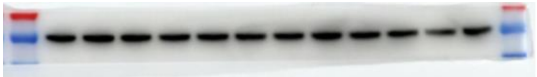

Fig.4i

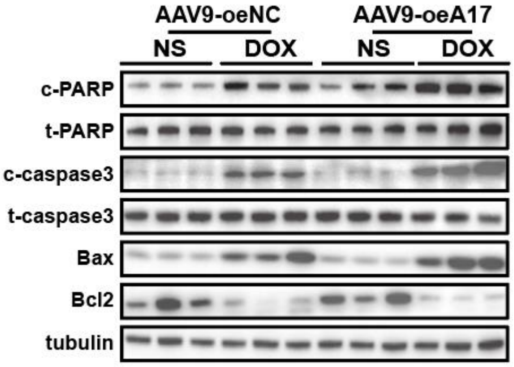

c-PARP

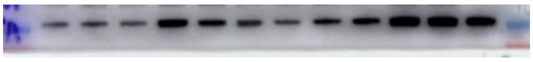

t-PARP

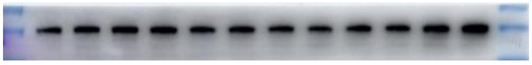

c-caspase3

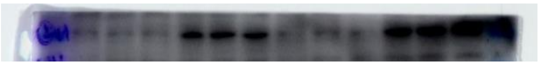

t-caspase3

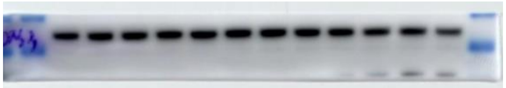

	Bax
	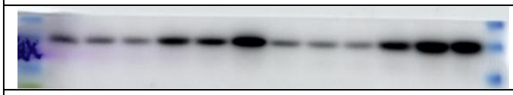
	Bcl2
	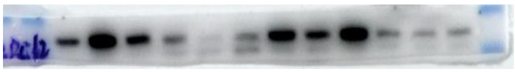
	tubulin
	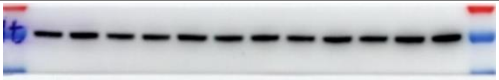
**Fig.5c**	
	c-PARP
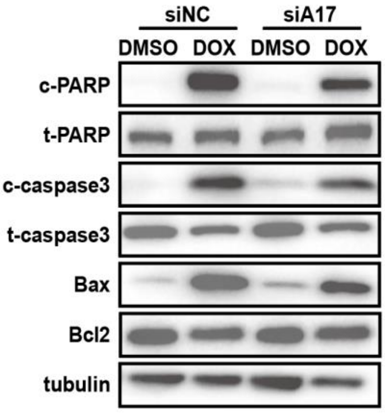	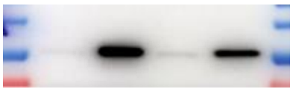
	t-PARP
	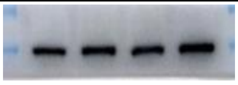
	c-caspase3
	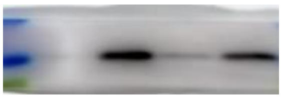
	t-caspase3
	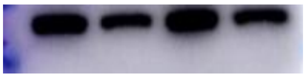
	Bax
	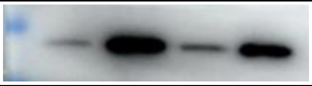
	Bcl2
	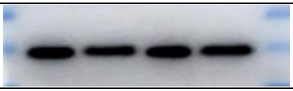
	tubulin
	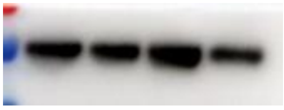
**Fig.5i**	
	c-PARP
	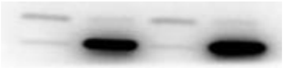
	t-PARP
	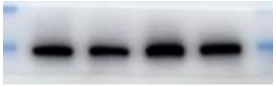
	c-caspase3

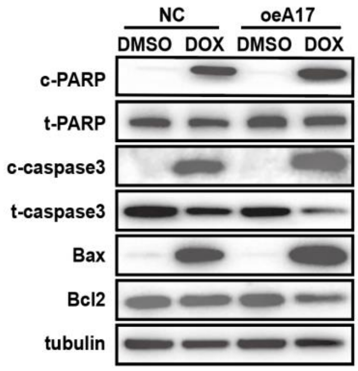

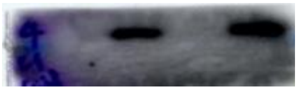

t-caspase3

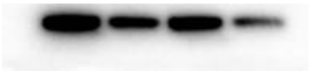

Bax

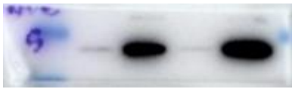

Bcl2

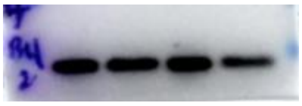

tubulin

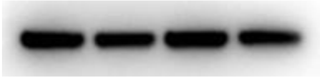

Fig.6c

TRAF3

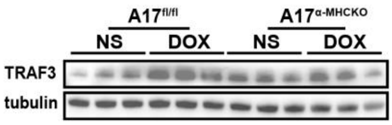

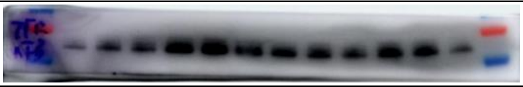

tubulin

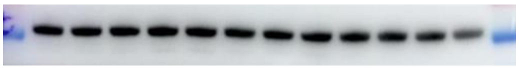

Fig.6e

TRAF3

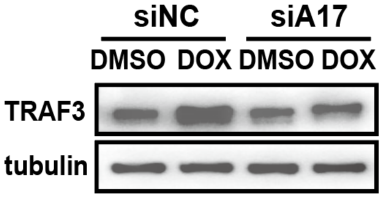

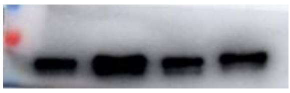

tubulin

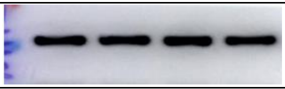

Fig.6g

c-PARP

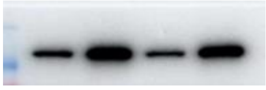

t-PARP

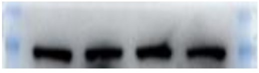

c-caspase3

<table>
<tr><td rowspan="5">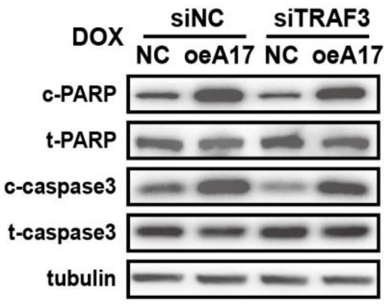
</td><td>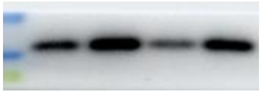</td></tr>
<tr><td>t-caspase3</td></tr>
<tr><td>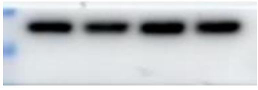</td></tr>
<tr><td>tubulin</td></tr>
<tr><td>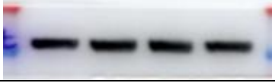</td></tr>
<tr><td colspan="2">Fig.6n</td></tr>
<tr><td></td><td>TRAF3</td></tr>
<tr><td rowspan="3">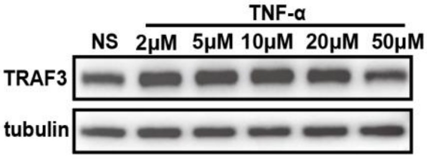
</td><td>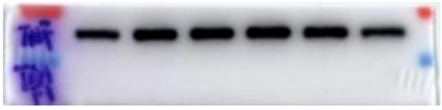</td></tr>
<tr><td>TRAF3</td></tr>
<tr><td>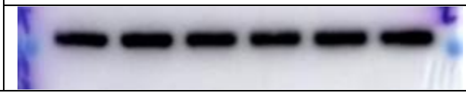</td></tr>
<tr><td colspan="2">Fig.7a</td></tr>
<tr><td></td><td>TRAF3</td></tr>
<tr><td rowspan="3">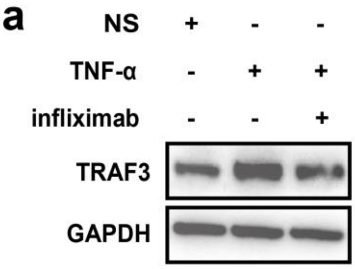
</td><td>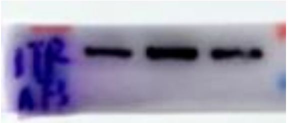</td></tr>
<tr><td>GAPDH</td></tr>
<tr><td>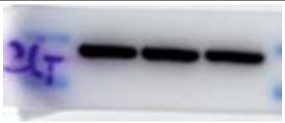</td></tr>
<tr><td colspan="2">Fig.7d</td></tr>
<tr><td></td><td>TRAF3</td></tr>
<tr><td rowspan="3">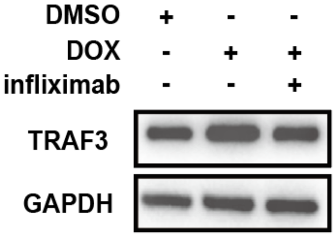
</td><td>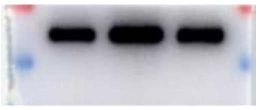</td></tr>
<tr><td>GAPDH</td></tr>
<tr><td>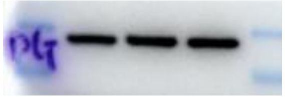</td></tr>
<tr><td colspan="2">Fig.7g</td></tr>
<tr><td></td><td>c-PARP and t-PARP</td></tr>
<tr><td rowspan="2"></td><td>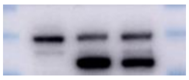</td></tr>
<tr><td>c-caspase3</td></tr>
</table>

<table>
<tr><td rowspan="5">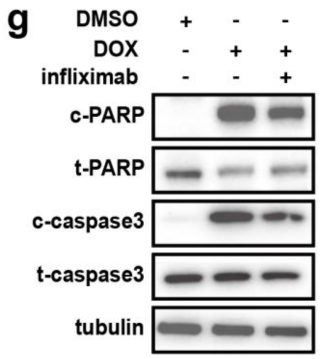
</td><td>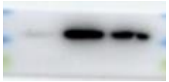</td></tr>
<tr><td>t-caspase3</td></tr>
<tr><td>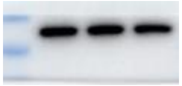</td></tr>
<tr><td>tubulin</td></tr>
<tr><td>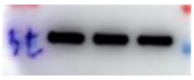</td></tr>
<tr><td colspan="2">Fig.7j</td></tr>
<tr><td></td><td>TAK1</td></tr>
<tr><td rowspan="5">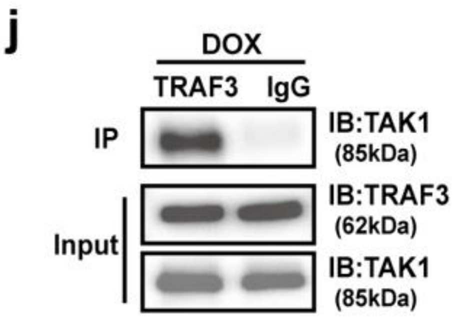
</td><td>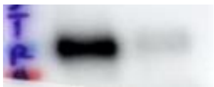</td></tr>
<tr><td>TRAF3</td></tr>
<tr><td>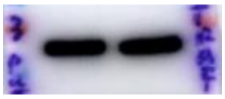</td></tr>
<tr><td>TAK1</td></tr>
<tr><td>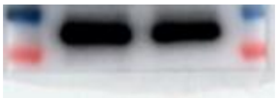</td></tr>
<tr><td colspan="2">Fig.7k</td></tr>
<tr><td></td><td>TRAF3</td></tr>
<tr><td rowspan="5">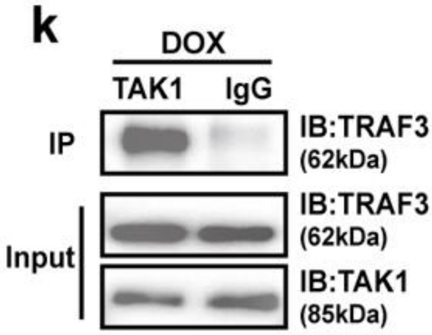
</td><td>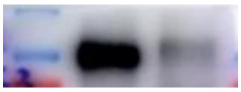</td></tr>
<tr><td>TRAF3</td></tr>
<tr><td>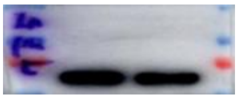</td></tr>
<tr><td>TAK1</td></tr>
<tr><td>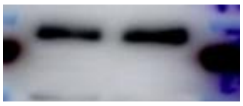</td></tr>
<tr><td colspan="2">Fig.7l</td></tr>
<tr><td></td><td>Flag</td></tr>
<tr><td rowspan="2"></td><td>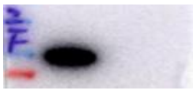</td></tr>
<tr><td>Flag</td></tr>
</table>

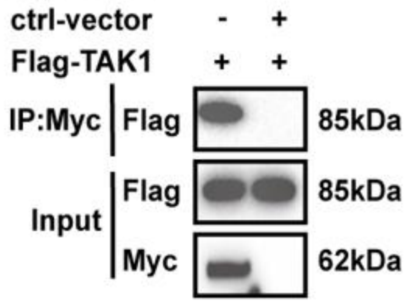

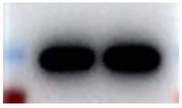

Myc

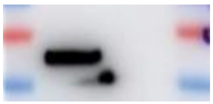

Fig.7m

Myc

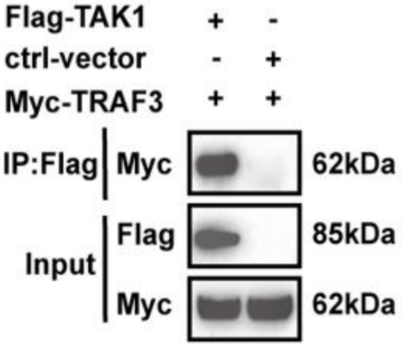

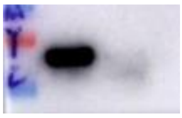

Flag

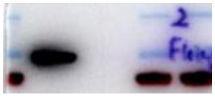

Myc

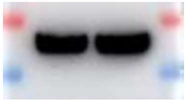

Fig.7n

p-TAK1

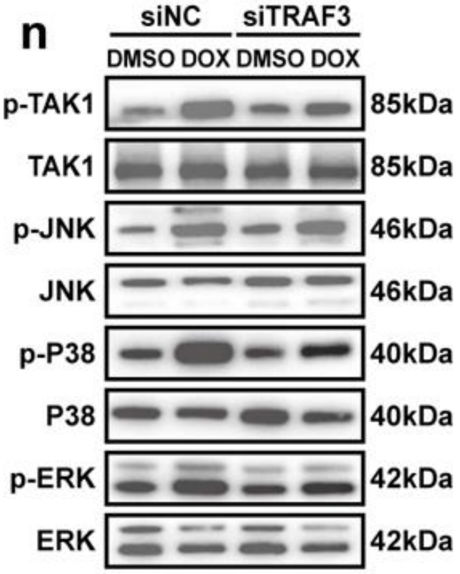

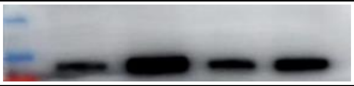

TAK1

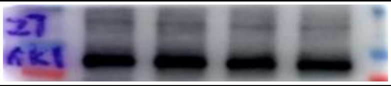

p-JNK

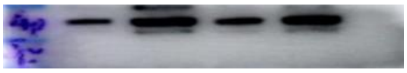

JNK

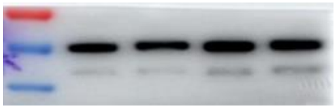

p-P38

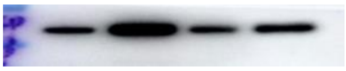

P38

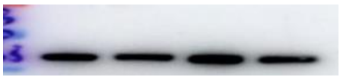

p-ERK

	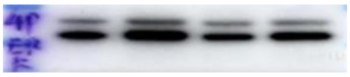
	ERK
	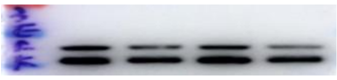
Fig.7s	
	p-TAK1
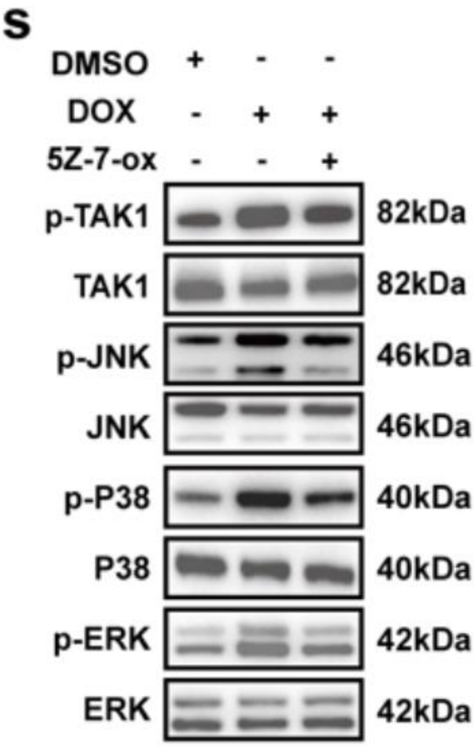	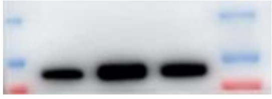
	TAK1
	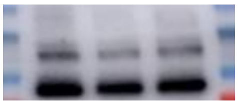
	p-JNK
	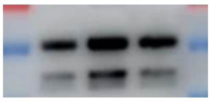
	JNK
	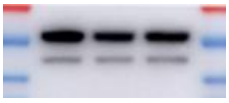
	p-P38
	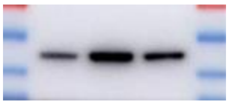
	P38
	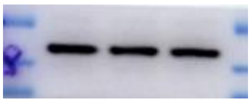
	p-ERK
	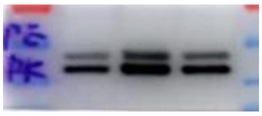
	ERK
	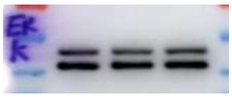
Fig.7x	
	c-PARP
	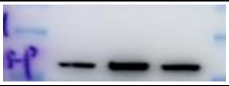

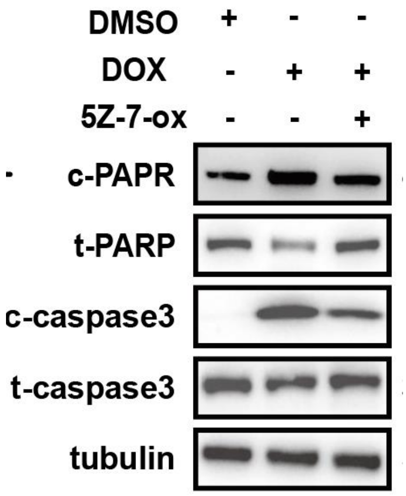

t-PARP

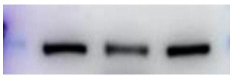

c-caspase3

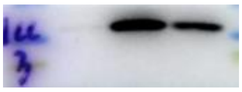

t-caspase3

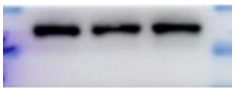

tubulin

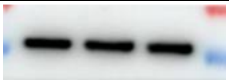

Supplementary Fig.2c

ADAM17

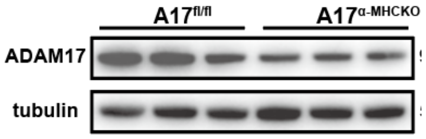

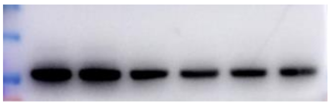

tubulin

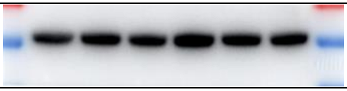

Supplementary Fig.3b

ADAM17

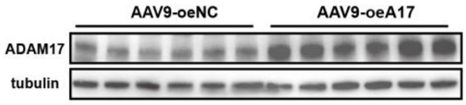

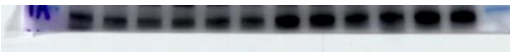

tubulin

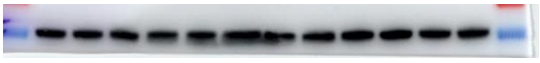

Supplementary Fig.3g

ADAM17

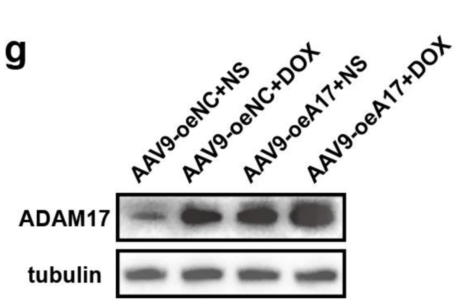

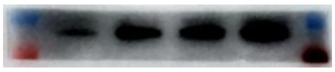

tubulin

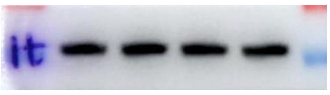

Supplementary Fig.4a

ADAM17

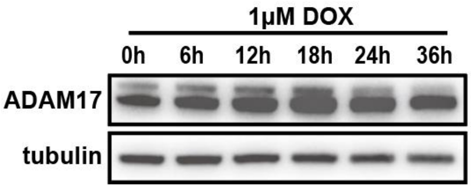

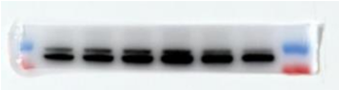

tubulin

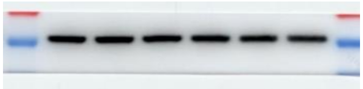

Supplementary Fig.4e

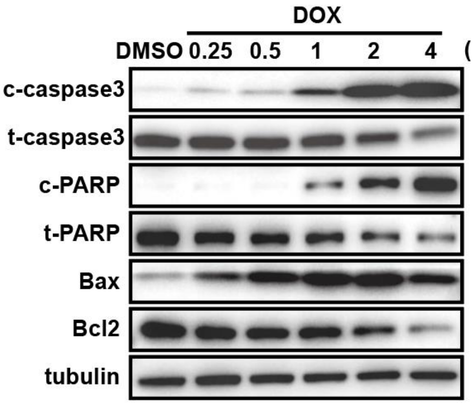

c-caspase3

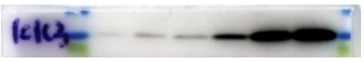

t-caspase3

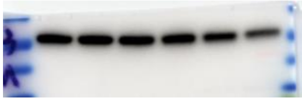

c-PARP and t-PARP

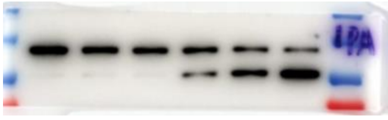

Bax

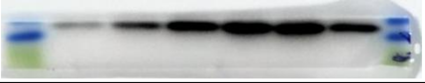

Bcl2

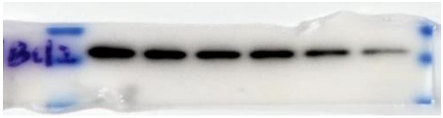

tubulin

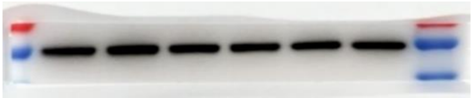

Supplementary Fig.4k

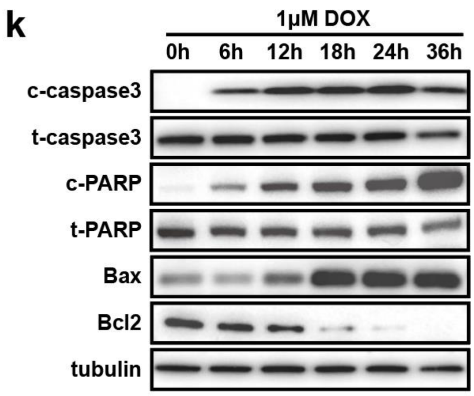

c-caspase3

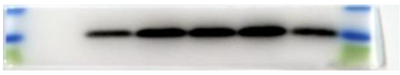

t-caspase3

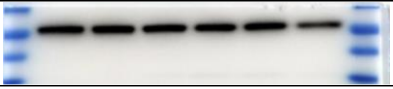

c-PARP and t-PARP

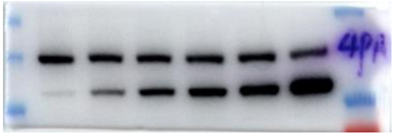

Bax

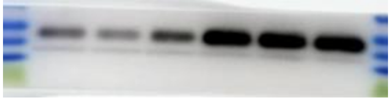

Bcl2

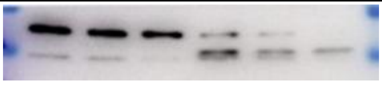

tubulin

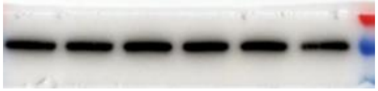

Supplementary Fig.6a

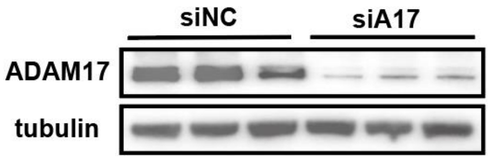

ADAM17

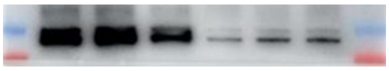

tubulin

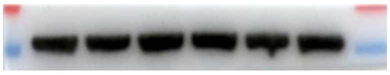

Supplementary Fig.6e

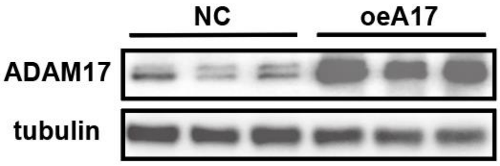

ADAM17

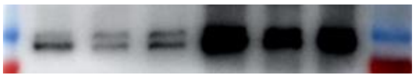

tubulin

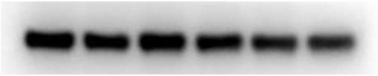

Supplementary Fig.7c

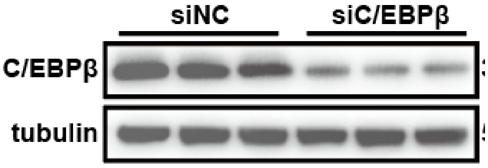

C/EBPβ

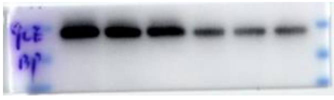

tubulin

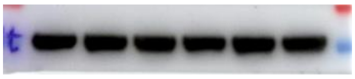

Supplementary Fig.7f

C/EBPβ

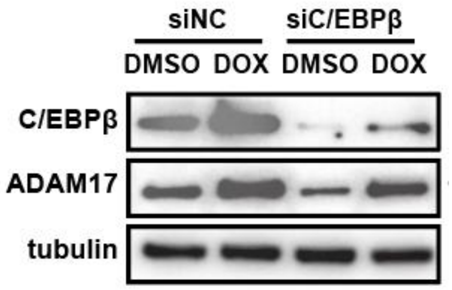

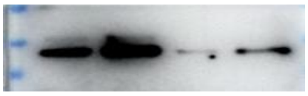

ADAM17

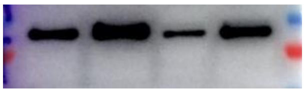

tubulin

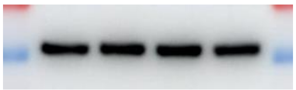

Supplementary Fig.8c

TRAF3

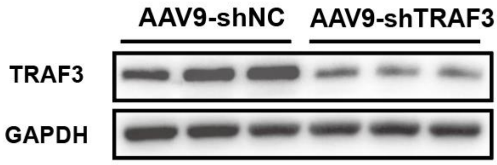

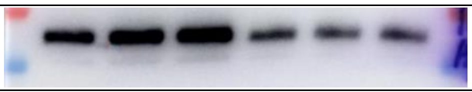

GAPDH

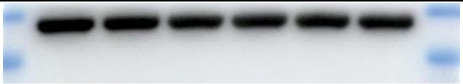

Supplementary Fig.8f

TRAF3

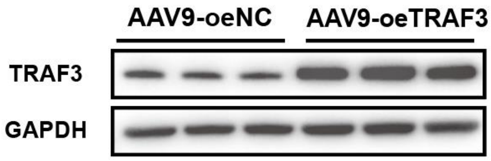

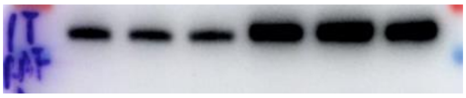

GAPDH

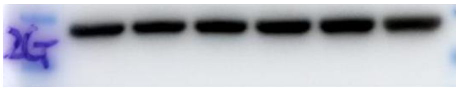

Supplementary Fig.12a

TRAF3

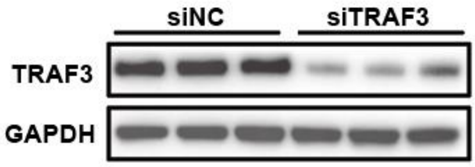

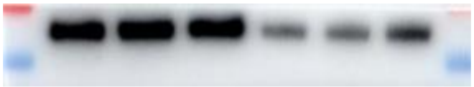

GAPDH

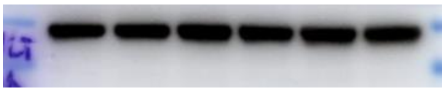

Supplementary Fig.14a

TNFR1

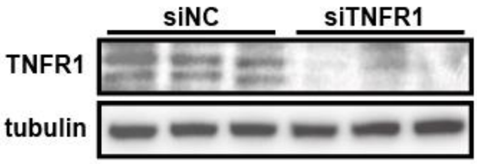

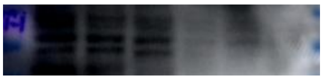

tubulin

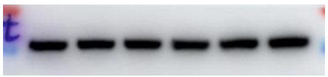

<table>
<tr><td colspan="2">Supplementary Fig.14d</td></tr>
<tr><td></td><td>TNFR2</td></tr>
<tr><td rowspan="3">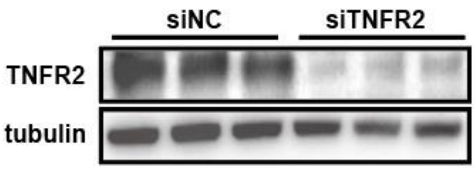
</td><td>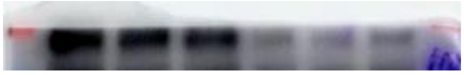</td></tr>
<tr><td>tubulin</td></tr>
<tr><td>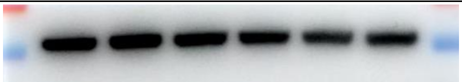</td></tr>
<tr><td colspan="2">Supplementary Fig.14g</td></tr>
<tr><td></td><td>TRAF3</td></tr>
<tr><td rowspan="3">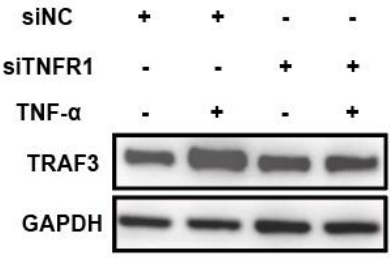
</td><td>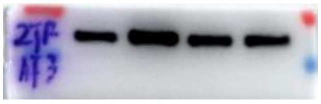</td></tr>
<tr><td>GAPDH</td></tr>
<tr><td>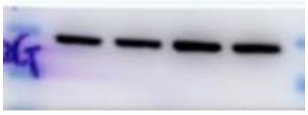</td></tr>
<tr><td colspan="2">Supplementary Fig.14j</td></tr>
<tr><td></td><td>TRAF3</td></tr>
<tr><td rowspan="3">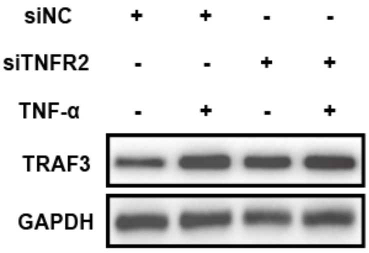
</td><td>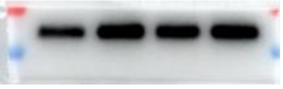</td></tr>
<tr><td>GAPDH</td></tr>
<tr><td>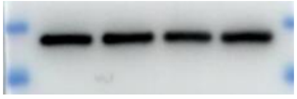</td></tr>
<tr><td colspan="2">Supplementary Fig.14m</td></tr>
<tr><td></td><td>TRAF3</td></tr>
<tr><td rowspan="3">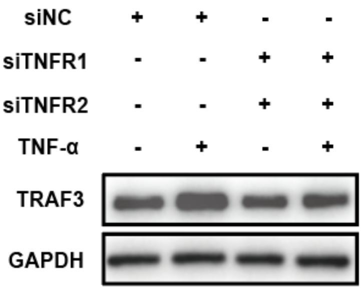
</td><td>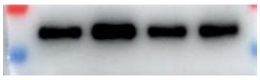</td></tr>
<tr><td>GAPDH</td></tr>
<tr><td>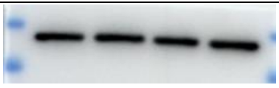</td></tr>
<tr><td colspan="2">Supplementary Fig.15h</td></tr>
<tr><td></td><td>TRAF3</td></tr>
<tr><td rowspan="2"></td><td>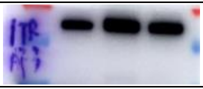</td></tr>
<tr><td>GAPDH</td></tr>
</table>

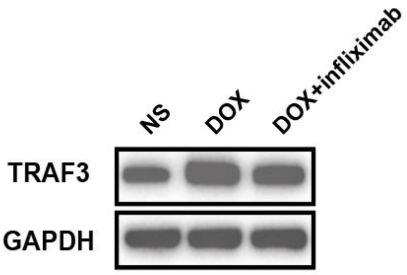	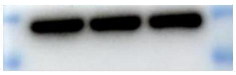
**Supplementary Fig.15j**	
	**c-PARP**
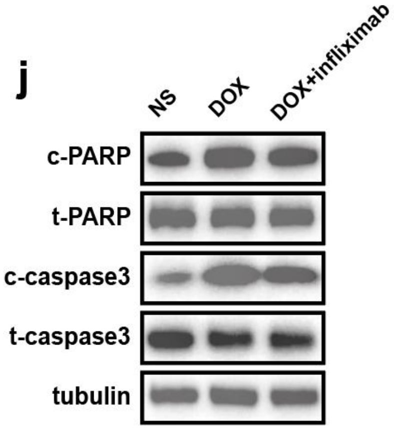	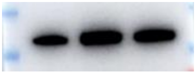
	**t-PARP**
	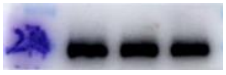
	**c-caspase3**
	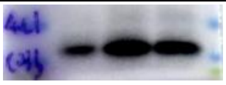
	**t-caspase3**
	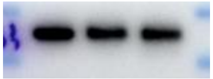
	**tubulin**
	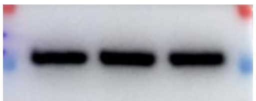
**Supplementary Fig.17h**	
	**p-TAK1**
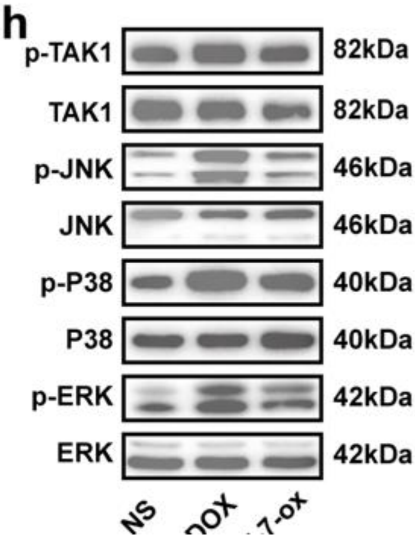	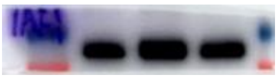
	**TAK1**
	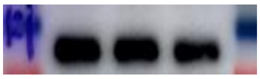
	**p-JNK**
	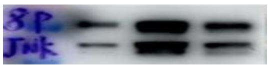
	**JNK**
	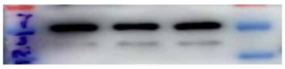
	**p-P38**
	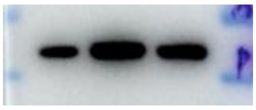
	**P38**

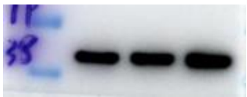

p-ERK

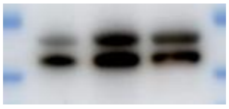

ERK

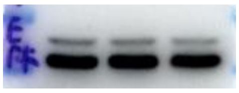

Supplementary Fig.17m

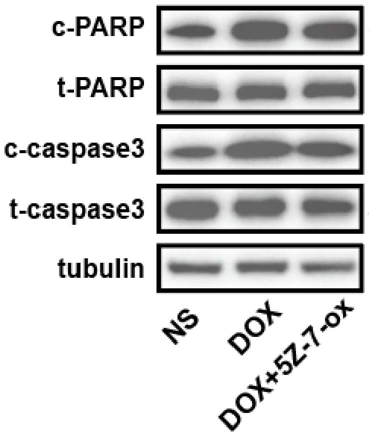

c-PARP

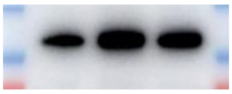

t-PARP

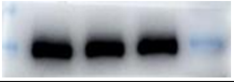

t-caspase3

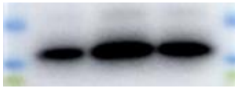

t-caspase3

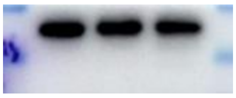

tubulin

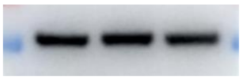

Supplementary Fig.18a

p-TAK1

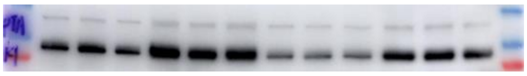

TAK1

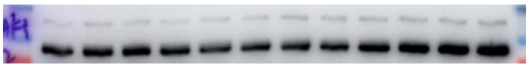

p-JNK

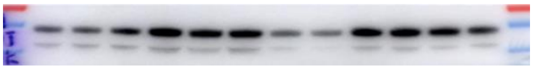

JNK

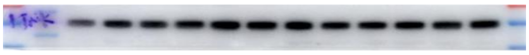

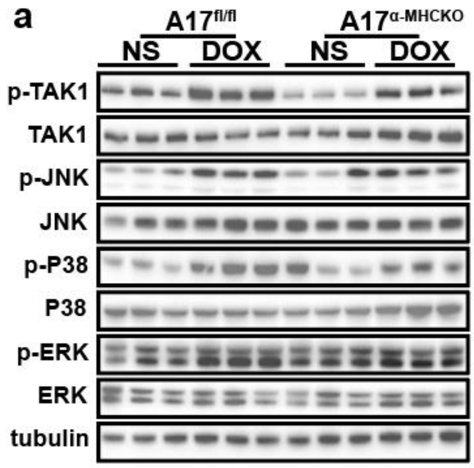

p-P38

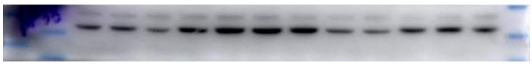

P38

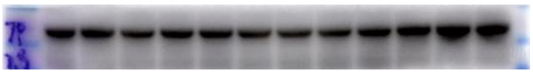

p-ERK

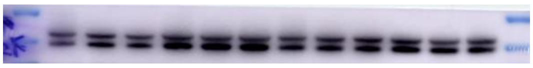

ERK

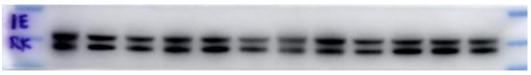

tubulin

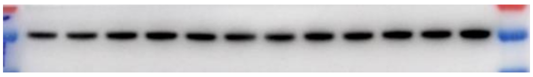

Supplementary Fig.18f

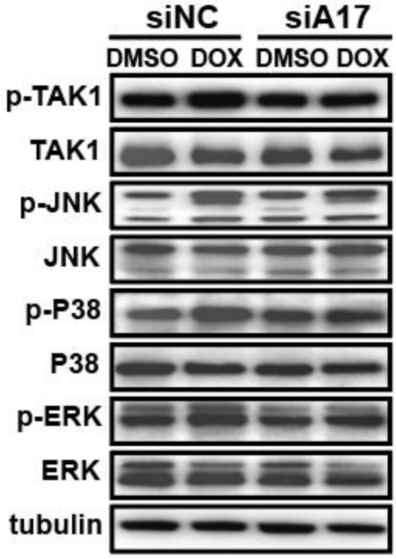

p-TAK1

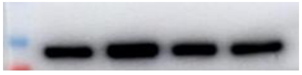

TAK1

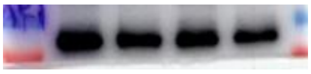

p-JNK

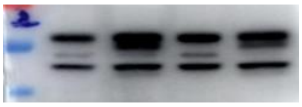

JNK

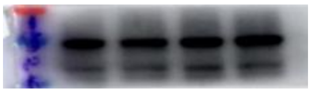

p-P38

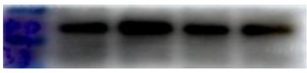

P38

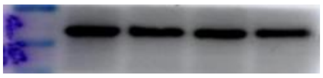

p-ERK

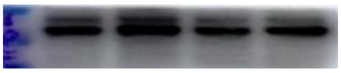

ERK

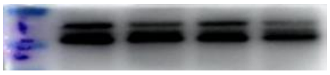

tubulin

	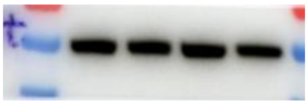
**Supplementary Fig.19a**	
	p-TAK1
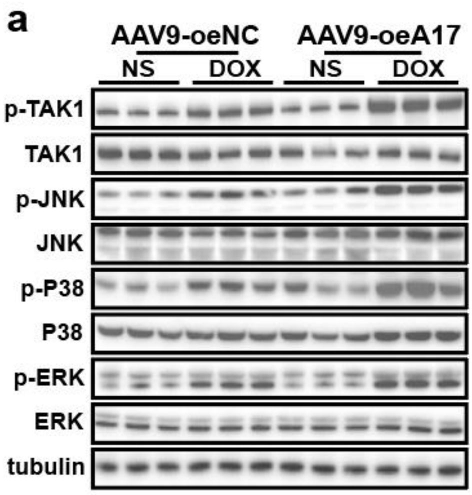	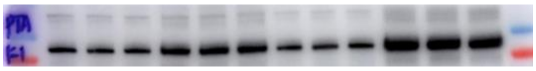<br>TAK1<br>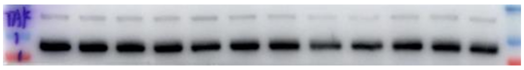<br>p-JNK<br>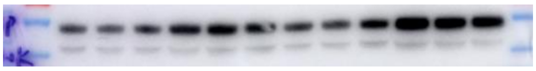<br>JNK<br>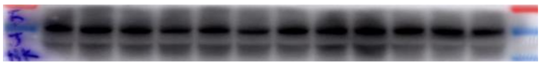<br>p-P38<br>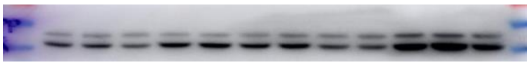<br>P38<br>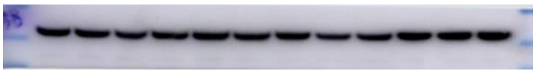<br>p-ERK<br>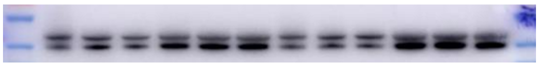<br>ERK<br>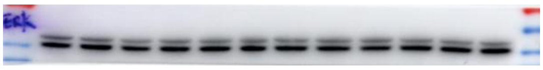<br>tubulin<br>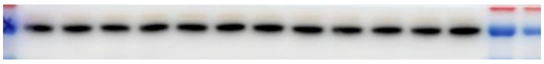
**Supplementary Fig.19f**	
	p-TAK1
	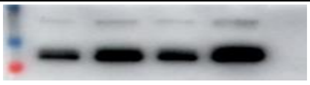<br>TAK1<br>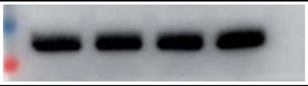<br>p-JNK<br>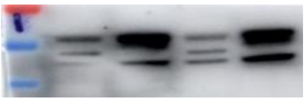<br>JNK

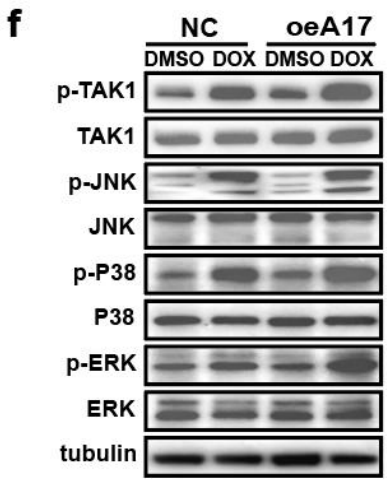

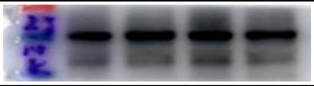

p-P38

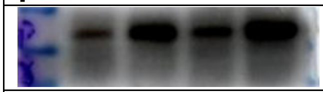

P38

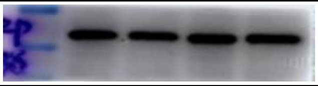

p-ERK

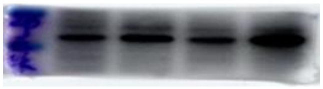

ERK

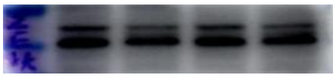

tubulin

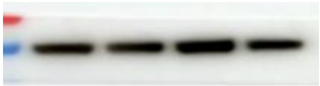

Supplementary Fig.20a

P62

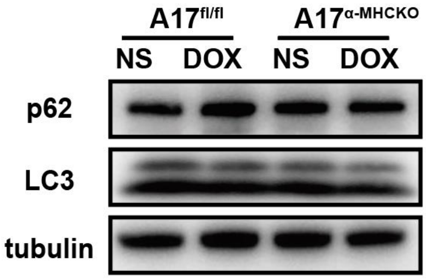

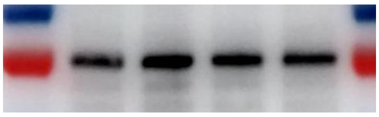

LC3

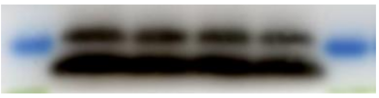

tubulin

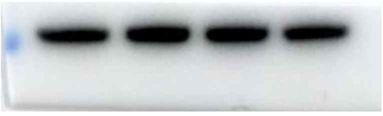

Supplement: Supplementary file 2 — western blot raw images [file 41392_2024_1977_MOESM2_ESM.pdf]
